# Synergy of Theory, NMR, and Rotational Spectroscopy to Unravel Structural Details of d‐Altroside Puckering and Side Chain Orientation

**DOI:** 10.1002/chem.202502358

**Published:** 2025-11-10

**Authors:** Donatella Loru, Clemens Lütjohann, Christian Näther, Thisbe K. Lindhorst, Melanie Schnell, Alexander A. Auer

**Affiliations:** ^1^ Deutsches Elektronen‐Synchrotron DESY Notkestr. 85 22607 Hamburg Germany; ^2^ Institute of Physical Chemistry Christiana Albertina University of Kiel Max‐Eyth‐Straße 1 24118 Kiel Germany; ^3^ Otto Diels Institute of Organic Chemistry Christiana Albertina University of Kiel Otto‐Hahn‐Platz 3–4 24118 Kiel Germany; ^4^ Institute of Inorganic Chemistry Christiana Albertina University of Kiel Max‐Eyth‐Straße 2 24118 Kiel Germany; ^5^ Max‐Planck‐Institut für Kohlenforschung Kaiser‐Wilhelm‐Platz 1 Mülheim an der Ruhr 45470 Deutschland

**Keywords:** ab initio calculations, carbohydrate conformation, conformational search, density functional theory, rotational spectroscopy

## Abstract

The conformational characteristics of carbohydrates significantly affect their chemical and biological properties; however, the conformational analysis of sugar rings (puckers) is hampered by a methodological deficit. Here, we show that the combination of NMR spectroscopy, electronic structure theory calculations, and rotational spectroscopy can provide a deep insight into the conformational degrees of freedom of sugars. Combining electronic structure theory methods and global conformational search algorithms can efficiently yield an overview and free energy ranking for thousands of possible conformers of a given monosaccharide. Using NMR spectra, a fit of computed and measured spin–spin coupling constants allows to qualitatively and quantitatively assign the ring puckers in a dynamic mixture. However, a gap between theory and experiment remains, due to the difficulty to accurately describe solvent effects in electronic structure calculations. Here, we overcome this gap by applying rotational spectroscopy, as it yields highly accurate structural data in the gas phase. This not only allows to benchmark computational data and demonstrate the accuracy that can be reached by structure prediction methods, but also yields valuable information about the side chain conformation in saccharides. We provide a detailed investigation of d‐altrose derivatives, which exhibit conformational flexibility regarding both side chains and ring puckers.

## Graphical abstract

We combine electronic structure theory calculations, NMR, and rotational spectroscopy to study ring and side chain conformations of altrosides. The calculations identify low‐energy structures among thousands of conformers. Fitting computed and experimental coupling constants reveals conformer mixtures in solution. Rotational spectroscopy benchmarks the calculations and provides information on side chain orientations.

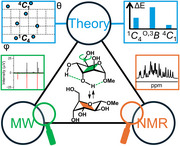

## 1 Introductions

The advent of AlphaFold^[^ ^1^, ^2^ ^]^ has revolutionised the field of protein structure prediction, with the calculation of conformations now occupying a central position in scientific discourse.^[^ ^3^ ^]^ Also, in glycobiology, the accurate prediction of the 3D structure of both monosaccharide rings as well as of oligosaccharides is crucial in the comprehension of their biological function.^[^ ^4^, ^5^, ^6^, ^7^, ^8^ ^]^ Nevertheless, while AlphaFold has already demonstrated its ability to predict protein folding with remarkable accuracy, the calculation of carbohydrate conformations is still in its adolescence.

The analysis of conformational equilibria of glycans is particularly demanding, as carbohydrate ring conformations, often called sugar puckers, are impacted by the biochemical surroundings as well as by functionalization. Since the significance of carbohydrate conformations has been increasingly recognized both in glycobiology^[^ ^4^, ^5^, ^6^, ^7^, ^8^, ^9^, ^10^, ^11^, ^12^, ^13^ ^]^ and glycochemistry^[^ ^14^, ^15^, ^16^, ^17^, ^18^ ^]^ the vital necessity for the development of precise prediction methodologies was reinforced. In the quest for rationalizing how structural modifications and glycoside puckering are related, a robust and unambiguous assignment of the conformation is of fundamental importance – yet it is only the starting point in exploring what actually influences the relative stability of a given conformer.

In contemporary conformational analysis, computational simulations are indispensable for an accurate interpretation of experimental data.^[^ ^4^, ^19^, ^20^, ^21^, ^22^, ^23^ ^]^ For the analysis of carbohydrate conformations in solution, NMR spectroscopy can be employed, as ^1^H,^1^H spin–spin coupling constants are dependent on the dihedral angle of vicinal protons according to the well‐known Karplus relation.^[^ ^24^ ^]^ However, dynamic conformational equilibria are largely inaccessible to standard NMR analysis as the interconversion of different ring conformers occurs on timescales that are significantly faster than NMR measurements.^[^ ^25^ ^]^ Consequently, the coupling constants (and also the chemical shifts) of conformational mixtures are obtained as an average.

For the prediction of conformer structures, a variety of computational approaches at different levels of theory have been employed over the past few decades, ranging from classical force field and molecular dynamics simulations (MD)^[^ ^23^, ^26^, ^27^, ^28^, ^29^, ^30^ ^]^ up to density functional theory (DFT)^[^ ^31^, ^32^, ^33^ ^]^ and coupled cluster^[^ ^4^, ^34^ ^]^ electronic structure methods. Mayes et al. emphasised that a thorough conformational search is a key element of conformation prediction, enabling the identification of all low‐energy puckering conformations.^[^ ^4^ ^]^ We previously employed the CREST conformational search and demonstrated that the fit of computed and experimental data at higher levels of theory yielded precise results for the conformational dynamics of diamino‐xylopyranosides.^[^ ^34^ ^]^ However, we recognized that DFT and even post‐HF calculations with implicit solvation are not sufficiently accurate to unambiguously determine the most stable carbohydrate conformers by their energy alone. We speculated that high‐level ab initio methods — which typically yield accuracies of a few kJ/mol in relative energies — do not allow to quantitatively predict the relative stabilities of xyloside conformers due to solvent effects as described by the implicit solvation model.

Note that while combining conformer‐sensitive experimental NMR data (like *J* coupling constants) with computed values allows to assign a composition of puckering conformers to the experimental data, this approach will not yield reliable information about side‐chain orientation.

Hence, there is still a gap between theory and experiment: On the one hand, it remains to be demonstrated that it is indeed the treatment of solvation effects that is limiting the predictive power of computed results. On the other hand, while calculations yield fully optimized structures, it is difficult to obtain detailed information on side‐chain structure and dynamics from NMR data.

This gap could be resolved by applying even more complex explicit and dynamic solvation models, which, however, often come with prohibitive computational cost and/or limited accuracy. Alternatively, spectroscopy could be applied if a) it yields accurate and detailed structural information, and b) solvent effects could be excluded. In fact, for the most unbiased comparison and the most conclusive benchmarking, it is best to join theory and gas‐phase experiments, as numerous studies in other areas of spectroscopy have impressively demonstrated.^[^ ^35^, ^36^ ^]^

Combining both advantages, rotational spectroscopy stands out for its unparalleled resolution, enabling the accurate differentiation of even subtle structural variations. It excels in providing precise information on structural features, such as ring puckering, side‐chain arrangements, and the complex network of intramolecular interactions.^[^ ^37^, ^38^, ^39^, ^40^, ^41^, ^42^, ^43^ ^]^ In a rotational spectrum, the spectral signatures of multiple conformers of the same molecule may coexist. Since these signatures depend strictly on the atomic mass distribution of the molecule, they serve as molecular fingerprints, allowing for unambiguous conformational differentiation. These capabilities make rotational spectroscopy particularly effective for studying the preferred conformations of carbohydrates in an environment free of solvent effects.^[^ ^37^, ^38^, ^39^, ^40^, ^41^, ^42^, ^43^ ^]^ As it circumvents complications associated with solvents, the technique can serve as a benchmark for computational approaches.

Note that rotational spectroscopy has already proven successful in the conformational analysis of carbohydrates: Cocinero and coworkers used the technique to uncover the conformation of d‐ribose.^[^ ^37^ ^]^ Similarly, Alonso and coworkers demonstrated their ability to unambiguously identify the different rotamers of d‐glucose arising from varying orientations of the hydroxy groups.^[^ ^39^ ^]^ More recently, rotational spectroscopy was used to investigate the conformational landscape of methylated sugars.^[^ ^43^ ^]^

**Figure 1:**
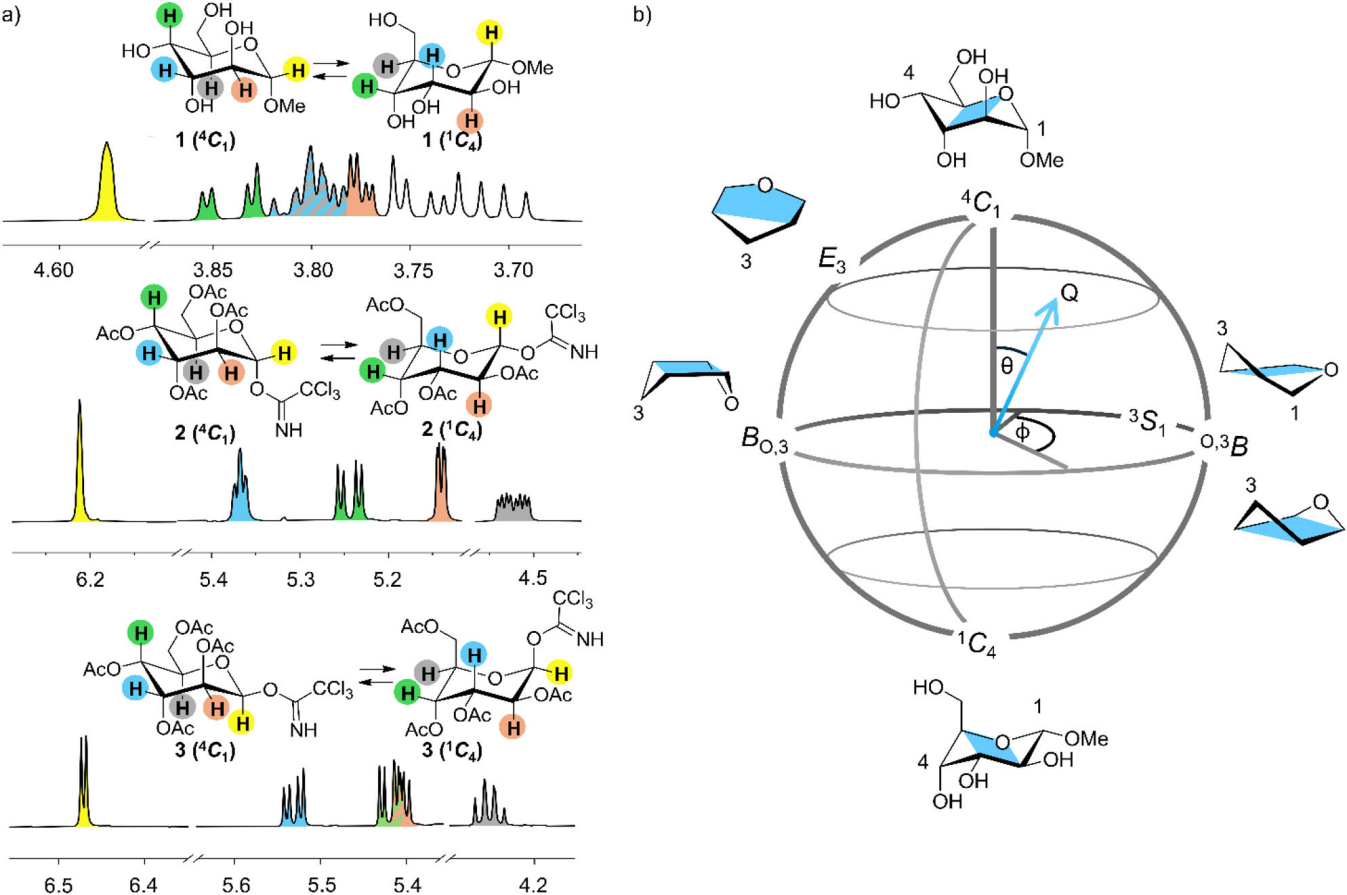
a) In this work, we investigate the conformational properties of three d‐altrose derivatives, namely methyl α‐d‐altropyranoside (**1**), *O*‐(α‐altropyranosyl) trichloroacetimidate **2**, and *O*‐(β‐altropyranosyl) trichloroacetimidate **3,** which were used in a previous publication for the synthesis of altrobiosides.^[^ ^46^ ^]^ In order to determine the composition of the conformer mixtures in solution, we use spin–spin coupling constants obtained from the ^1^H NMR spectra of **1**–**3**. b) The ring conformation of pyranoses can be described mathematically by two angles θ and Φ and a puckering amplitude Q according to Cremer and Pople.^[^ ^49^ ^]^ As a result, a spherical coordinate system (also referred to as conformational sphere) is obtained in which the contrary chair conformations, ^4^ *C*_1_ and ^1^ *C*_4_, are located at the North and South poles, respectively. Consequently, conformational equilibria between the ^4^ *C*_1_ and ^1^ *C*_4_ conformations follow distinct itineraries,^[^ ^4^ ^]^ populating other conformers, for example ^O,3^ *B*, on the way.

Hence, in this work, we leverage the synergistic combination of NMR spectroscopy, rotational spectroscopy, and theory to comprehensively characterize the conformational landscape of d‐altrose derivatives. These sugars serve as ideal model compounds for conformational analysis, as the ring inversion energy in d‐altrose is particularly low and thus conformational equilibria between the complementary chair conformations ^4^ *C*_1_ and ^1^ *C*_4_ can be observed (Figure 1a).^[^ ^26^, ^33^, ^44^, ^45^ ^]^ Here, we investigate methyl α‐d‐altropyranoside **1** and the α‐ and β‐altropyranosyl trichloroacetimidates **2** and **3,** which were previously used in the synthesis of methyl altrobiosides. Their NMR analysis indicated intriguing conformational properties (Figure 1a).^[^ ^46^ ^]^

The presented conformational analysis exploits an extended computational workflow with respect to our previously published approach^[^ ^34^ ^]^ by including a global optimization tool (namely the GOAT algorithm as implemented in ORCA 6.0^[^ ^47^, ^48^ ^]^). The new scheme is based on a conformational ensemble obtained using GOAT, which is then refined at various levels of theory, yielding a map of practically all stable conformers. The final step involves a DLPNO‐CCSD(T) energy calculation and DFT and MP2 simulations of free energy corrections, NMR parameters, and dipole moments. By integrating theory with solution‐phase information from NMR and the high‐resolution capabilities of gas‐phase rotational spectroscopy, this approach not only provides conformational information but also offers a deeper understanding of the extent to which common solvation models in electronic structure methods introduce errors in electronic energies and molecular properties. In this way, we provide not only information about the most stable conformer, but an unparalleled level of detail and accuracy in conformational analysis in the *altro*‐series and of monosaccharides in general.

## 2 Results and Discussion

### 2.1 Computational Protocol

We start by outlining the results of our computational protocol that allows to obtain an overview of all likely conformations of a given structure. For each altrose derivative **1**, **2,** and **3** (Figure 1a), a hierarchical conformational search of the puckering space represented by the Cremer and Pople sphere in Figure 1b was carried out. The scheme applied here is based on a combination of conformational search tools and hierarchical energy refinement, inspired by the work of Mayes and coworkers^[^ ^4^ ^]^ refined and extended based on the results of our previous work.^[^ ^34^ ^]^

In a first step, a conformational search at the xTB2 level of theory^[^ ^50^, ^51^, ^52^, ^53^, ^54^, ^55^ ^]^ is carried out using the GOAT (global geometry optimization and ensemble generator) algorithm^[^ ^47^, ^48^ ^]^ as implemented in ORCA 6.0 (“GOAT entropy variant”). GOAT is a global optimizer that was developed by de Souza et al.^[^ ^56^ ^]^ based on Wales’ and Doye's basin‐hopping approach,^[^ ^57^ ^]^ Goedecker's minima hopping scheme,^[^ ^58^ ^]^ and ideas from Simulated Annealing and Taboo Search techniques. For all xTB structures in the ensemble generated by GOAT, the puckering amplitude Q and the puckering angles θ (0° > θ > 180°) and Φ (0° > Φ > 360°) are computed as defined by Cremer and Pople.^[^ ^49^ ^]^ Next, the ranges of θ and Φ were divided into bins of 30° for Φ and 20° for Φ (except for 140°‐180° and 40°‐0°, which are each one bin for ^1^ *C*_4_ and ^4^ *C*_1_, respectively). For each bin, a selection was made in which identical structures were eliminated, and up to 10 of the lowest energy structures in each bin were selected. Note that for some examples, no selection was carried out in this step, and all structures were carried on to the next step. The selected structures were then optimized at the M062X‐D3/def2‐SVP + CPCM level of theory.^[^ ^59^, ^60^, ^61^, ^62^ ^]^ Typically, if the initial ensemble is smaller than 1000 conformers, no selection is required for the first step. The resulting structures were analyzed and binned again, and the two or three lowest energy structures of each bin were selected, depending on how many local minima of sufficiently different puckering coordinates were present in a bin. For the selected ensemble, B3LYP‐D4/def2‐TZVP + CPCM optimization^[^ ^53^, ^63^, ^64^, ^65^, ^66^ ^]^ and frequency calculations were carried out, so that relative energies and free energies were obtained for this ensemble.

For each of the puckering conformers, the energy of the lowest energy structure was then evaluated at the DLPNO‐CCSD(T1)/cc‐pVTZ + CPCM level of theory.^[^ ^67^, ^68^, ^69^, ^70^, ^71^, ^72^, ^73^, ^74^, ^75^, ^76^, ^77^ ^]^ Note that for different solvents, all steps that include CPCM were repeated.

This way, a robust and unbiased assessment of the conformational landscape can be obtained, including implicit solvation. The final relative free energies of the different conformers were obtained by combining the DLPNO‐CCSD(T1) energies and the free energy corrections obtained at the B3LYP‐D4/def2‐TZVP level of theory. This resembles the original computational scheme proposed by Mayes et al. that has successfully been applied to a variety of carbohydrates.^[^ ^4^ ^]^

For the final ensemble, spin–spin coupling constants were evaluated at the PBE/pcJ‐3 + CPCM level of theory.^[^ ^77^, ^78^, ^79^, ^80^, ^81^, ^82^ ^]^ Furthermore, dipole moments are computed at the B3LYP/def2‐TZVP and MP2/aug‐cc‐pVTZ^[^ ^83^, ^84^, ^85^ ^]^ level of theory for comparison with the gas‐phase rotational spectroscopy data (vide infra). For more information on the computational details, see the Supporting Information (Section 1).

**Figure 2:**
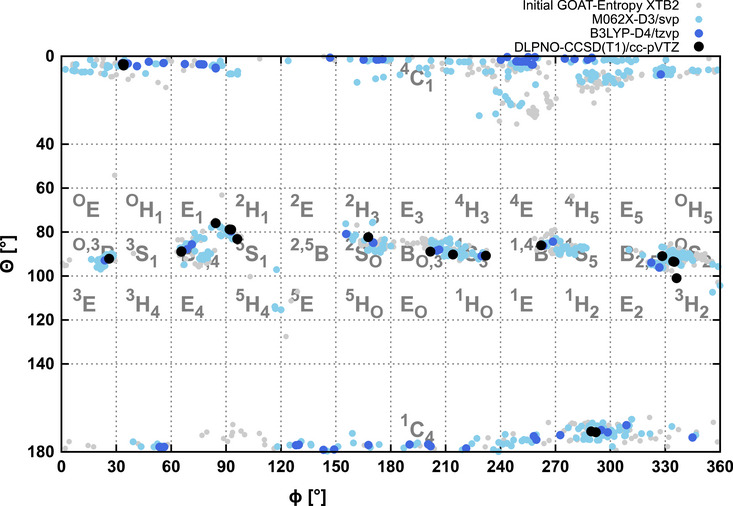
Hierarchical conformational search (see text for details) for the methyl altroside **1** (here without implicit solvent). From the initial GOAT entropy search, 504 structures are obtained and refined at the M06‐2X‐D3/def2‐SVP level of theory, 86 of these at the B3LYP‐D4/def2‐TZVP level of theory, which yields 44 final structures for which DLPNO‐CCSD(T1)/cc‐pVTZ energies are calculated. The nomenclature used to indicate the different classes of conformers and puckering angles is introduced in Figure 1b.

Figure 2 shows the results of the hierarchical conformer search for methyl altropyranoside **1**. The Mercator plot nicely illustrates the refinement of the few lowest energy structures. It also becomes obvious that there is quite a change of conformer structures going from xTB to DFT, and that while some areas of the mercator plot are populated, other possible conformers are missing from the map for altroside **1**. This way, out of 39 possible puckering conformers, only some 15 were actually found.

**Figure 3:**
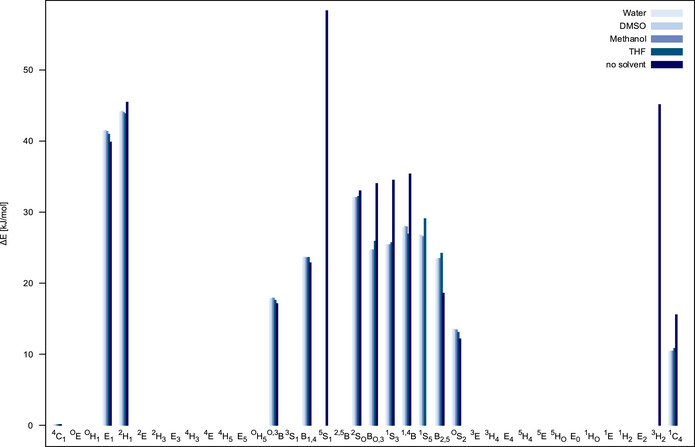
Relative free energies for methyl altroside **1** for the final structures of the hierarchical conformational analysis computed using DLPNO‐CCSD(T1)/cc‐pVTZ energies + B3LYP‐D4/def2‐TZVP free energy corrections, with and without CPCM (THF, CH_3_OH, DMSO, and H_2_O).

For the methyl altropyranoside **1**, the final ensemble was obtained for four different solvents (THF ε≈8, methanol ε≈33, DMSO ε≈47, and water ε≈80) and without CPCM. The final relative energies of the conformers obtained as local minima are shown in Figure 3. Here, it can be seen that the ^4^ *C*_1_ structure is the global minimum in every case, and no other structure is within 10 kJ/mol of this minimum. Solvation seems to generally stabilize other conformers, which is not surprising given the polar side chains of the structure. And while there are two high‐energy conformers that are only stable in very polar solvents, the differences between different solvents, as recovered by CPCM, are not very pronounced.

**Figure 4:**
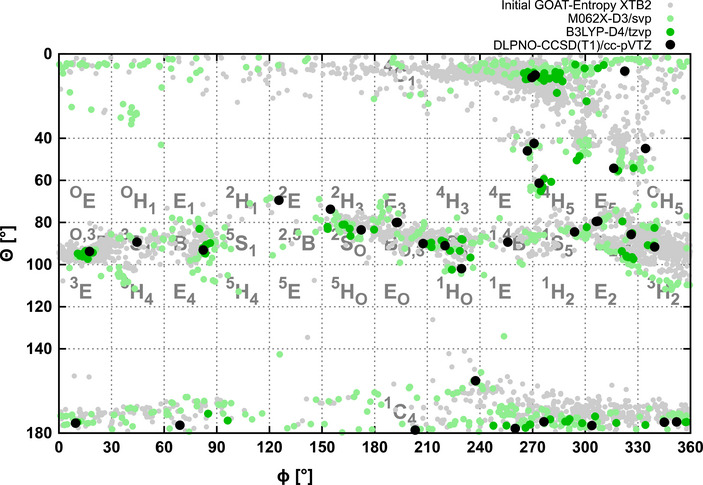
Hierarchical conformational search (see text for details) for the α‐altropyranosyl trichloroacetimidate **2** using CPCM (CDCl_3_). From an initial GOAT entropy search yielding 4702 structures, 722 are refined at the M06‐2X‐D3/def2‐SVP level of theory, 144 of these at the B3LYP‐D4/def2‐TZVP level of theory, which yields 35 final structures for which DLPNO‐CCSD(T1)/cc‐pVTZ energies are calculated.

**Figure 5:**
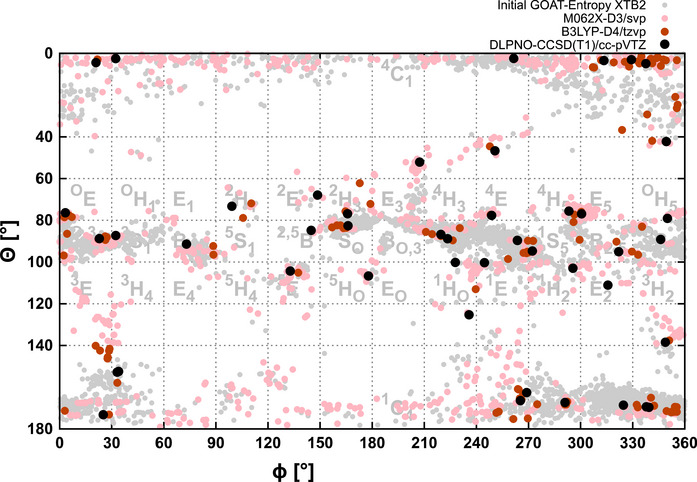
Hierarchical conformational search (see text for details) for the β‐altropyranosyl trichloroacetimidate **3** using CPCM (CDCl_3_). From an initial GOAT entropy search yielding 5317 structures, 1012 are refined at the M06‐2X‐D3/def2‐svp level of theory, 176 of these at the B3LYP‐D4/def2‐tzvp level of theory, which yields 47 final structures for which DLPNO‐CCSD(T1)/cc‐pVTZ energies are calculated.

The same analysis is shown in Figures 4 and 5 for the altropyranosyl trichloroacetimidates **2** and **3** without CPCM and with CPCM (for CDCl_3_, which is the solvent used in the NMR experiments). Focusing on the xTB results shown in light gray, it is clearly visible that the initial conformational search yields thousands of possible minima given the sizable flexible side chains in these systems. Already at the xTB level, the plots exhibit distinct differences between the two structures, like for the regions of 210° > Φ > 270° and 80° > θ > 100° or the region of 300° > Φ > 360° and 140° > θ > 180°. The successive refinement of the lowest energy structures at the M06‐2X and B3LYP level of theory shows that while the xTB already yields results of remarkable quality, the refinement shifts the distribution of conformers noticeably.

**Figure 6:**
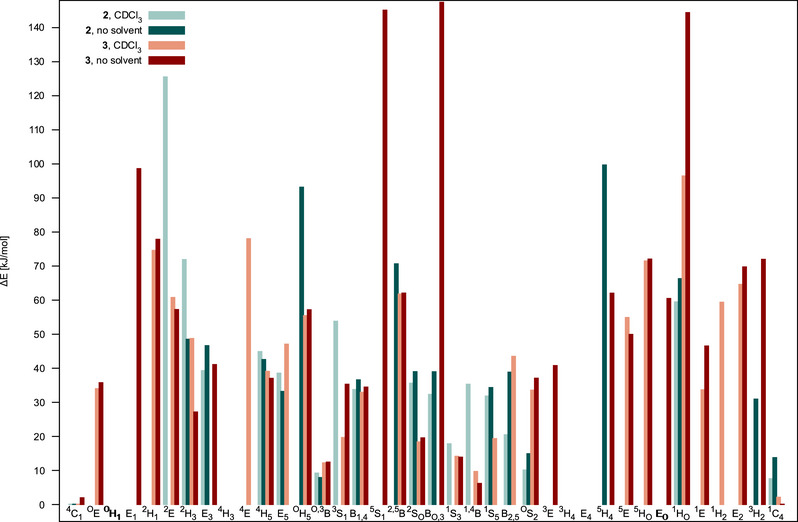
Relative free energies for **2** and **3** for the final structures of the hierarchical conformational analysis computed using DLPNO‐CCSD(T1)/cc‐pVTZ energies + B3LYP‐D4/def2‐TZVP free energy corrections, with and without CPCM (CDCl_3_).

Generally, a clustering of results and a coalescence of several initial structures due to a deepening of local minima at higher levels was observed (see the Supporting Information for more details). From Figure 6, it can be seen that for the α‐altropyranosyl trichloroacetimidate **2** and for its β‐anomer **3,** only very few conformers lie within a range of 10 kJ/mol. For **2,** these are the ^1^ *C*_4_ and ^4^ *C*_1_ conformers as well as the ^O,3^ *B*, and ^O^ *S_2_* conformers with a clear ^4^ *C*_1_ global minimum. For **3,** the situation is different – ^1^ *C*_4_ and ^4^ *C*_1_ are very close in energy, and if CDCl_3_ as an implicit solvent is used, the global minimum switches from ^1^ *C*_4_ to ^4^ *C*_1_. Besides these two, only the ^1,4^ *B* conformer is within 10 kJ/mol, while the ^O,3^ *B* conformer is slightly above. Upon closer inspection, further differences become obvious: There are several high‐energy conformers that are only present for **2**, like the ^O^ *E*, *E*_1_, ^2^ *H*_1_, ^4^ *E*, ^5^ *S*_1_, ^3^ *H*_4_, ^5^ *E*, ^5^ *H*_O_, ^1^ *E*, ^1^ *H*_2,_ and *E*_2_ conformers, for example. Another interesting aspect is the stabilization or destabilization of different conformers by solvation. While the relative energies of some conformers are hardly affected, the ^O^ *H*_5_ conformer of **2** and the ^2^ *H*_1_ conformer of **3** are only found without implicit solvent, while the ^1^S_3_ conformer of **2** and the *B_2,5_* conformer of **3** are only obtained upon inclusion of CDCl_3_.

### 2.2 Conformational Analysis in Solution via ^1^H NMR

For the conformational analysis, we used vicinal proton–proton coupling constants obtained from ^1^H NMR spectra of **1** (recorded in THF‐d_8_, methanol‐d_4_, DMSO‐d_6_, and D_2_O) and of **2** and **3** (recorded in neutralized CDCl_3_, Figure 1a). Following the scheme proposed in our last report ^[^ ^34^ ^]^ the spin–spin coupling constants of the final ensemble are computed at the PBE/pc‐J‐3 + CPCM level of theory. Then, the ^3^ *J*_H,H_ coupling constants of the pyranose ring are fitted to the experimental values with the weight of each conformer as the degrees of freedom for the least‐squares (LSQ) fit. In all cases, very similar RMSD values are obtained if the conformers fitted are reduced to the three or four most important contributions. The final results are shown in Table 1. Note that there are several sources of error for the corresponding fits: The numerics of the LSQ fit and artefacts due to overfitting, the computed structures used to calculate couplings, and the accuracy of the computed couplings themselves. While we have previously shown that the couplings computed at the PBE0/pc‐J3 + CPCM level of theory are accurate to about 1 Hz and the RMSD values given indicate a fit accuracy of better than 0.5 Hz, the percentages of the conformer mixture determined in the fit are much more sensitive (estimated errors lie in the range of ± 5%, for a detailed discussion, see Supporting Information).^[^ ^34^ ^]^ Thus, these values should rather be understood as indicators for major and minor components than as quantitative analysis.

**Table 1:** Experimental and theoretical coupling constants of the altrose derivatives **1**, **2**, and **3**. The ^3^ *J*_H,H_ coupling constants (in Hz) of the respective sugars were measured in different solvents, and theoretical coupling constants were calculated at the PBE/pc‐J3^[^ ^77^, ^78^, ^79^, ^80^, ^81^, ^82^ ^]^ level of theory with implicit solvent. Experimental values were obtained from apodised ^1^H NMR spectra (cf. Supporting Information, Section 4). Conformational ratios were obtained by fitting calculated and experimental values, and root‐mean‐square deviations (RMSD) are given, error estimates are ± 1 Hz for computed couplings and ± 5% for the fitted compositions.

Altroside	Solvent	*J*_1,2_	*J*_2,3_	*J*_3,4_	*J*_4,5_	RMSD	Conformer ratio
**1 (obs)**	D_2_O	1.9	4.3	3.5	8.9		
**1 (calc ^4^ *C*_1_)**	D_2_O	1.4	3.7	4.0	10.3		
**1 (calc ^1^ *C*_4_)**	D_2_O	7.7	9.7	3.9	1.0		
**1 (calc ^O,3^B)**	D_2_O	0.1	6.2	3.7	0.3		
**1 fit**	D_2_O	1.9	4.3	3.9	8.9	0.22	^4^ *C*_1_: ^O,3^B: ^1^ *C*_4_ 86: 6: 8
**1 (obs)**	methanol‐d_4_	1.6	3.9	3.3	9.5		
**1 (calc ^4^ *C*_1_)**	methanol‐d_4_	1.5	3.6	4.0	10.2		
**1(calc ^O,3^B)**	methanol‐d_4_	0.1	6.1	3.7	0.3		
**1(calc B_O,3_)**	methanol‐d_4_	6.4	1.2	2.7	8.2		
**1 fit**	methanol‐d_4_	1.6	3.7	3.9	9.4	0.31	^4^ *C*_1_: ^O,3^B: B_O,3_ 88: 7: 5
**1 (obs)**	DMSO‐d_6_	1.9	4.2	3.5	8.9		
**1 (calc ^4^ *C*_1_)**	DMSO‐d_6_	1.5	3.7	4.0	10.3		
**1 (calc ^1^ *C*_4_)**	DMSO‐d_6_	7.7	9.7	3.9	1.0		
**1 (calc B_O,3_)**	DMSO‐d_6_	6.4	1.2	2.7	8.2		
**1 (calc ^O,3^B)**	DMSO‐d_6_	0.1	6.1	3.7	0.3		
**1 fit**	DMSO‐d_6_	1.9	4.1	3.9	8.9	0.21	^4^ *C*_1_: B_O,3_: ^O,3^B: ^1^ *C*_4_ 82: 4: 8: 6
**1 (obs)**	THF‐d_8_	1.7	3.7	n.d.	n.d.		
**1 (calc ^4^ *C*_1_)**	THF‐d_8_	1.6	3.7	4.0	10.2		
**2 (obs)**	CDCl_3_	1.0	3.4	3.3	10.2		
**2 (calc ^4^ *C*_1_)**	CDCl_3_	1.2	3.4	3.5	10.6		
**2 (calc E_3_)**	CDCl_3_	1.5	3.5	3.0	8.5		
**2 fit**	CDCl_3_	1.2	3.4	3.4	10.2	0.13	^4^ *C*_1_: E_3_ 80: 20
**3 (obs)**	CDCl_3_	2.8	8.1	3.3	5.0		
**3 (calc ^4^ *C*_1_)**	CDCl_3_	1.7	4.0	3.7	10.7		
**3 (calc ^1^ *C*_4_)**	CDCl_3_	4.2	11.7	3.8	1.0		
**3 (calc ^3^S_1_)**	CDCl_3_	3.7	5.4	3.8	0.2		
**3 fit**	CDCl_3_	3.1	8.1	3.7	5.1	0.28	^4^ *C*_1_: ^3^S_1_: ^1^ *C*_4_ 42: 7: 51

For the methyl altroside **1**, the ^4^ *C*_1_ conformer dominates in all solvents besides small ratios of ^O,3^ *B*, and *B*_O,3_, as well as minor contributions of ^1^ *C*_4_. In D_2_O, the second most populated species is the ^1^ *C*_4_ conformation (8%), while in methanol‐d_4_ and DMSO‐d_6_ the second most populated conformer is ^O,3^ *B*. The larger share of boat conformations in comparison to ^1^ *C*_4_ might be caused by the high‐energy conformers between 80° > θ > 0° (Figure 2), which makes puckering between the equator and ^1^ *C*_4_ less likely. As we could not determine reliable coupling constants of **1** in THF‐d_8_, we have not performed a least‐squares fit, but already by simply comparing the calculated coupling constants of ^4^ *C*_1_ with the measured ones, it is evident that ^4^ *C*_1_ is the main conformation here too.

For the α‐altropyranosyl trichloroacetimidate **2**, the ^4^ *C*_1_ conformer dominates with a proportion of 80% followed by 20% of the *E*_3_ conformer. This is surprising, since envelopes are usually considered as transition states in ring inversion itineraries, but since normal mode analysis confirms *E*_3_ as the minimum structure adjacent to ^4^ *C*_1_, a transition between these conformers is conceivable. An additional transition state search for a ^4^ *C*_1_ to *E*_3_ conversion indeed corroborates this assumption, for details, see Supporting Information.

The β‐d‐altropyranosyl trichloroacetimidate **3** shows different conformational properties in comparison to the α‐anomer **2**. As already indicated by the relative free energies shown in Figure 6, the similar energies of the complementary chair conformations are also reflected in the obtained fit, which yields a conformational population of 5:4:1 of 51% ^1^ *C*_4_, 42% ^4^ *C*_1,_ and 7% ^3^ *S*_1_. The larger proportion of ^1^ *C*_4_ might be due to the anomeric effect that stabilizes the axial orientation of the anomeric bond, thus, the equatorially oriented ^4^ *C*_1_ conformation becomes less likely.^[^ ^86^ ^]^

The results of the fit show a high degree of consistency with the computed relative energies for all investigated altrose derivatives. The correct minima are identified, and for altrosyl trichloroacetimidate **3,** the fit yields the expected mixture of two major components that are close in relative energy. We even find perfect correspondence for the three lowest energy conformers of **3** and all relevant contributions to the fit. However, in the fit for **1** and **2**, minor components occur that have very high relative energies, like the ^O,3^ *B* conformation of **1**.

Energy discrepancies like this, which frequently occur when computed data using approximated solvation models are compared with experiment, can be disentangled by conducting conformational studies in the gas phase. Here, molecules are investigated in isolation, free from medium‐induced structural influences. In this respect, rotational spectroscopy combined with a supersonic jet provides an ideal setting for such studies, as molecules in the gas phase are probed in a collision‐free environment. Additionally, the high resolution of the technique allows for precise structural determination, encompassing not only ring puckering but also the arrangement and the orientation of the side chains as well as the hydrogen bond networks that stabilize the conformers. Thus, rotational spectroscopy provides information on the preferred global conformations of a carbohydrate. Consequently, our discussion of conformers that has thus far primarily centered on variations in ring puckering has to be expanded by conformers in the orientation of their side chains when rotational spectroscopy is employed.^[^ ^37^, ^38^, ^39^, ^40^, ^41^, ^42^, ^43^ ^]^ Here, to establish a conclusive benchmark and bridge the gap between theory and experiment, we used rotational spectroscopy to investigate the conformational landscape of methyl α‐d‐altropyranoside (**1**).

### 2.3 Conformational Analysis of Altroside 1 by Rotational Spectroscopy and X‐ray Crystallography

**Figure 7:**
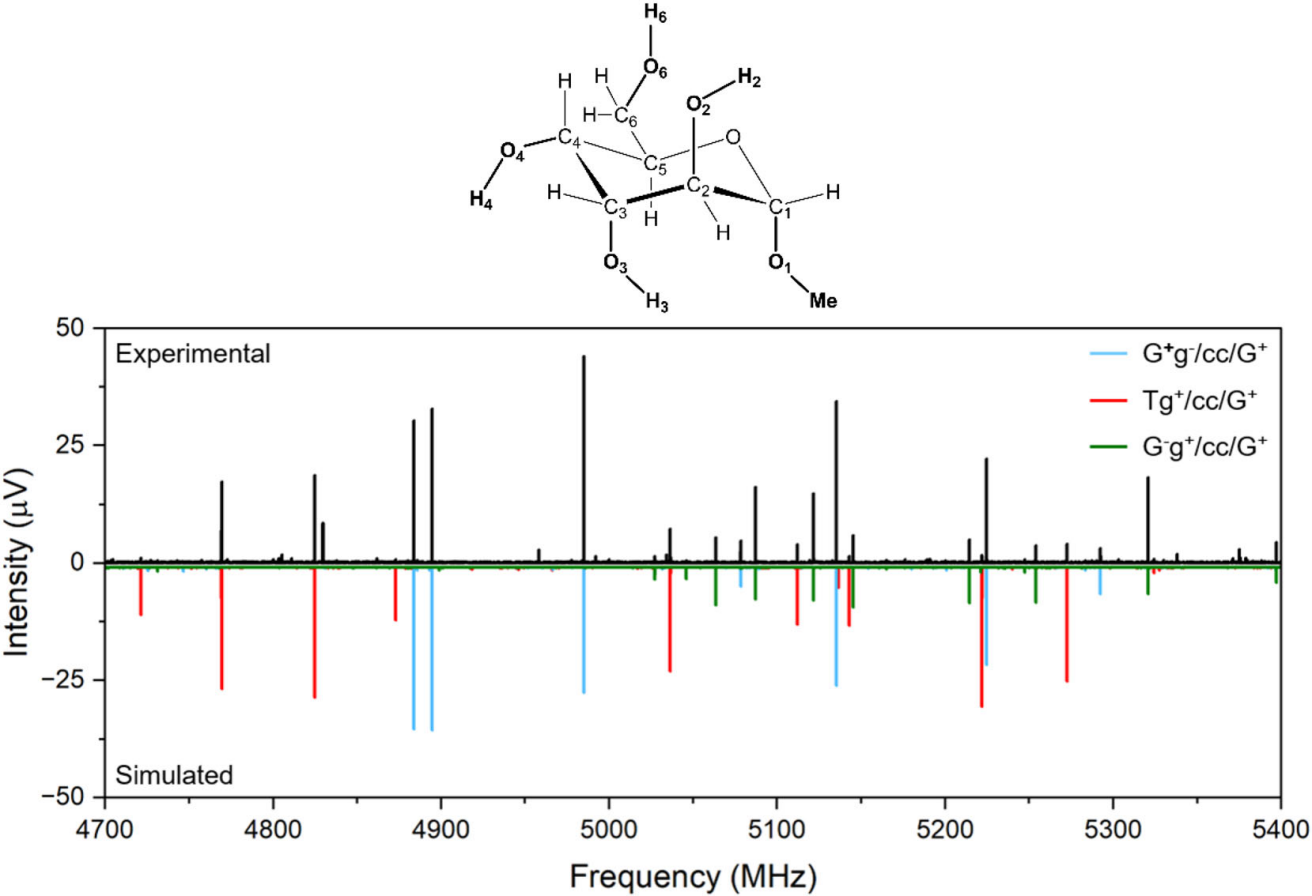
A section of the broadband rotational spectrum of methyl α‐d‐altropyranoside recorded in the 2–8 GHz frequency range. The upper trace (black) shows the experimental rotational spectrum. The lower trace (colored and inverted, for clarity) shows the simulated rotational spectra of the three assigned conformers. The simulations were performed using the experimentally determined rotational constants and a rotational temperature of 1 K.

To further explore the conformational properties of methyl altroside **1** in the gas and solid phases, we performed rotational spectroscopy and analyzed single crystals, as described in this subsection. In order to analyze the microwave spectrum of **1**, we focused on the conformers falling within an energy window of 10 kJ/mol. Conformers above this energy threshold are unlikely to be significantly populated in the supersonic jet and, thus, their rotational transitions are not expected to appear in the rotational spectrum. In total, we identified nine low‐lying energy conformers below 10 kJ/mol as potential candidates in the spectrum. They all share a ^4^ *C*_1_ ring conformation and differ in the orientation of the hydroxymethyl group at C‐5 and of the hydroxyl group at C‐2 (2‐OH), as shown in Figure S15. To distinguish these conformers, we followed standard conventions in carbohydrate analysis by referring to the orientation of a certain atom (like oxygen in OH) to a vicinal reference atom. Therefore, we have adopted a three‐term labelling system similar to that used in previous gas‐phase studies of monosaccharides.^[^ ^39^ ^]^ In general, in this system, the first term, consisting of two letters, namely G^+^, G^−^, or T and g^+^, g^−^, or t, respectively, describes the overall orientation of either oxygen or hydrogen of a hydroxy group relative to a vicinal carbon or the endocyclic oxygen. Specifically, G^+^, G^−^, or T and g^+^, g^−^ or t correspond to dihedral angles of + 60°, ‐60° or 180° for the O_6 _− C_6 _− C_5 _− O_5_ and H_6 _− O_6 _− C_6 _− C_5_ angles, respectively, (Figure 7). The second term of the label describes the orientation of the cooperative hydrogen‐bond network, which can assume a clockwise (cl) or a counter clockwise (cc) direction. Typically, a third term is used to describe the orientation of the substituent at the anomeric carbon (C_1_). However, as no mutarotation occurs in methyl glycosides, we did not include this term. All nine conformers of **1** share a T orientation of the methoxy group. However, as already mentioned above, they differ in the orientation of the 2‐OH group, which can also adopt a G^+^, G^−^, or T conformation depending on whether the dihedral angle H_2 _− O_2 _− C_2 _− C_1_ is + 60°, ‐60° or 180°. Therefore, this third term is used here to define the orientation of the 2‐OH group. For all the conformers, rotational constants, dipole‐moment components as well as relative free energies were computed and reported in Table S1 of the Supporting Information.

The gas‐phase conformational space of methyl α‐d‐altropyranoside (**1**) was explored using the chirped‐pulse Fourier transform microwave spectrometer COMPACT, which operates in the 2–18 GHz frequency range. Experimental conditions are detailed in the Supporting Information (Section 2.1). A portion of the experimental rotational spectrum, which in the case of methyl α‐d‐altropyranoside (**1**) was recorded in the 2–8 GHz frequency range, is shown in Figure 7, where the analysis revealed three distinct spectra exhibiting characteristic patterns. Fitting these spectra yielded three unique sets of rotational constants. By comparing the measured rotational constants and types of observed rotational transitions with the calculated structures, their relative energies and predicted dipole‐moment components, the three lowest‐energy, nearly isoenergetic conformers of the methyl altroside **1**: G^+^g^−^/cc/G^+^, Tg^+^/cc/G^+^, and G‐g^+^/cc/G^+^, could be assigned from the rotationally resolved spectral features.

Notably, conformers sharing the same hydroxymethyl group orientation exhibit similar rotational constants, making it difficult to assign the individual observed spectra solely based on a comparison of theoretical and experimental rotational constants. However, changes in the orientation of the 2‐OH group result in differences in the magnitude and ratios of the dipole‐moment components, which proved crucial for the unambiguous assignment of the rotational spectrum. The three sets of rotational transitions, belonging to three different, individual conformers, were each fitted using an asymmetric‐top Hamiltonian (cf. Supporting Information, Section 2). The experimental rotational constants for the three different conformers are summarized in Table 2, whereas the measured rotational transition frequencies are listed in the Supporting Information (Tables S2‐S4). In addition, some rotational transitions observed in the rotational spectra of all three assigned conformers exhibited characteristic fine structure due to the internal rotation of the methyl group of the O‐CH_3_ moiety, consistent with the theoretical values of the corresponding V_3_ barriers of these internal motions (Figure S16). However, the number of rotational transitions exhibiting such fine structure was insufficient to experimentally determine the height of the respective methyl internal rotation barriers.

**Table 2:** Experimental and theoretical (B3LYP‐D4/def2‐TZVP) spectroscopic constants of methy α‐d‐altropyranoside.

	G^+^g^−^/cc/G^+^		Tg^+^/cc/G^+^		G^−^g^+^/cc/G^+^	
	Theo.	Exp.	Theo.	Exp.	Theo.	Exp.
*A* (MHz)^a^	867.4	872.74810(28)	971.8	970.20007(41)	911.2	912.16263(33)
*B* (MHz)	755.3	760.14590(23)	684.2	693.13049(40)	715.0	720.79211(35)
*C* (MHz)	581.6	582.72933(26)	570.1	572.26102(41)	612.4	615.93365(40)
Δ*_J_* (kHz)^b^	‐	0.0547(37)	‐	0.0416(66)	‐	0.0173(68)
|*μ_a_* | (D)^c^	0.4	y^g^	2.6	y	2.8	y
|*μ_b_* | (D)	3.1	y	1.8	y	3.3	y
|*μ_c_* | (D)	0.3	n	0.1	n	1.1	y
ΔG (kJ/mol)^d^	0		0.3		0.4	
N^e^	‐	61	‐	70	‐	75
RMS (kHz)^f^	‐	7.0	‐	8.5	‐	7.9

^a^ *A*, *B*, and *C* are the rotational constants.

^b^ Δ*_J_* is the centrifugal distortion constant.

^c^ *μ_a_, μ_b_, and μ_c_* are the electric dipole‐moment components along the principal inertial axes.

^d^ Δ*G* are the relative Gibbs energies calculated at the B3LYP‐D4/def2‐TZVP level of theory.

^e^ Total number (N) of lines in the fit.

^f^ Root‐mean‐square deviation of the fit.

^g^ Yes (y) or no (n) observation of *a*‐, *b*‐, and *c*‐type transitions.

Conformational relative abundances were estimated by measuring the relative intensities of a selected set of *b*‐type transitions common among the three assigned conformers, while considering the magnitude of the computed dipole‐moment components. When splitting due to methyl internal rotation was present, the sum of the intensities of the two components was considered in order to account for it. This approach yielded conformational relative abundances of 59%, 29%, and 12% for the G^+^g^−^/cc/G^+^, Tg^+^/cc/G^+^, and G^−^g^+^/cc/G^+^, respectively.

**Figure 8:**
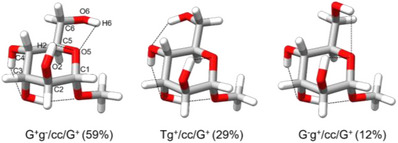
Optimized structures of the three observed conformers of methyl α‐d‐altropyranoside (**1**) calculated at the B3LYP‐D4/def2‐TZVP level of theory, showing their hydrogen bond networks. The atom numbering, as well as the relative conformational abundances in percentages, are also provided.

Figure 8 shows the structures of the three observed conformers, which differ solely in the orientation of the hydroxymethyl group. Each conformer is stabilized by a network of intramolecular O‐H···O hydrogen bonds. In the G^+^g^−^/cc/G^+^ and G^−^g^+^/cc/G^+^ conformers, this network consists of a hydrogen‐bond chain involving 4‐OH···3‐OH···1‐OMe. In the Tg^+^/cc/G^+^ conformer, the network is extended with the inclusion of 6‐OH, forming the sequence: 6‐OH···4‐OH···3‐OH···1‐OMe. Instead, in the G^+^g^−^/cc/G^+^ and G^−^g^+^/cc/G^+^ conformers, the 6‐OH group participates in a bifurcated hydrogen bond with both the endocyclic oxygen atom (O_5_) and the 2‐OH hydroxy group. In the Tg^+^/cc/G^+^ conformer, an isolated hydrogen‐bond interaction occurs between the 2‐OH group and the O_5_ atom.

Consistent with recent studies on methyl glycosides, such as methyl α,β‐d‐glucopyranoside and methyl α,β‐d‐galactopyranoside,^[^ ^43^ ^]^ all observed conformers of methyl α‐d‐altropyranoside (**1**) exhibit a counter‐clockwise hydrogen‐bond network. This preference for a counter‐clockwise orientation in methyl glycosides is attributed to the influence of the methyl aglycone.^[^ ^43^ ^]^ Indeed, while the anomeric hydroxy group (1‐OH) in reducing sugars can act as both a hydrogen bond donor and acceptor, the 1‐OMe group in methyl glycosides functions only as a H‐bond donor, making a clockwise hydrogen‐bond network energetically unfavorable.

In the literature, the G^+^ conformation of the hydroxymethyl group is often described as the most abundant conformer, followed by the G^−^ conformation, while the T conformation is generally regarded as the least abundant. This trend has been observed across most monosaccharides studied to date.^[^ ^39^, ^40^, ^43^, ^87^ ^]^ The preference for the gauche conformations, G^+^ and G^−^, is often explained in terms of the gauche effect,^[^ ^43^, ^88^ ^]^ which suggests that electronic interactions, such as hydrogenbonding and hyperconjugation, can stabilize the gauche conformations despite the presence of steric hindrance. However, in the case of methyl α‐d‐altropyranoside (**1**), the gauche effect cannot explain the observed conformational abundances, as the T conformation prevails over the G^−^ conformation. A similar trend has been reported recently for methyl α,β‐d‐galactopyranoside.^[^ ^43^ ^]^

Note that the crystallographic analysis of **1** is in agreement with the findings of the microwave investigation (for details, see the Supporting Information). Crystal structure studies of methyl α‐d‐altropyranoside (**1**)^[^ ^89^ ^]^ provided positions of all atoms, including the hydrogens. Interestingly, the X‐ray analysis of the crystallized form did not show the T conformation but revealed only the presence of the G^+^ and G^−^ conformations. A comparison of the structures of methyl α‐d‐altropyranoside (**1**) in the gas phase and in the crystal revealed that, while the positions of the heavy atoms remain unchanged, the orientations of the hydrogen atoms change to favor the formation of intramolecular hydrogen bonds (see Figure 8). This finding is also in line with the earlier crystallographic study by Gatehouse and Poppleton, who also observed the ^4^ *C*_1_ conformation of the ring but reported exclusively the G^+^ conformer in the solid state.^[^ ^90^ ^]^

To summarize, in our quest to rationalise and predict the conformation of sugars, we previously demonstrated that a combination of theory and NMR parameters can be used to assign complex conformational equilibria. However, we also found that this approach is limited by the ability of electronic structure methods to describe relative free energies in solution. The agreement of rotational spectroscopy and theory is close to perfect, and the observed conformers are accurately reproduced by the computed order in stability.

**Figure 9:**
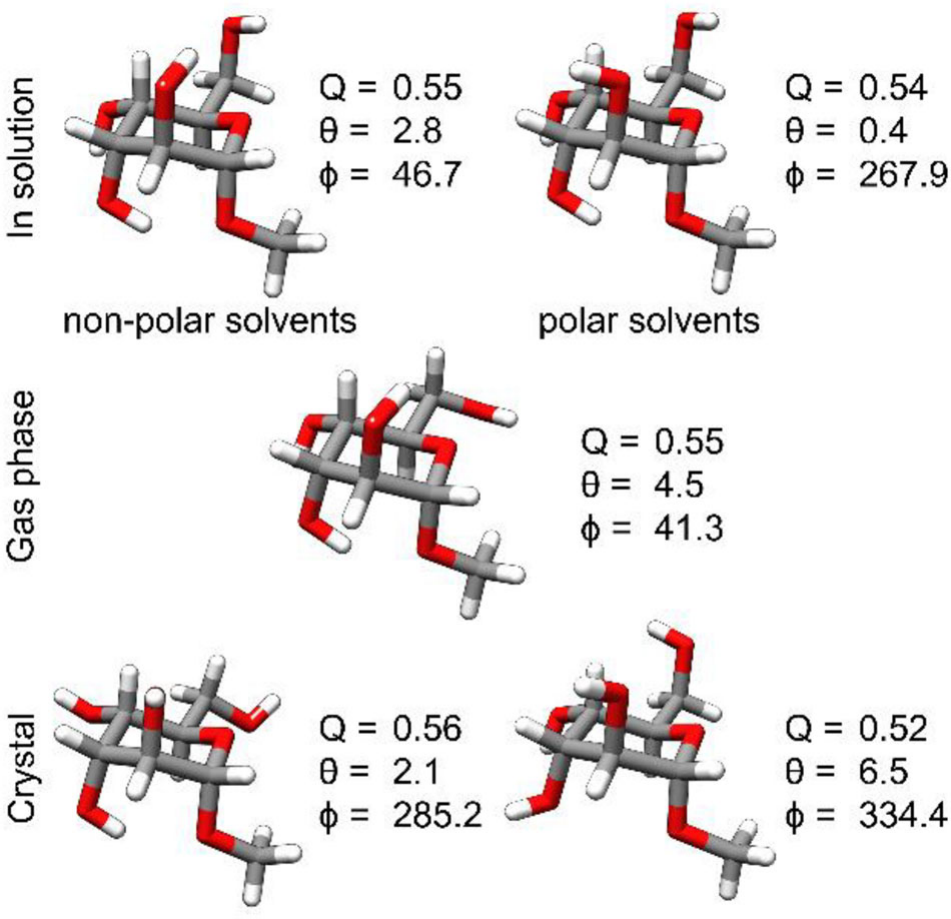
The structures of methyl α‐D‐altropyranoside (1) were determined in solution (polar and nonpolar solvents), in the gas phase, and in the crystal. The Cremer‐Pople parameters (Q, θ, and ϕ) indicate that the ^4^C_1_ chair is the most energetically favorable conformation in all phases.

Hence, rotational spectroscopy effectively bridges the ”solvent gap“ between theory and experiment by establishing a direct link to the computational structures. Consequently, the synergy of theory, NMR analysis, rotational spectroscopy, and crystallography enables us to comprehensively investigate the conformational properties of methyl α‐d‐altropyranoside (**1**) in solution, gas phase, and in the crystal. The structures obtained from the different experiments and simulations are displayed in Figure 9 along with the associated Cremer‐Pople parameters.

## 3 Conclusion

In this work, we systematically outline the complementarity and synergy of theory, NMR spectroscopy, and microwave spectroscopy in the structure elucidation of carbohydrates. By integrating the advantages of the three methods, we achieved an exhaustive investigation across all conformational degrees of freedom. A new computational workflow, including global optimization tools and a hierarchy of electronic structure methods, provides a comprehensive view of both local and global conformations. NMR spectroscopy offers insights into the preferred puckering conformation in solution, and microwave spectroscopy reveals the preferred global conformations in a solvent‐free environment.

The use of cross‐phase spectroscopic techniques in both solution and gas phase is particularly valuable in disentangling that it is the lack of accurate solvent models that makes direct comparison between theory and structure in solution challenging, even when high‐level ab initio methods are used. Indeed, while we have shown that the computed structures using implicit solvent models are not in perfect agreement with the NMR results in solution, the agreement between the microwave data and theory is close to perfect, with the relative stability being well represented by the experimentally observed conformers. These results highlight the benefit of combining theory with a cross‐phase spectroscopic approach to disentangle complex conformational equilibria and may inspire the development of more sophisticated computational methods to improve the description of the effects of solvation. This way, experimental results can be used to guide the refinement of computational methods for the description of carbohydrate building on the synergy between theory and experiment in the spirit of similar successful synergy projects that explore the potential of combining computational methods and spectroscopic techniques.^[^ ^91^, ^92^, ^93^ ^]^

Note that it might be advantageous to consider expanding the NMR analysis to include heteronuclear coupling constants in future work. The Serianni group has demonstrated that these couplings can provide valuable additional information on glycosidic linkages, side‐chain orientations, and ring puckering ^[^ ^94^, ^95^, ^96^, ^97^, ^98^, ^99^ ^]^ and they may also prove useful in our analytical approach.

The computational workflow outlined in this work can potentially be extended to any carbohydrate to rationalize the conformational degrees of freedom and foster the knowledge on conformation‐function relationships in the glycosciences.

## Supporting Information

Supporting Information is available. The authors have cited additional references within the Supporting Information.^[^ ^100^, ^101^, ^102^, ^103^, ^104^ ^]^

## Conflicts of Interest

The authors declare no conflict of interest

## Supporting information



Supporting Information

Supporting Information

## Data Availability

The data that support the findings of this study are available in the supplementary material of this article.
