# Supplementary material for: Synergy of Theory, NMR, and Rotational Spectroscopy to Unravel Structural Details of d‐Altroside Puckering and Side Chain Orientation
Source: Chemistry. 2025 Nov 10;31(71):e02358. doi: 10.1002/chem.202502358 (PMC12734650; doi:10.1002/chem.202502358)
Supplement: Supplementary file 1 — Supporting Information [file CHEM-31-e02358-s001.docx]

**Supporting Information**

**Synergy of Theory, NMR and Rotational Spectroscopy to Unravel Structural Details of d-Altroside Puckering and Side Chain Orientation**

Donatella Loru, Clemens Lütjohann, Christian Näther, Thisbe K. Lindhorst, Melanie Schnell*, Alexander A. Auer*

**Table of Contents**

[1. Computational details 2](#_Toc208400680)

[1.1 Methyl α-d-altropyranoside (**1**) 2](#_Toc208400681)

[1.2 *O*-(2,3,4,6-Tetra-*O*-acetyl-α-d-altropyranosyl) trichloroacetimidate (**2**) 6](#_Toc208400682)

[1.3 *O*-(2,3,4,6-Tetra-*O*-acetyl-β-d-altropyranosyl) trichloroacetimidate (**3**) 11](#_Toc208400683)

[1.4 Example - transition state search for structures connecting different minima 16](#_Toc208400684)

[1.5 Least squares fit procedure and error estimate for conformational equilibria 18](#_Toc208400685)

[1.6 File attachment: Molecular structures and energies 24](#_Toc208400686)

[2. Microwave spectroscopy of methyl α-d-altropyranoside (**1**) 26](#_Toc208400687)

[2.1 Experimental method – Chirped pulse Fourier transform microwave spectroscopy 26](#_Toc208400688)

[2.2 Theoretical results 28](#_Toc208400689)

[2.2.1 Methyl internal rotation 29](#_Toc208400690)

[2.2.2 Conformational relaxation barriers 30](#_Toc208400691)

[2.2.3 Frequency list of the G^+^g^-^/cc/G^+^ conformer of methyl α-d-altropyranoside (**1**) 31](#_Toc208400692)

[2.2.4 Frequency list of the G^-^g^+^/cc/G^+^ conformer of methyl α-d-altropyranoside (**1**) 33](#_Toc208400693)

[2.2.5 Frequency list of the Tg^+^/cc/G^+^ conformer of methyl α-d-altropyranoside (**1**) 34](#_Toc208400694)

[3. Crystal structure of methyl α-d-altropyranoside (**1**) 37](#_Toc208400695)

[4. NMR studies 40](#_Toc208400696)

[4.1.1 Methyl α-d-altropyranoside (**1**) 40](#_Toc208400697)

[4.1.2 *O*-(2,3,4,6-Tetra-*O*-acetyl-α-d-altropyranosyl)trichloroacetimidate (**2**) 41](#_Toc208400698)

[4.1.3 *O*-(2,3,4,6-Tetra-*O*-acetyl-β-d-altropyranosyl)trichloroacetimidate (**3**) 42](#_Toc208400699)

[4.2 NMR spectra of methyl α-d-altropyranoside (**1**) 43](#_Toc208400700)

[4.3 NMR spectra of *O*-(2,3,4,6-Tetra-*O*-acetyl-d-altropyranosyl) trichloroacetimidates **2** and **3** 51](#_Toc208400701)

[5. Concluding remarks on the spectroscopical results in light of the anomeric effect 53](#_Toc208400702)

[6. References 54](#_Toc208400703)

# Computational details

## Methyl α-d-altropyranoside (1)

This subchapter presents Mercator plots of the conformer ensembles of methyl altroside **1**, obtained at different levels of theory. All calculations have been carried out using a development version of the ORCA 6.0 program package.^[1]^ Figure S1 illustrates the results after an initial conformational search via GOAT at the xTB level of theory.^[2]^ The first refinement of the obtained conformers was carried out at the M06-2X-D3/def2-svp level of theory for different solvents (using CPCM)^[3]^ and without solvation. The results are shown in Figure S2. Figure S3 displays the ensembles calculated at the B3LYP-D4/def2-tzvp level of theory,^[4]^ while Figure S4 summarizes the lowest energy structures for which DLPNO-CCSD(T1)/cc-pVTZ^[5]^ energies have been evaluated.


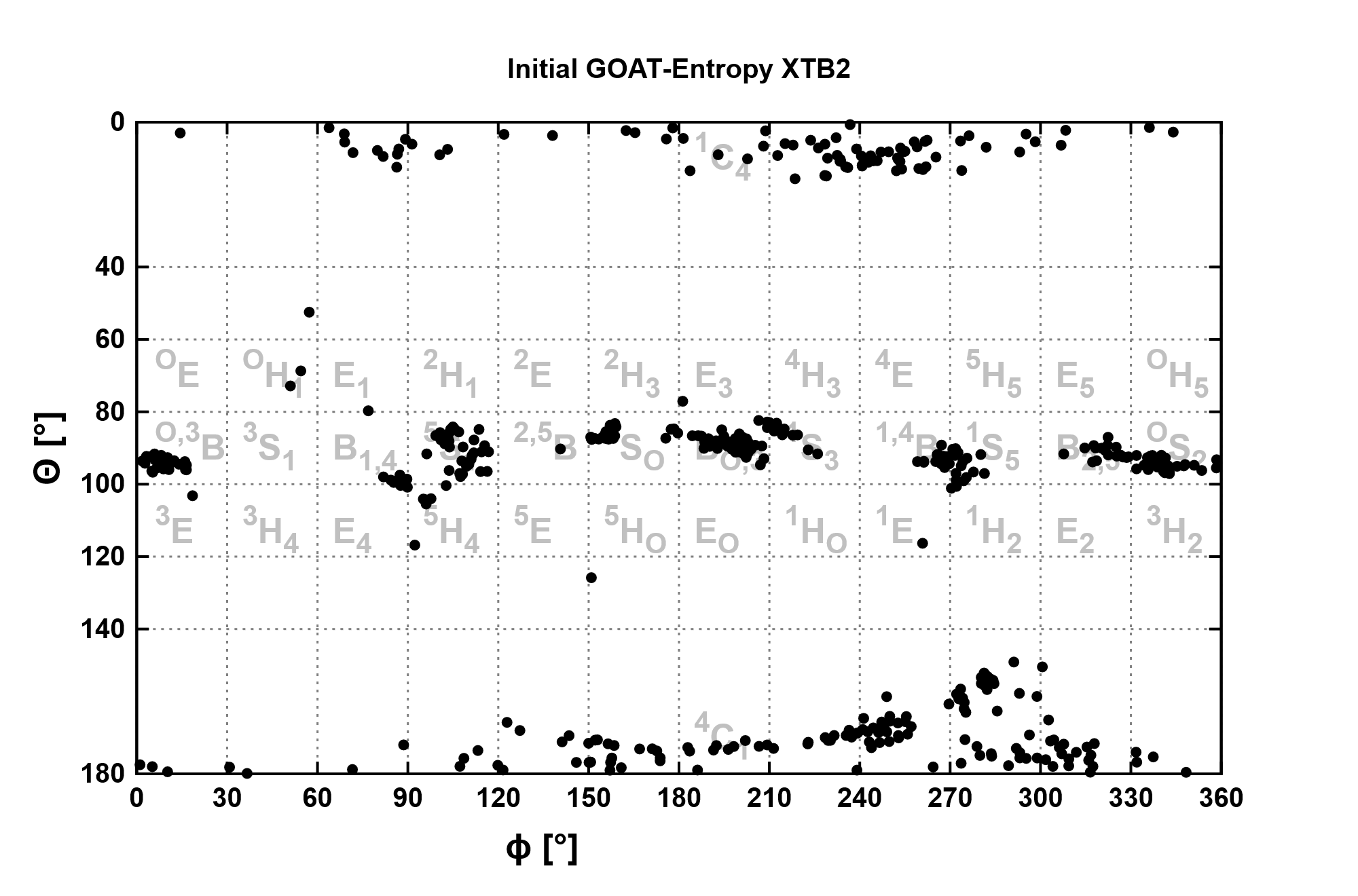
**Figure S1.** GOAT ensemble (504 structures) obtained at the xTB level of theory.


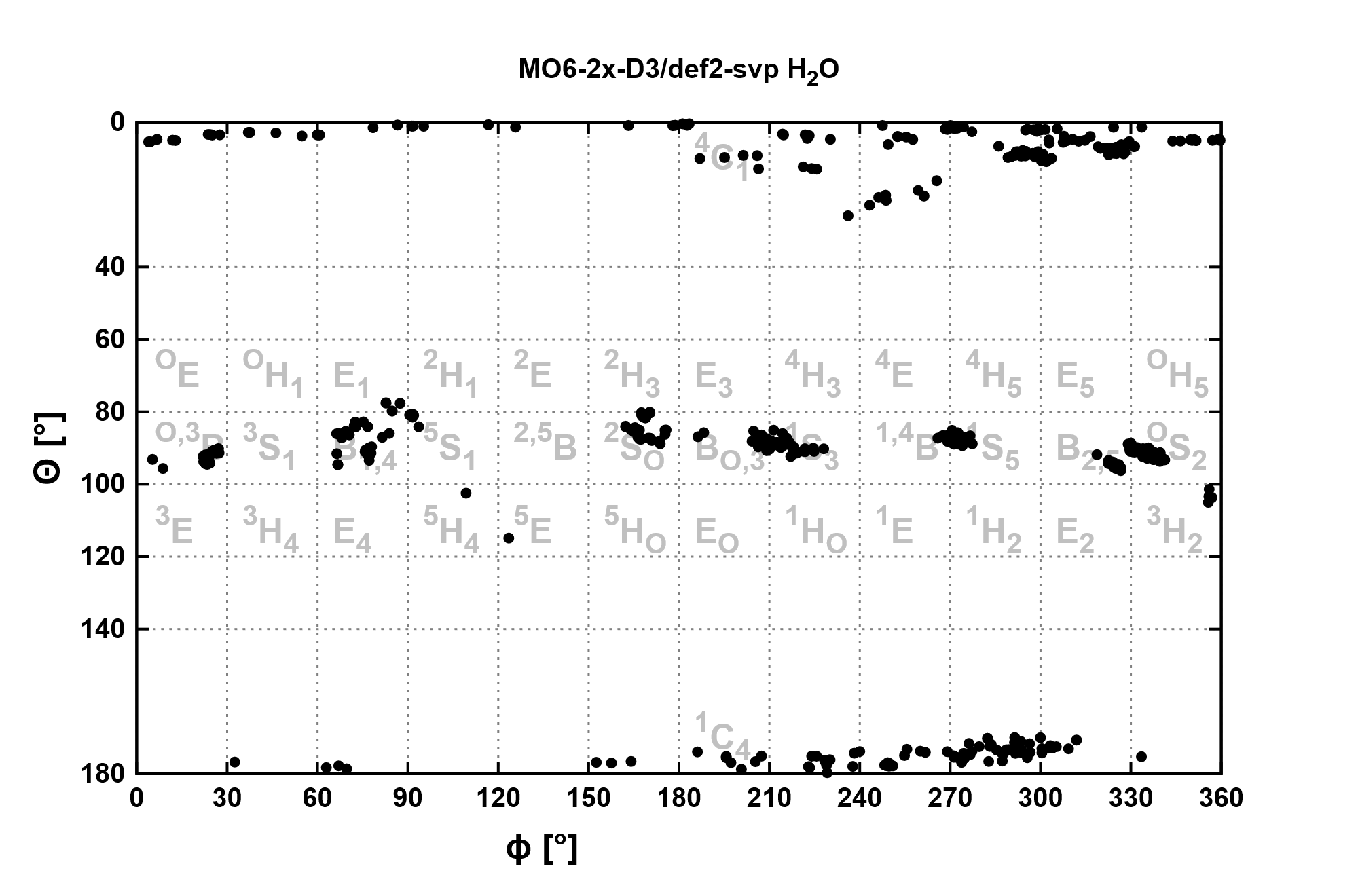

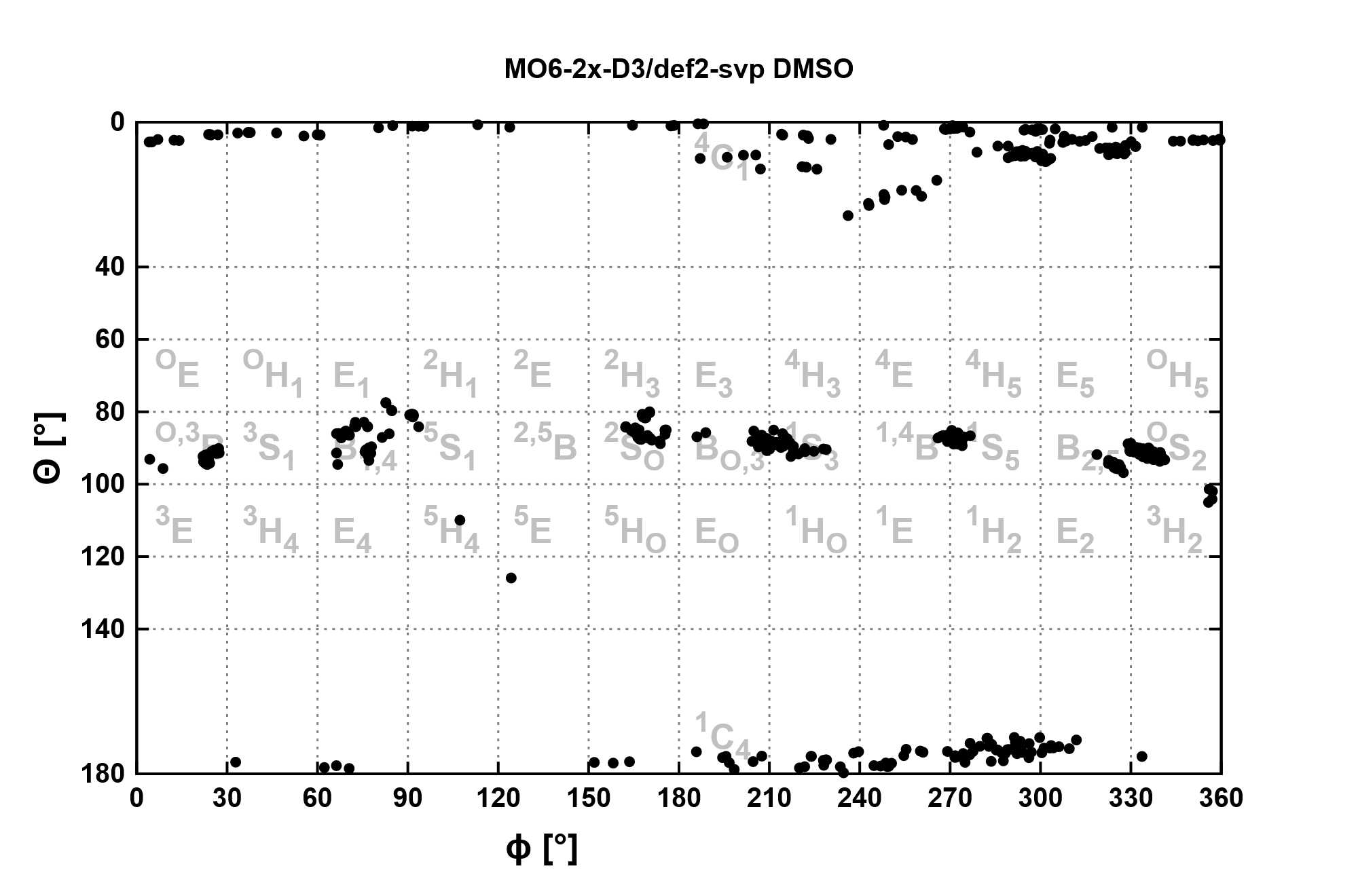

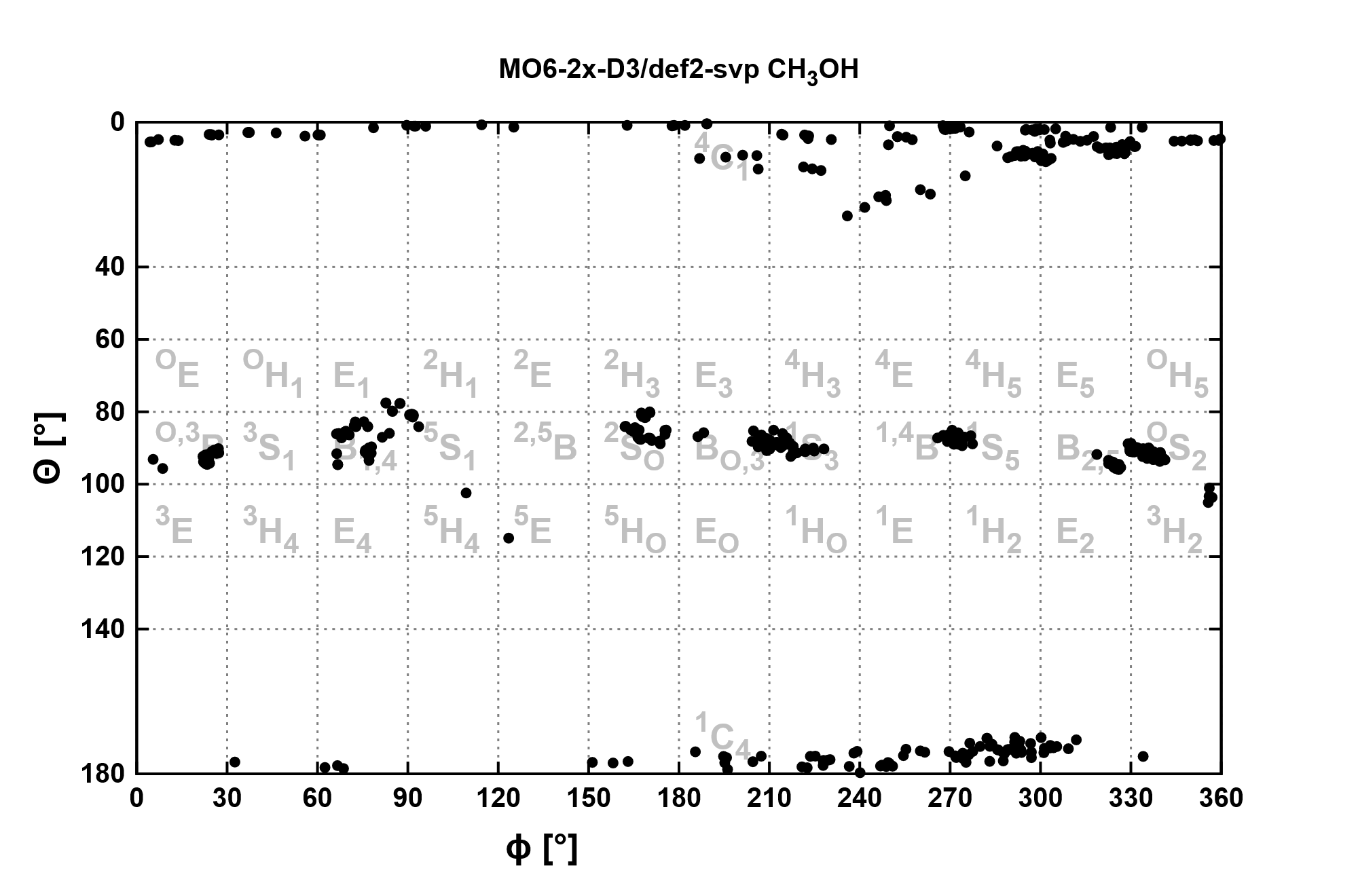

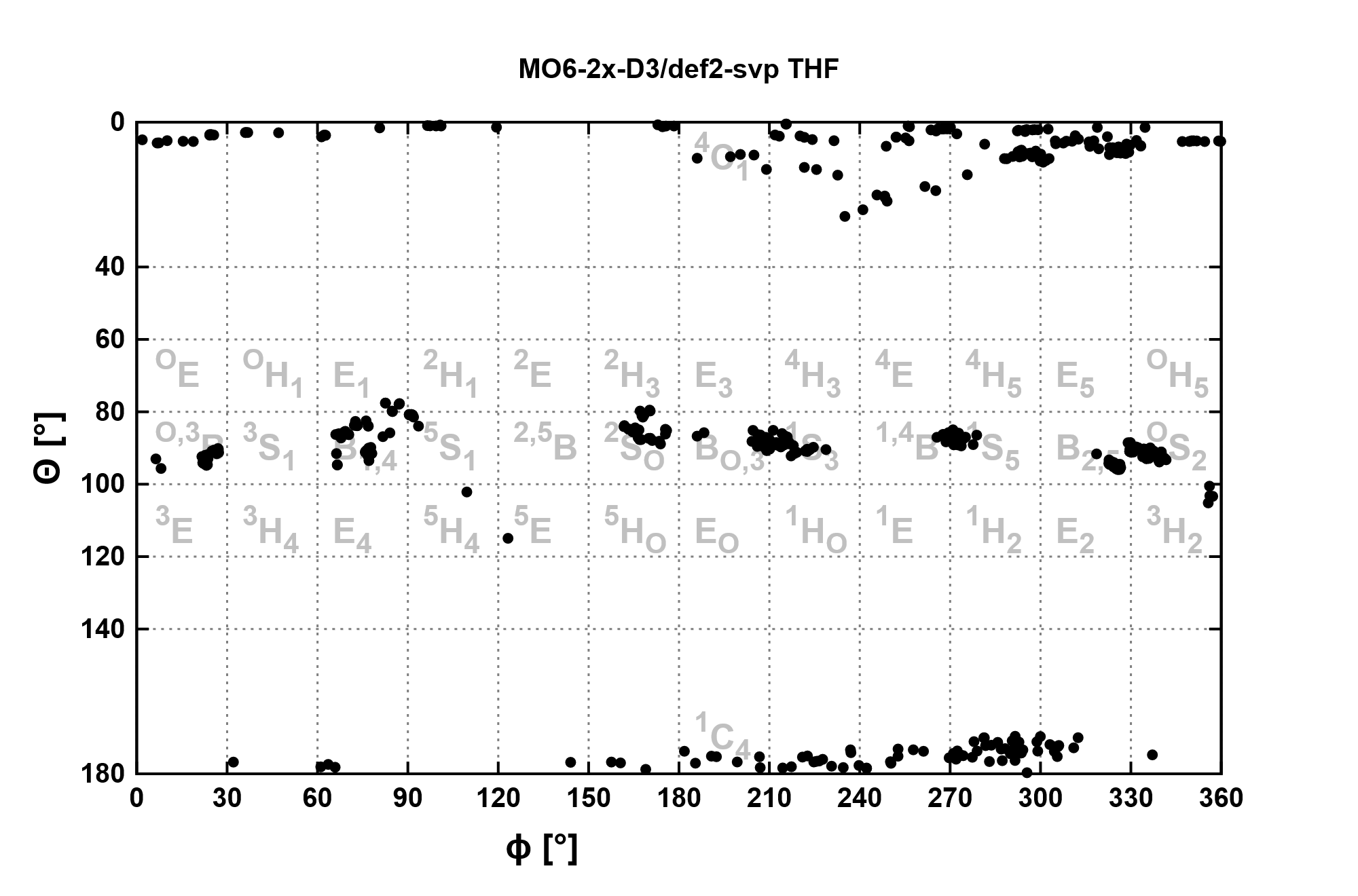

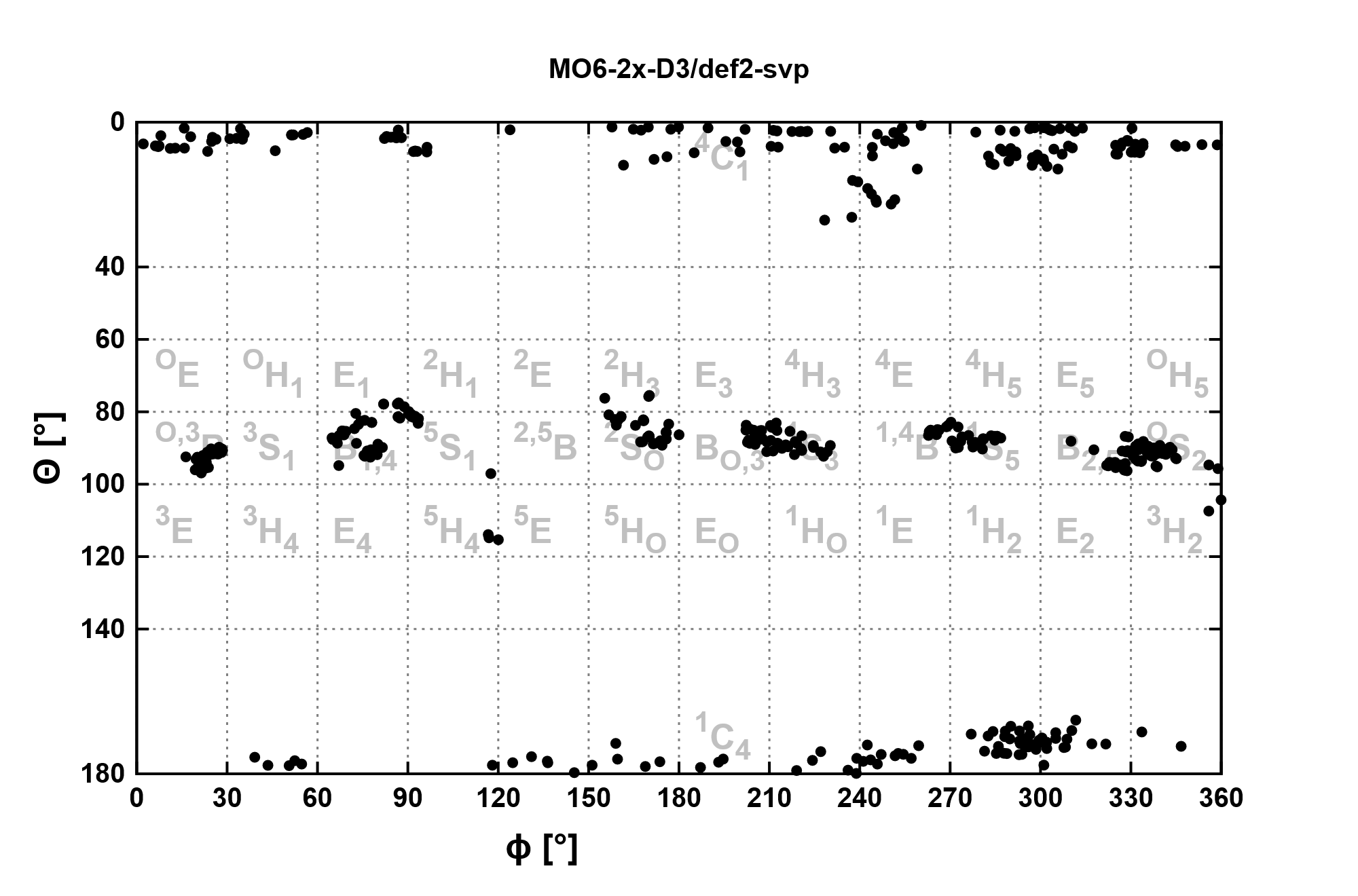


**Figure S2:** Refinement of 504 (no solvent, THF, CH_3_OH, DMSO and H_2_O with increasing dielectric constant) structures at the M06-2X-D3/def2-svp level of theory for different solvents treated using CPCM.


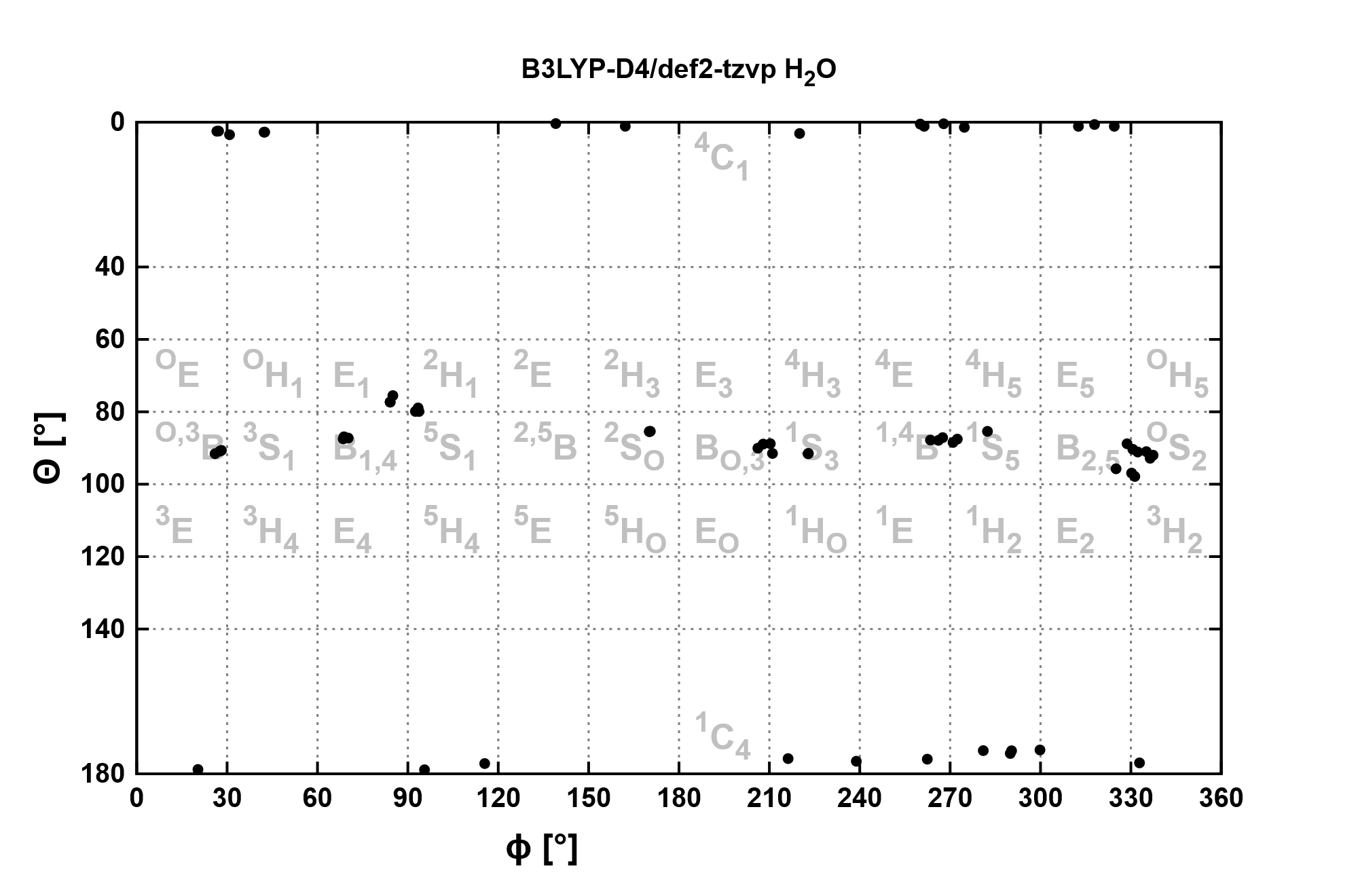

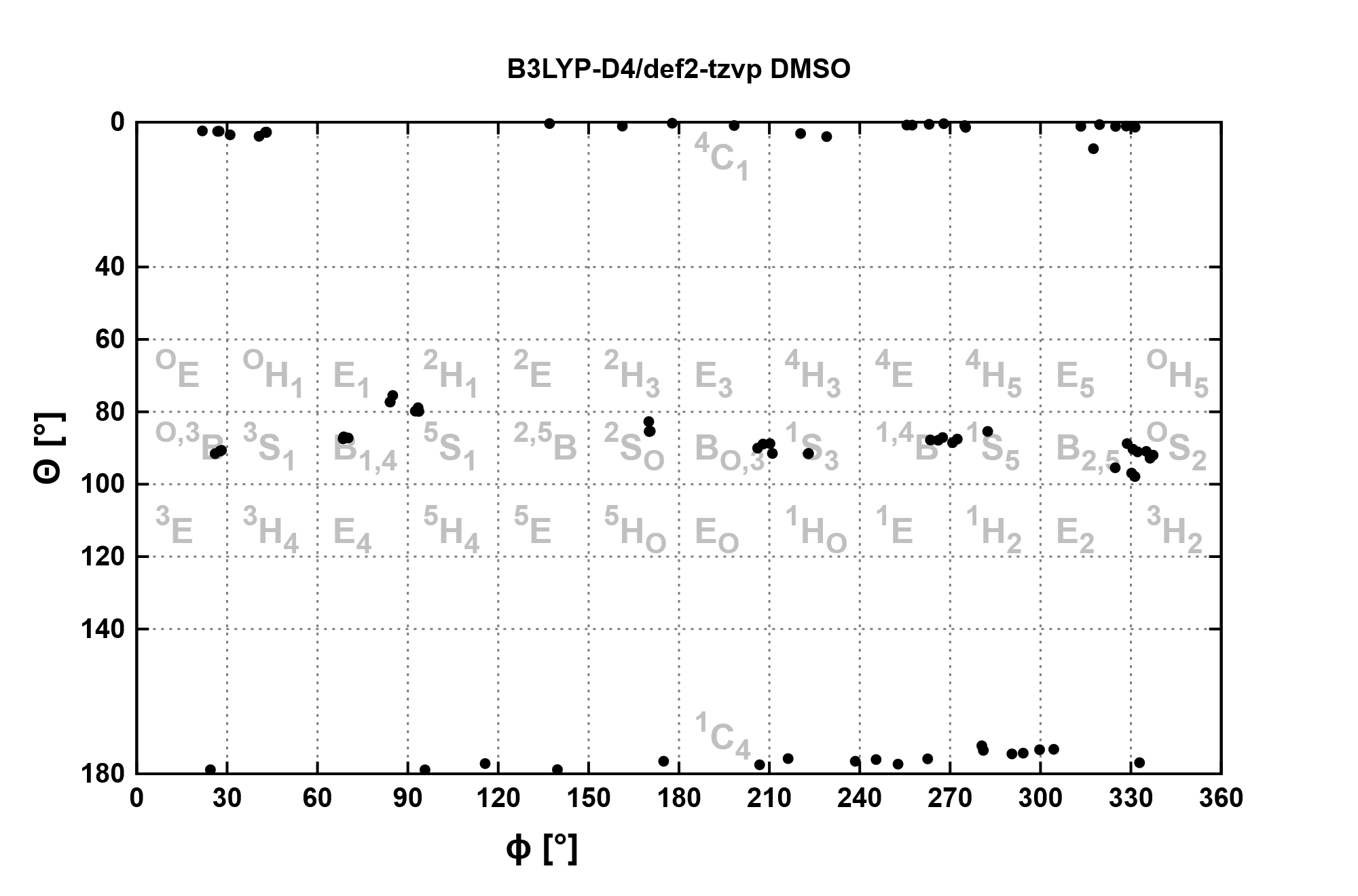

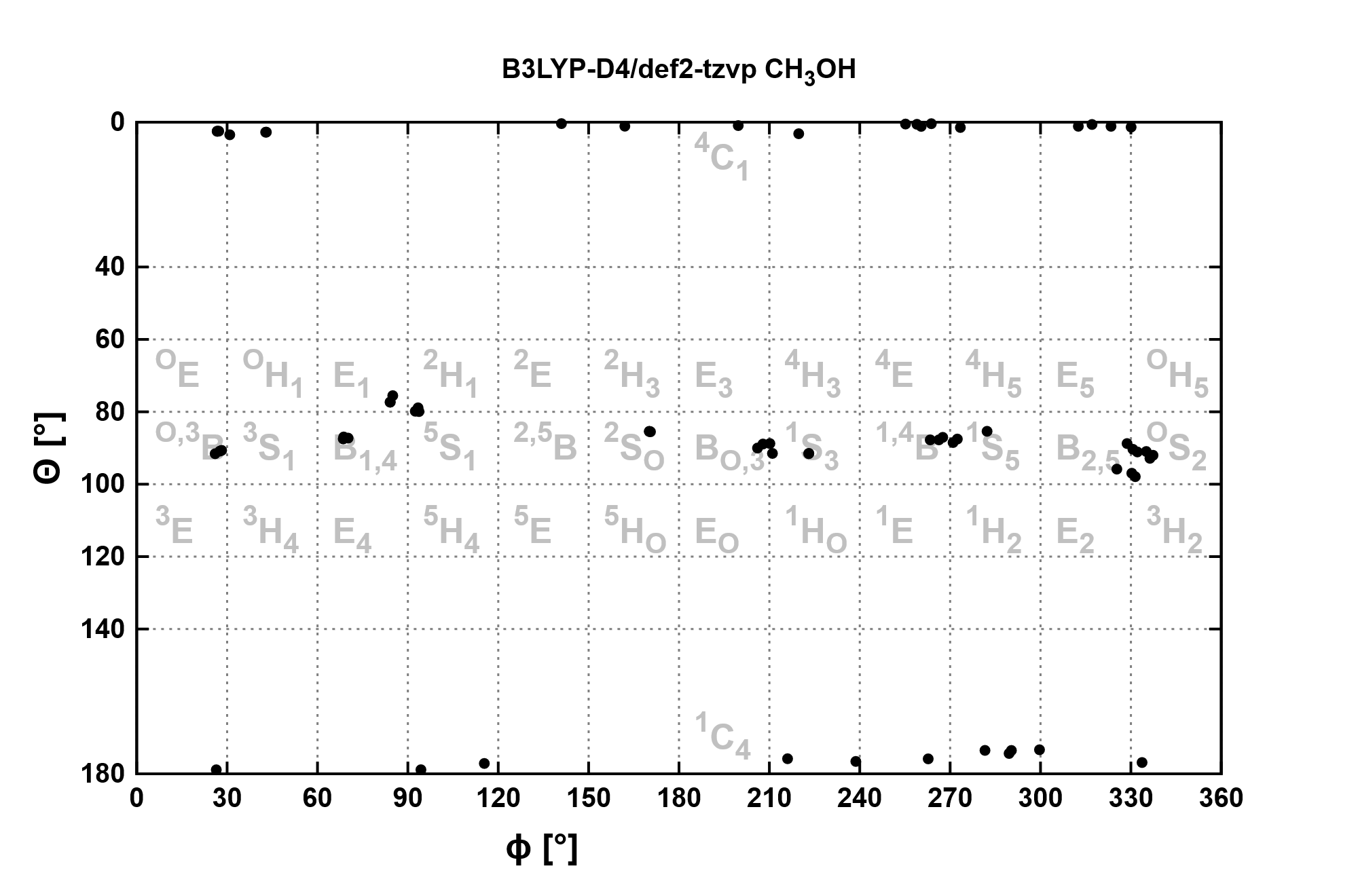

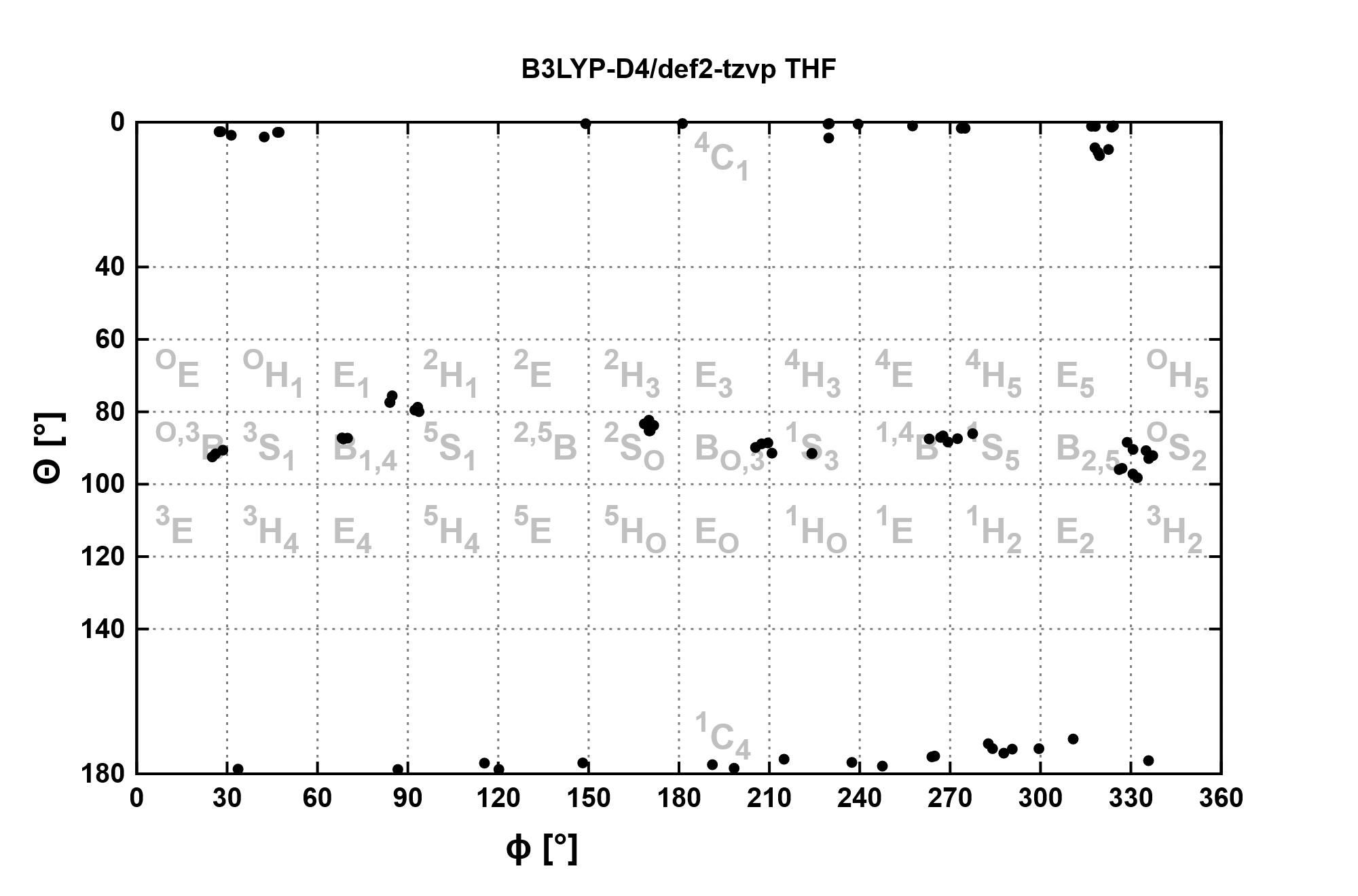

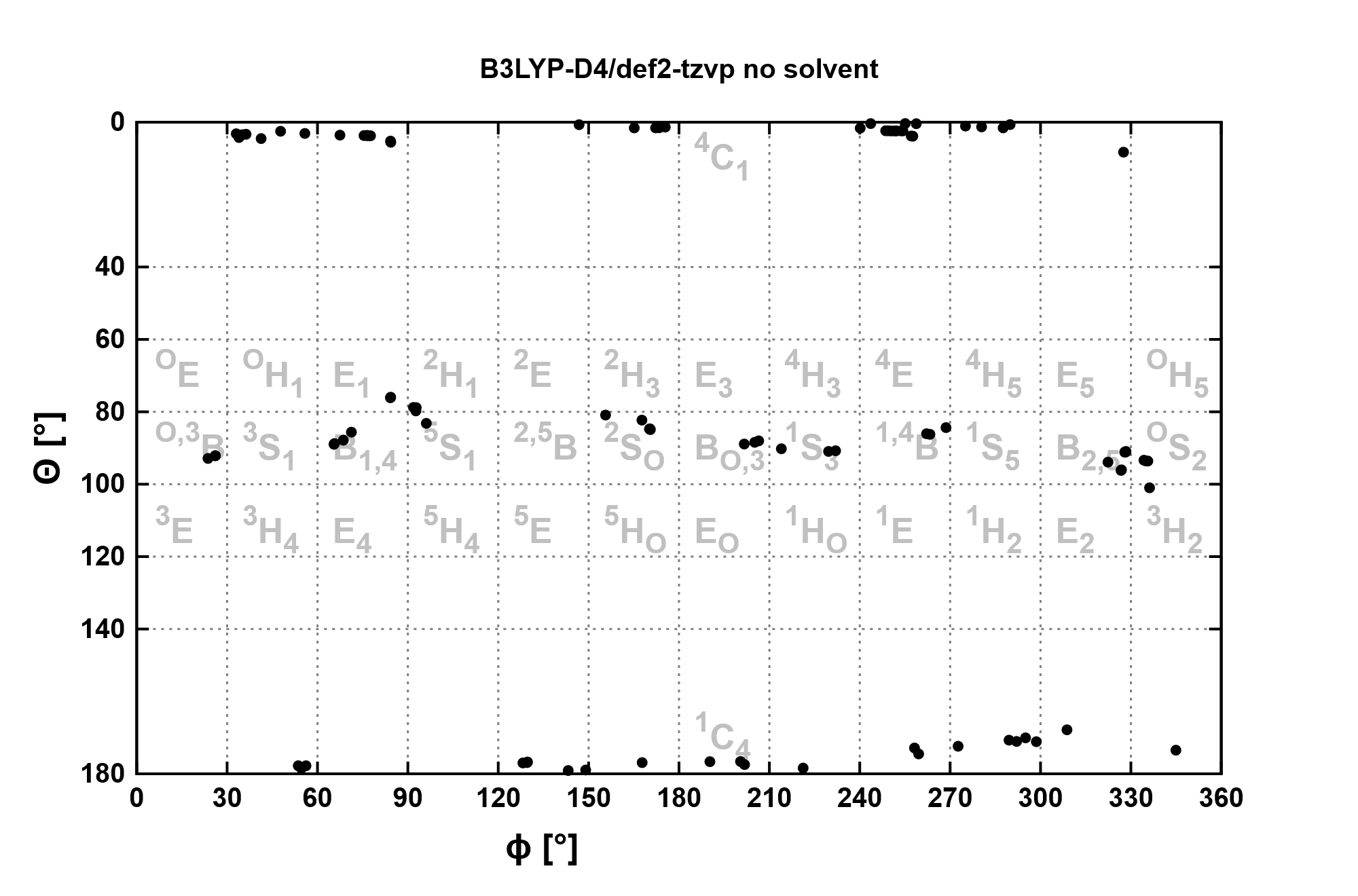


**Figure S3:** Refinement of 86 (no solvent), 82 (THF), 66 (CH_3_OH), 81 (DMSO) and 62 (H_2_O) (with increasing dielectric constant) structures at the B3LYP-D4/def2-tzvp level of theory.


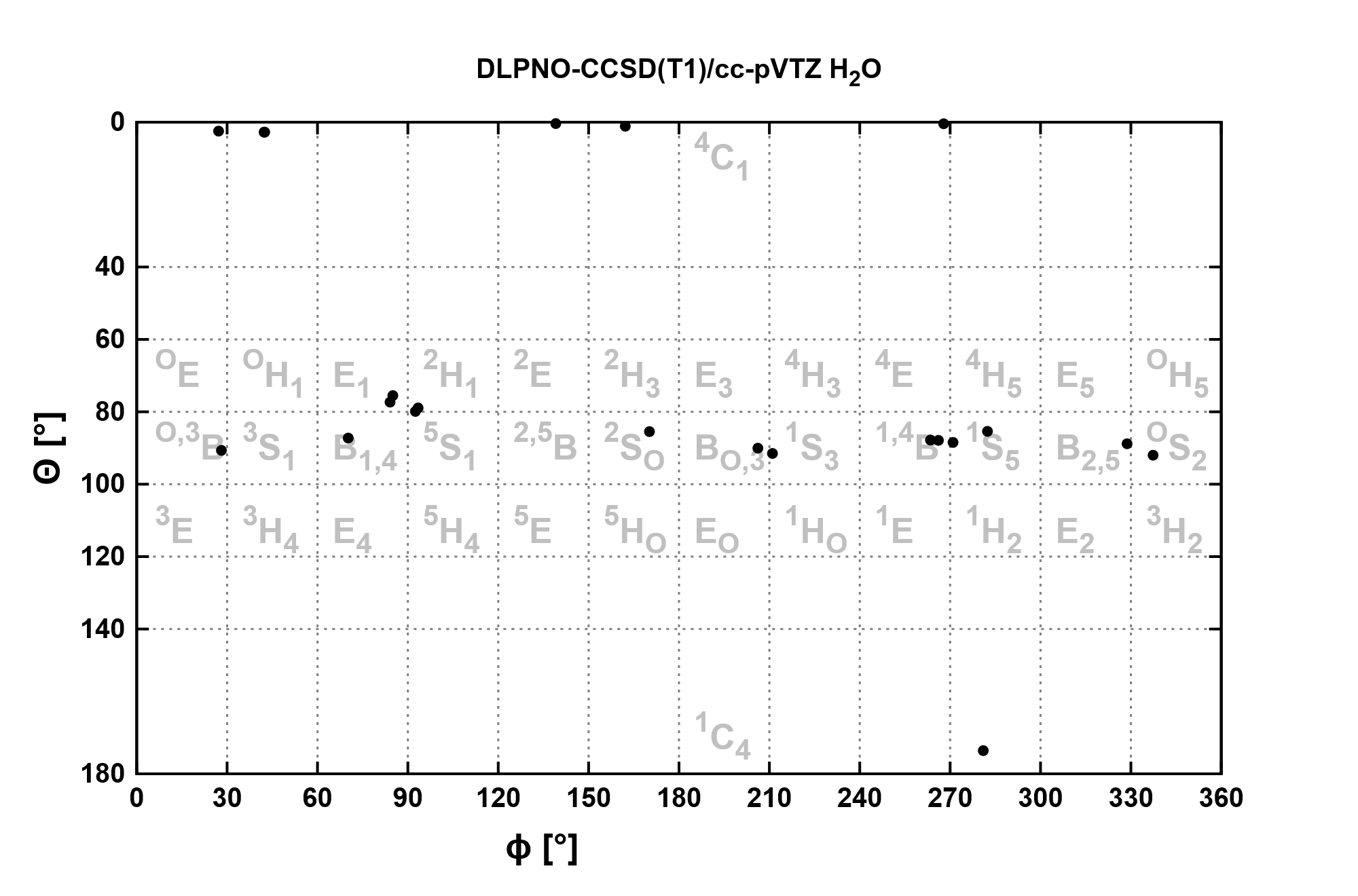

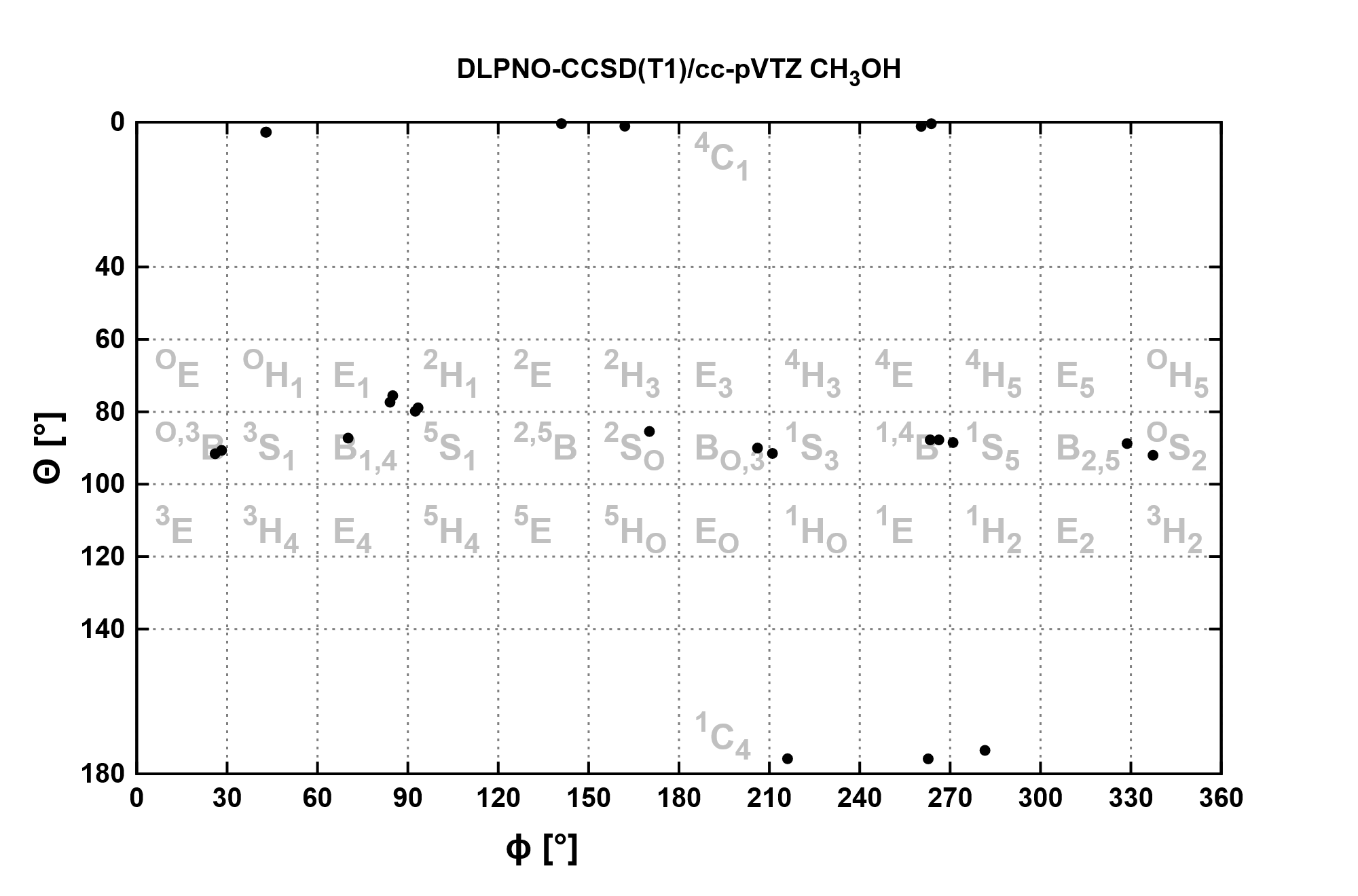

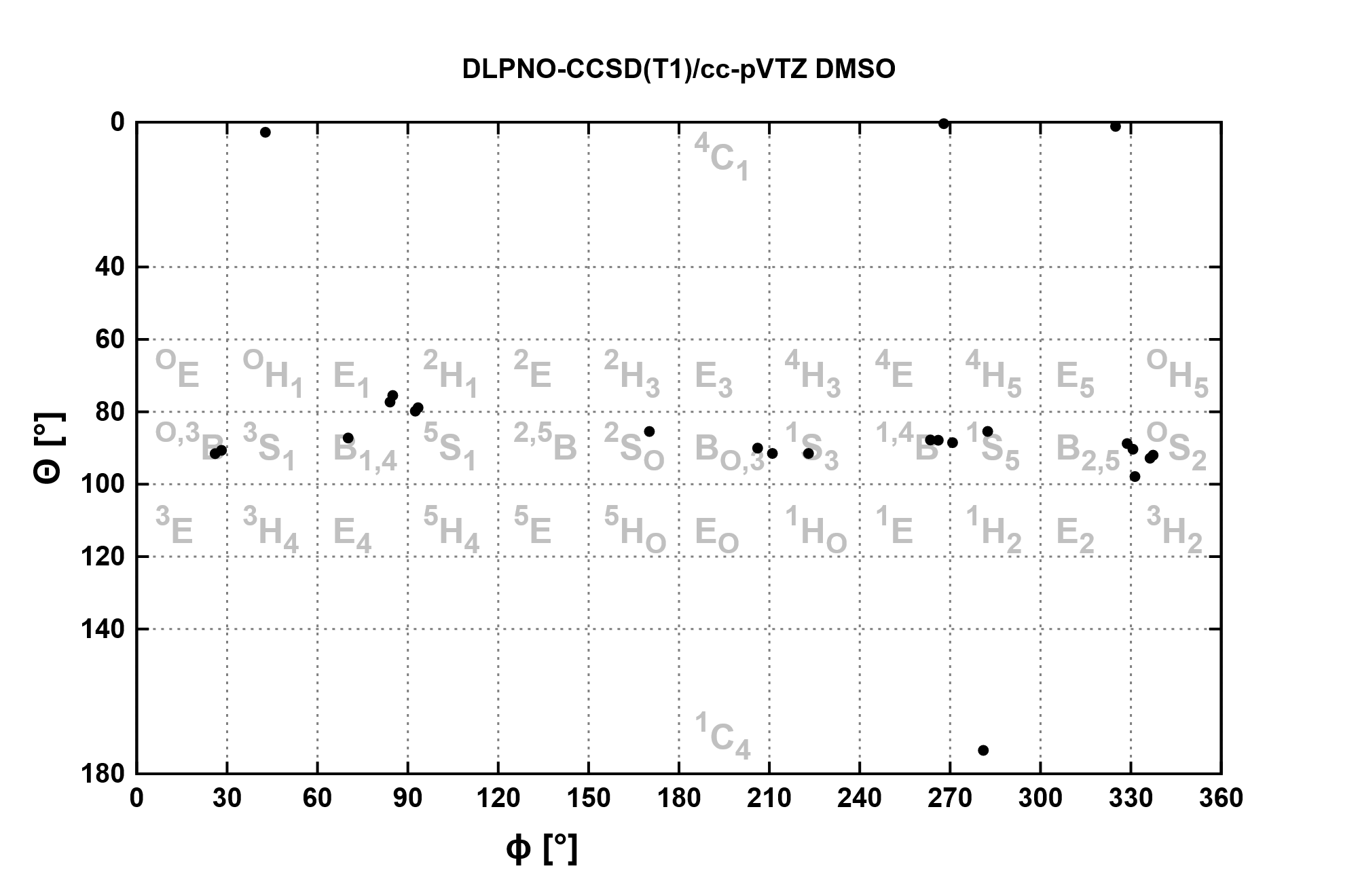

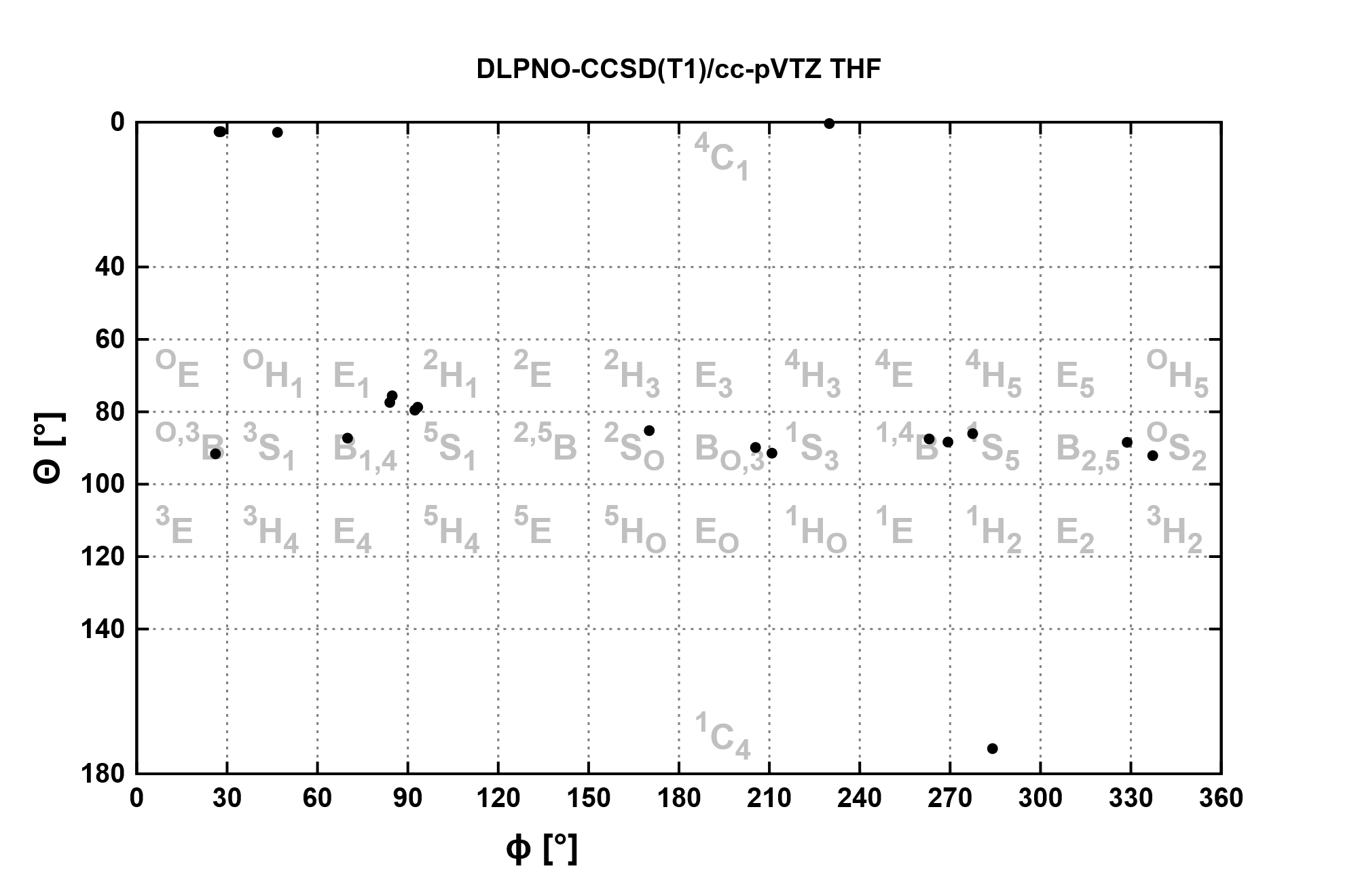

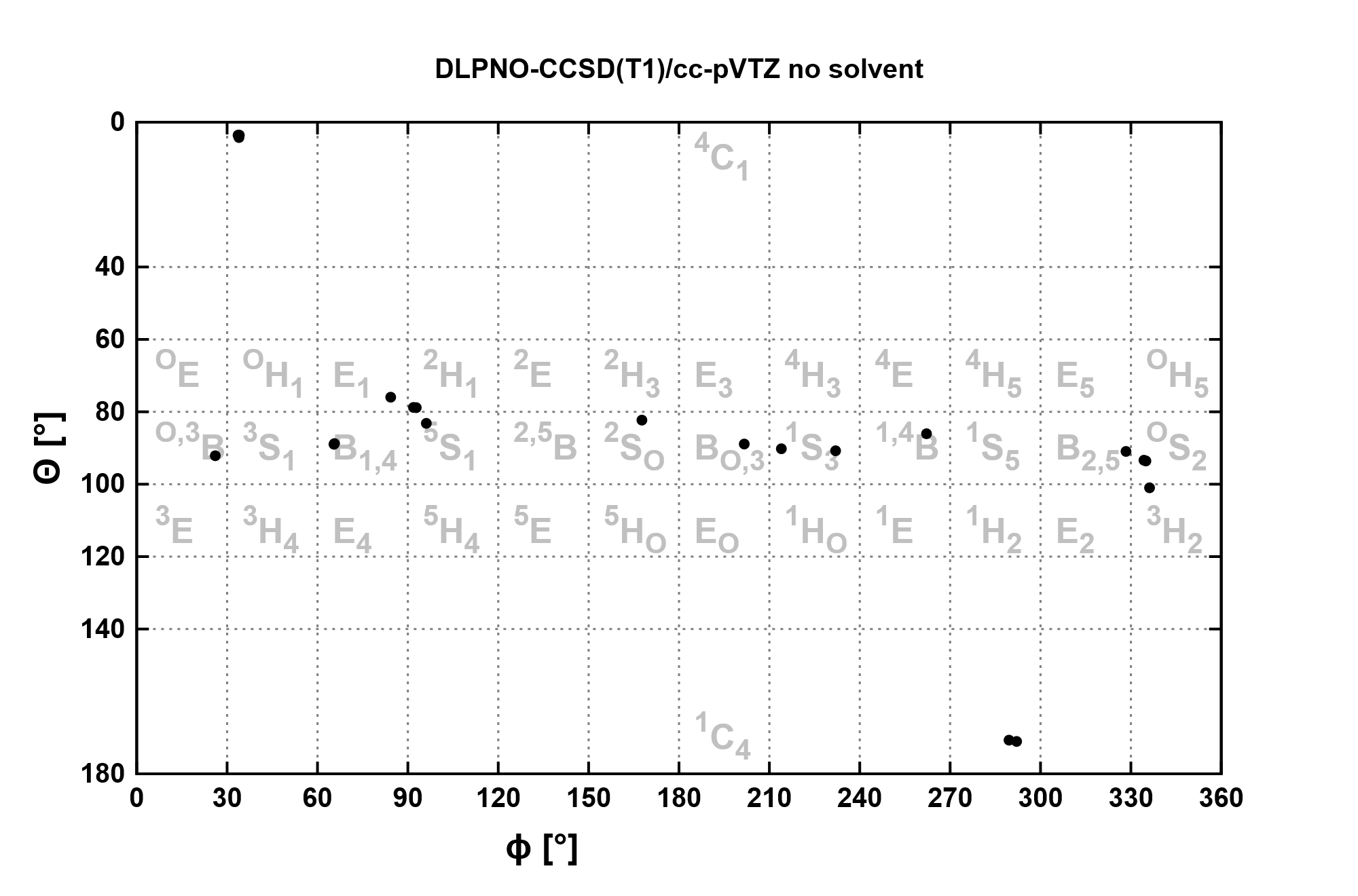
**Figure S4.** Final ensemble of 44 (no solvent), 19 (THF), 24 (CH_3_OH), 24 (DMSO) and 22 (H_2_O) lowest energy structures for which DLPNO-CCSD(T1)/cc-pVTZ energies have been evaluated. Note that for the no solvent case, a larger number of structures has been selected to account for more conformers that differ in their side chain conformations for comparison with the experimental microwave data.

## *O*-(2,3,4,6-Tetra-*O*-acetyl-α-d-altropyranosyl) trichloroacetimidate (2)

This subchapter presents Mercator plots of the conformer ensembles of *O*-(2,3,4,6-Tetra-*O*-acetyl-α-d-altropyranosyl) trichloroacetimidate (**2**), obtained at different levels of theory. All calculations have been carried out using a development version of the ORCA 6.0 program package.^[1]^ Figure S5 illustrates the results after an initial conformational search via GOAT at the xTB level of theory.^[2]^ The first refinement of the obtained conformers was carried out at the M06-2X-D3/def2-svp level of theory for CDCl_3_ (using CPCM)^[3]^ and the results are shown in Figure S6. Figure S7 displays the ensembles calculated at the B3LYP-D4/def2-tzvp level of theory,^[4]^ while Figure S8 summarizes the lowest energy structures for which DLPNO-CCSD(T1)/cc-pVTZ^[5]^ energies have been evaluated.


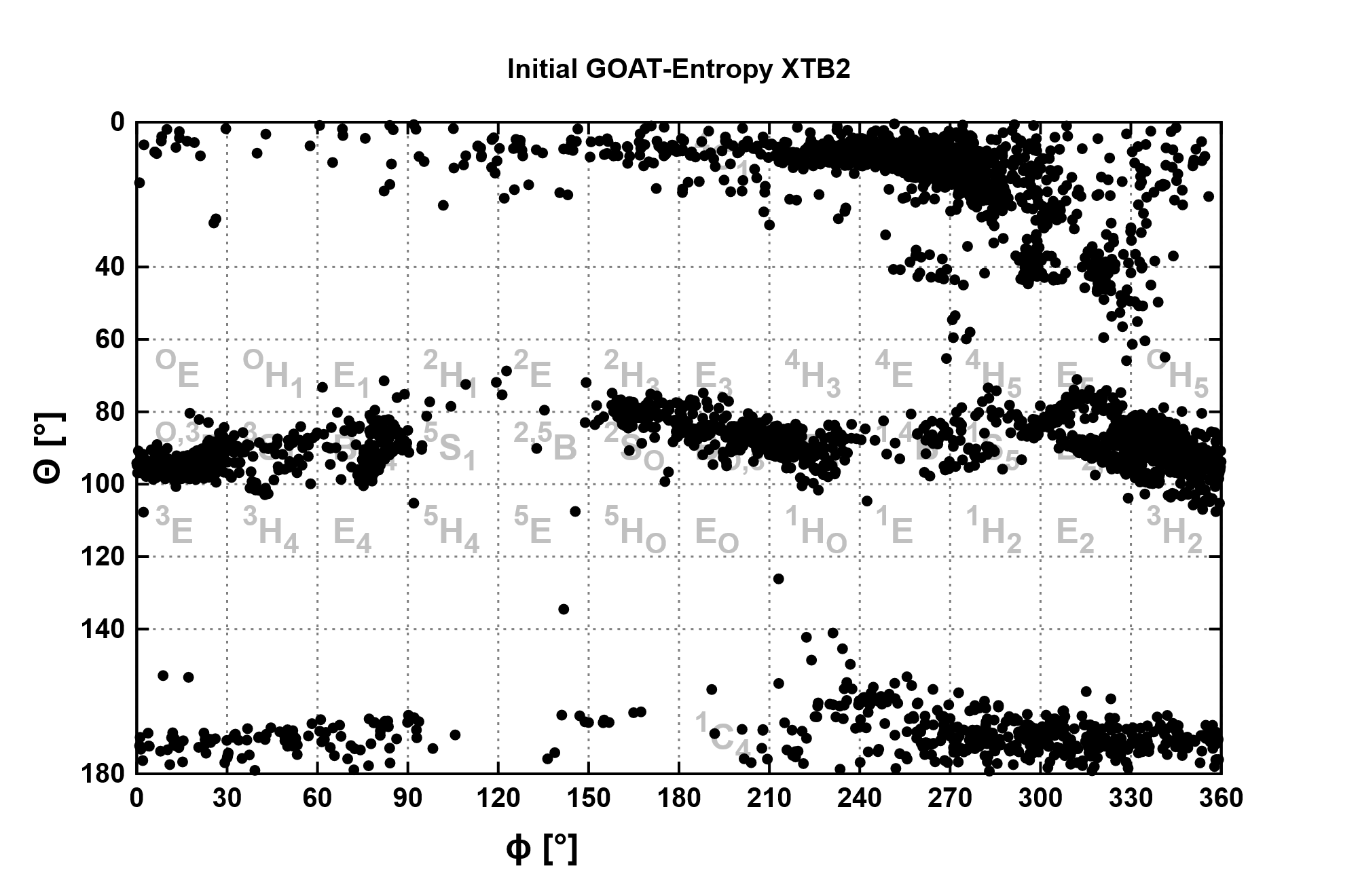
**Figure S5.** GOAT ensemble (4702 structures) obtained at the xTB level of theory.


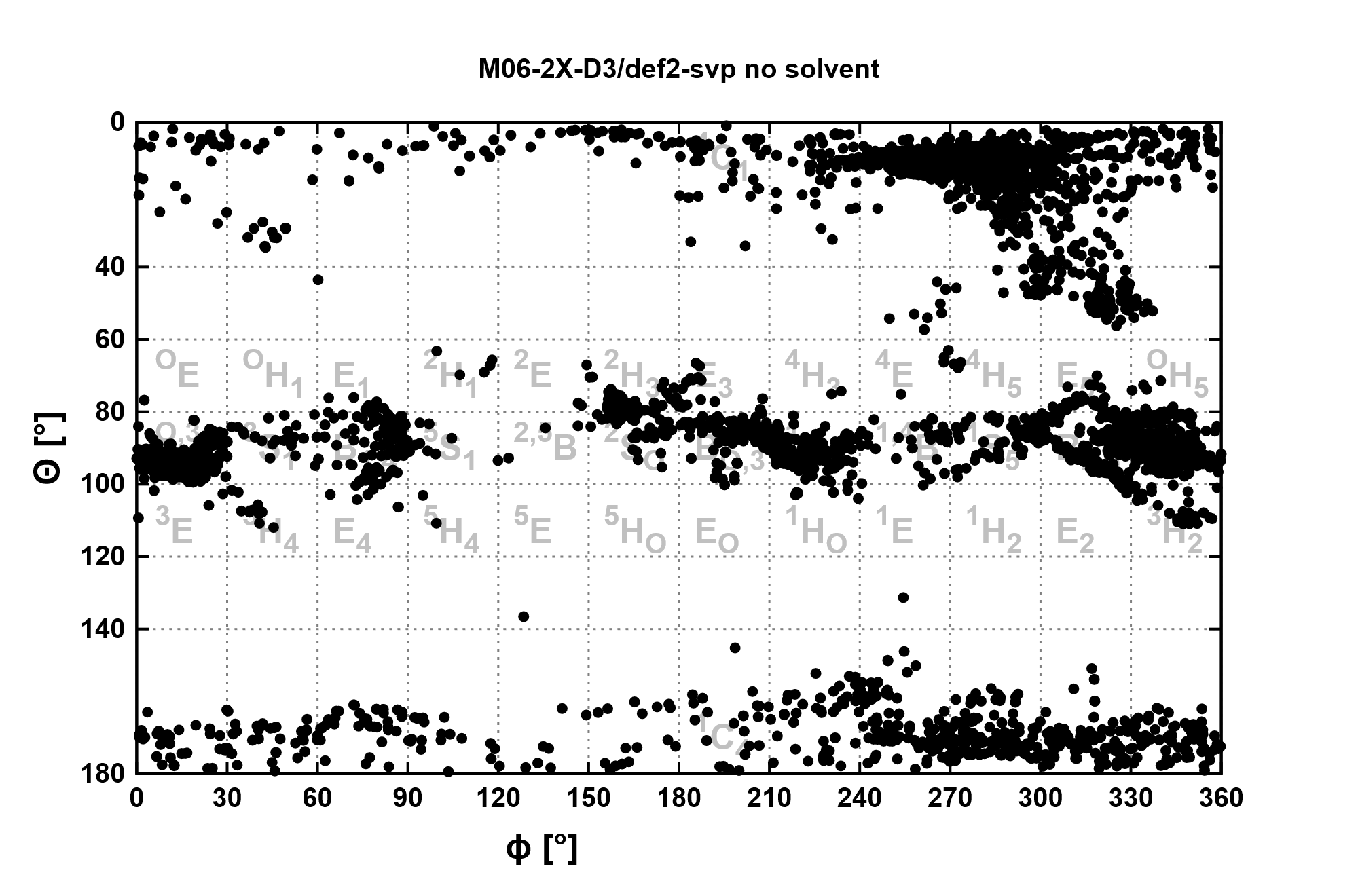


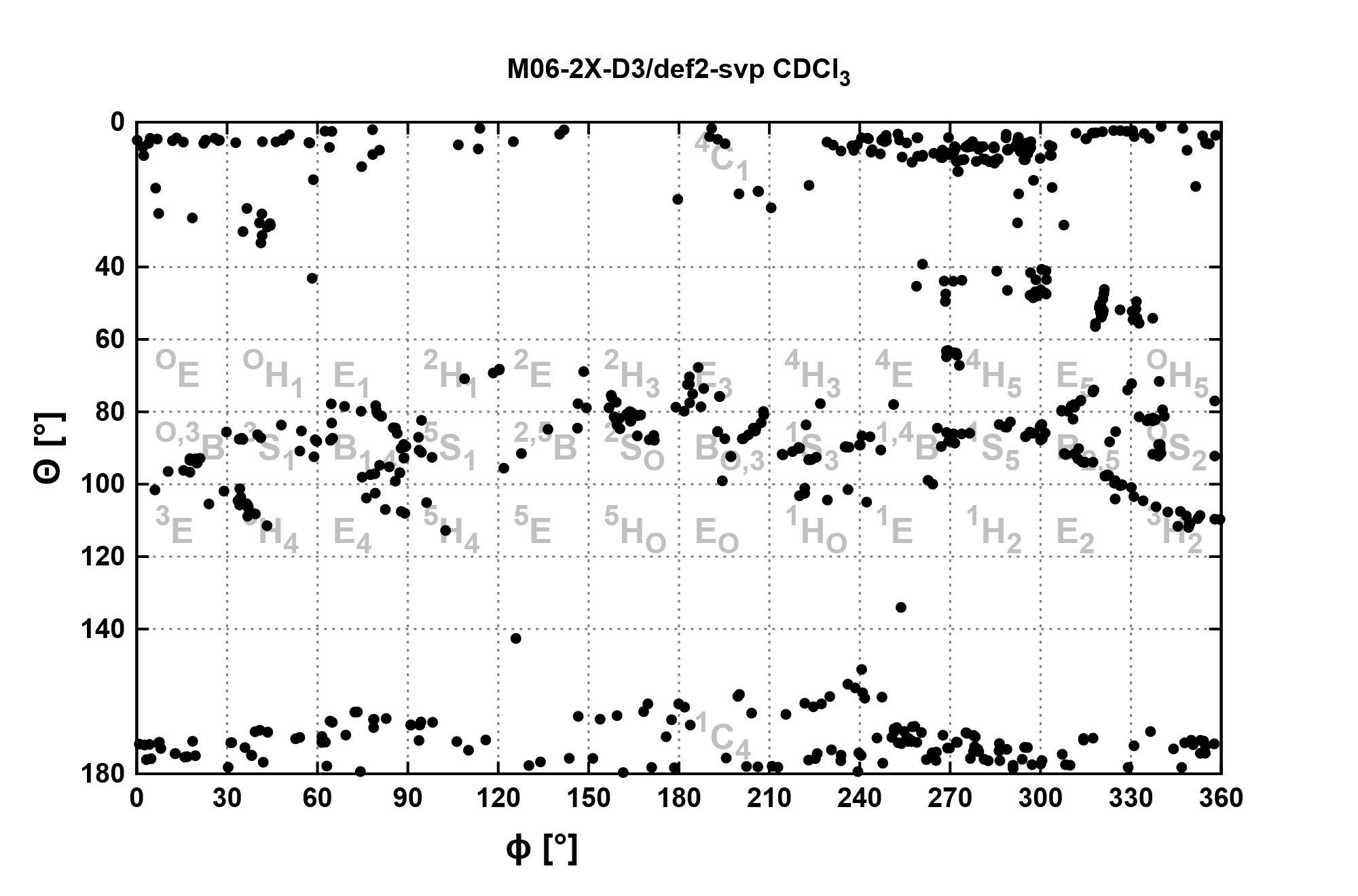


**Figure S6.** Refinement of 722 (CDCl_3_) / 4702 (no solvent) structures at the M06-2X-D3/def2-svp level of theory for different solvents treated using CPCM (note: in this case, a test with no selection at this level of theory has been carried out without CPCM).


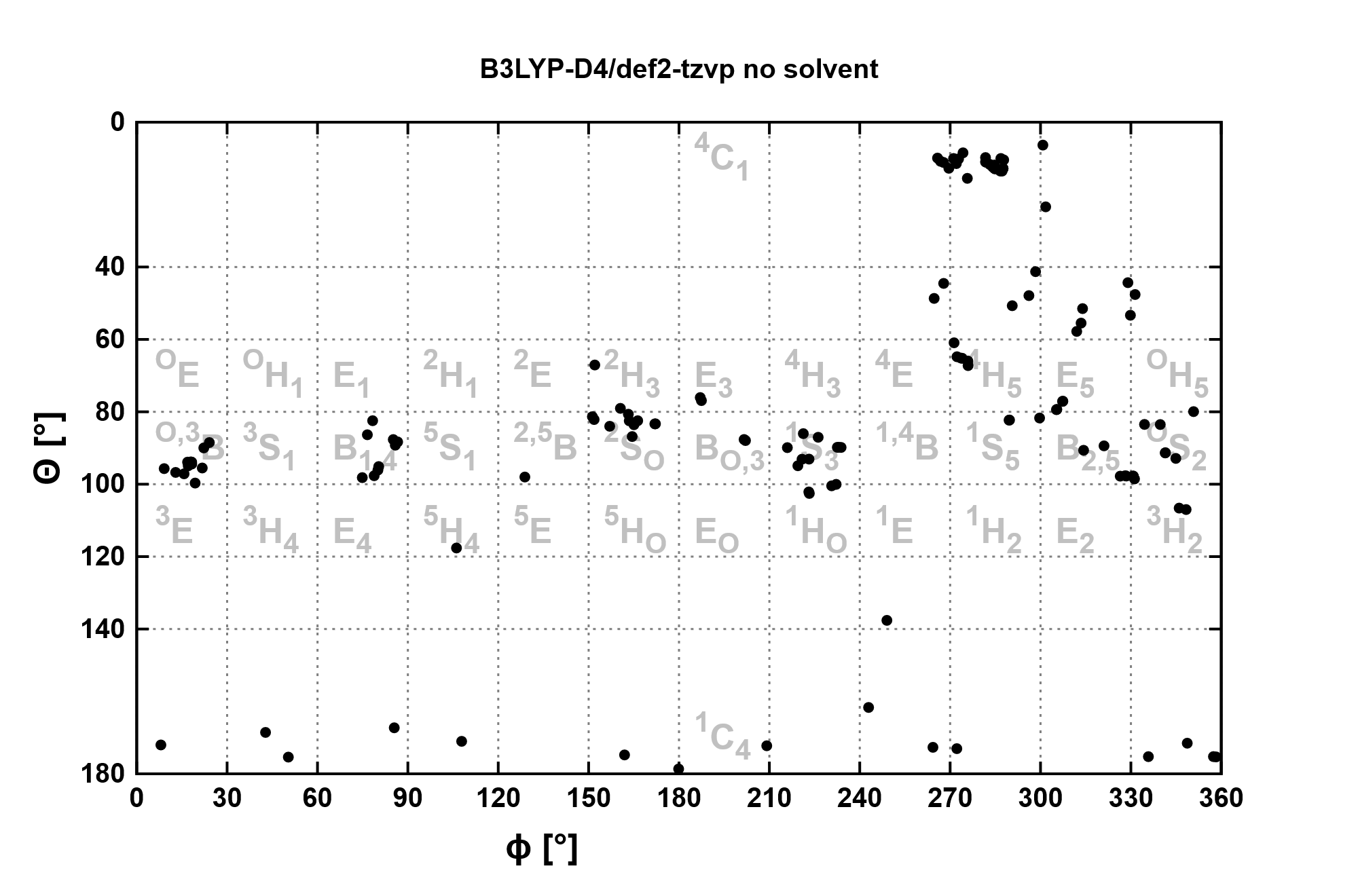

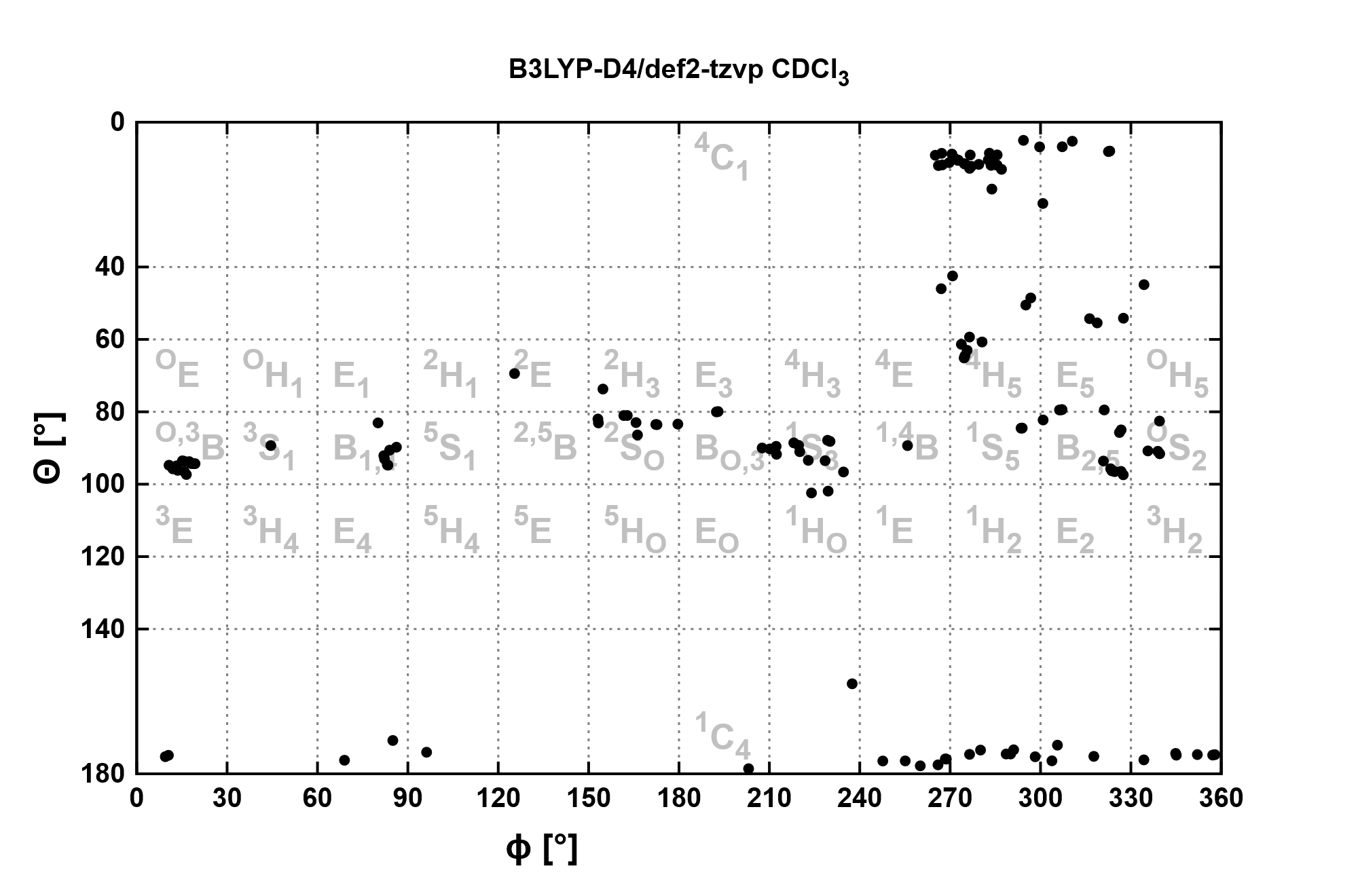


**Figure S7.** Refinement of 144 (CDCl_3_) / 141 (no solvent) structures at the B3LYP-D4/def2-tzpv level of theory.


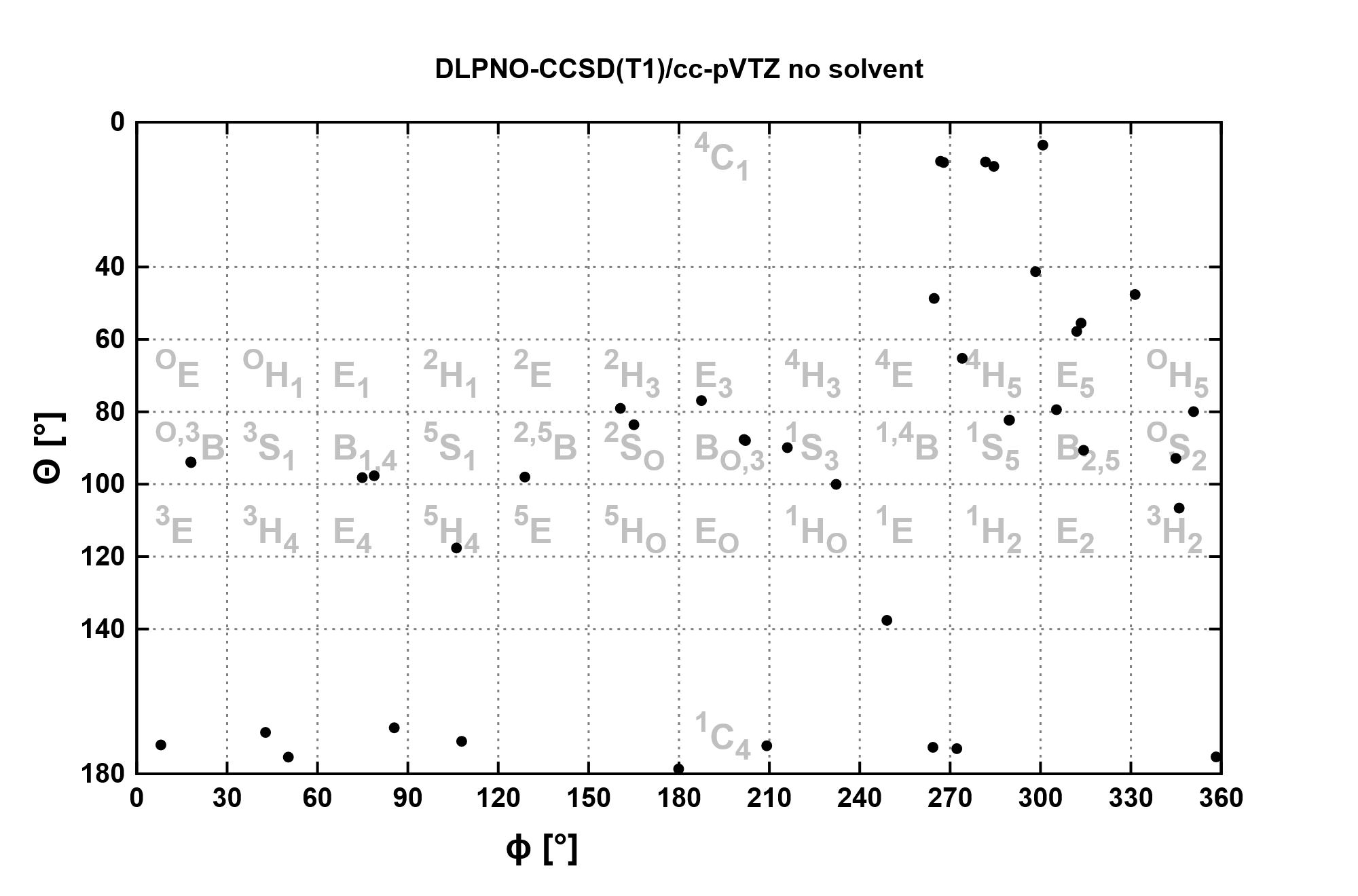

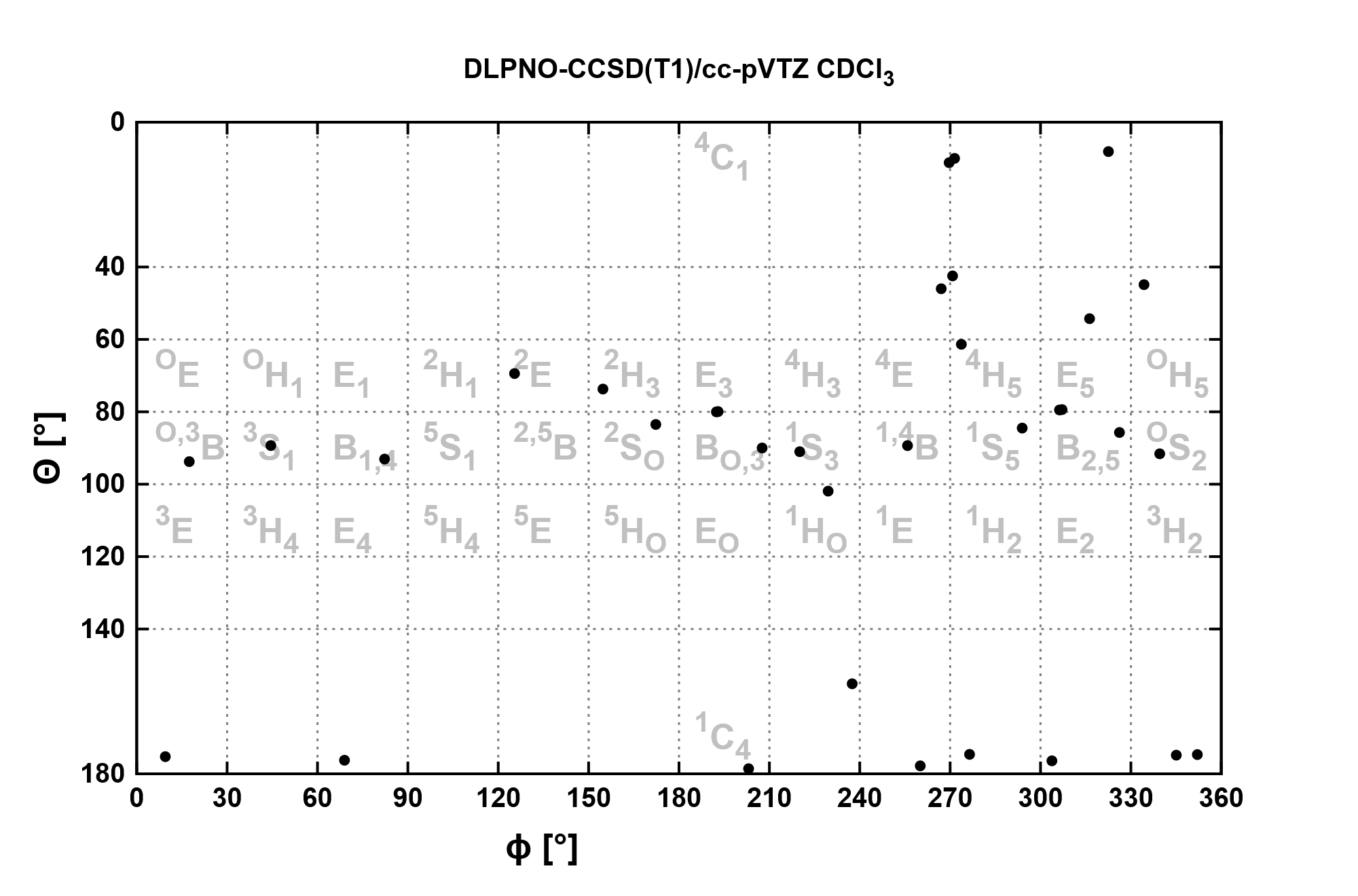


**Figure S8.** Final ensemble of 35 (CDCl_3_) / 43 (no solvent) lowest energy structures for which DLPNO-CCSD(T1)/cc-pVTZ energies have been evaluated.

In order to illustrate this in more detail, Figure S9 depicts the final ensemble of structures optimized at the B3LYP level of theory and the preceding structures optimized at lower levels of theory. This way, one can observe the changes upon increasing the level of theory from XTB to M06-2x-D3/svp to B3LYP-D4/tzvp and also compare the B3LYP results with and without CPCM. Here, clear differences upon refinement are visible as well as the general trend that higher levels of theory (refined functionals and corrections as well as larger basis sets) seem to deepen local minima effectively, reducing the scatter of the resulting structures. Furthermore, comparing results without and with CPCM (roughly speaking gas-phase vs. CDCl_3_) also seems to indicate a different bias. If solvent models are applied although the results do not change dramatically, which is probably because of screening of long-range electrostatic effects when using implicit solvation.


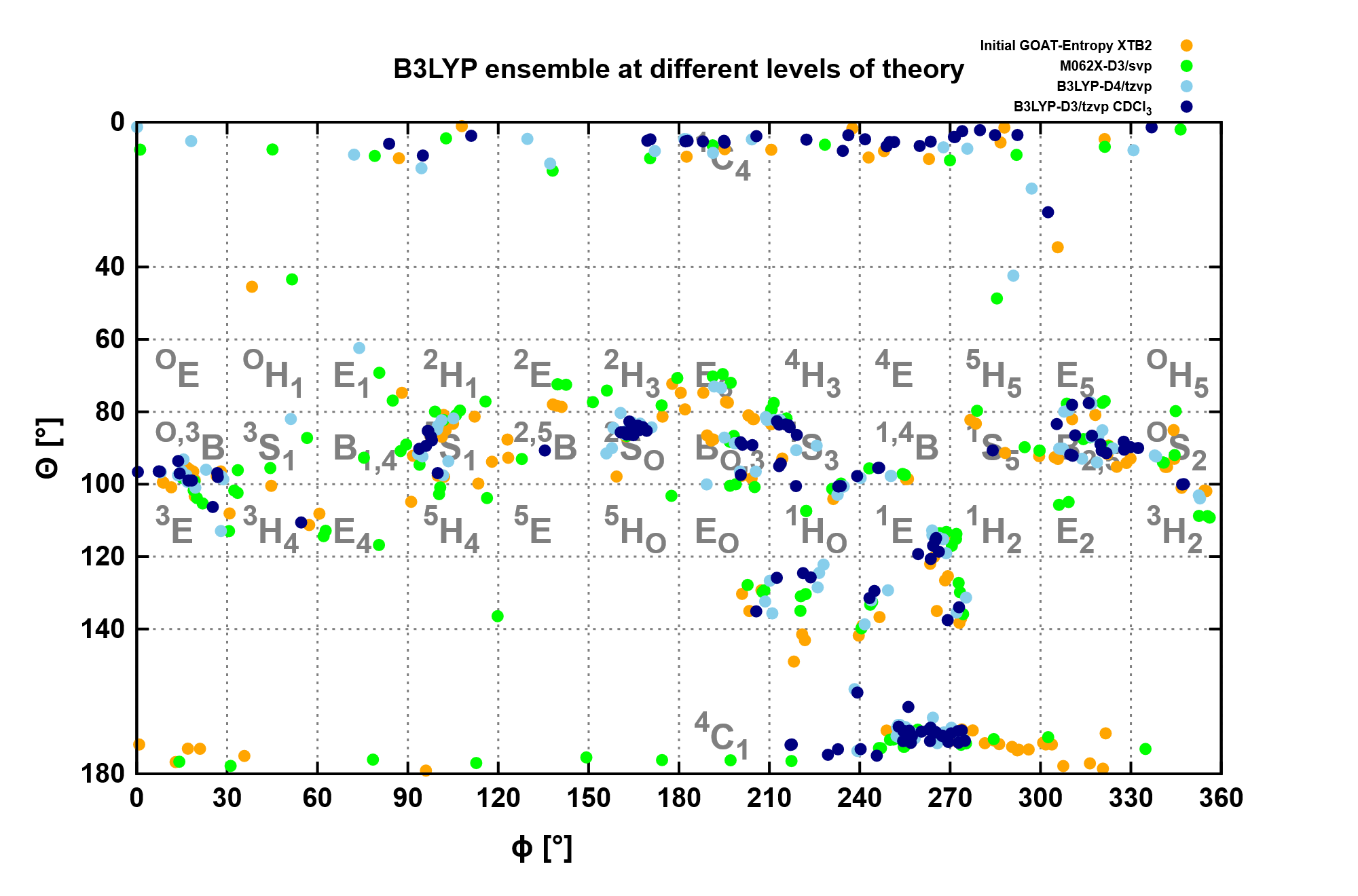
**Figure S9.** Mercator plot of the puckering angles of the ensemble of **2**. All structures of the final B3LYP (no solvent) ensemble are shown together with their preceding lower-level geometries (M06-2x and XTB) together with the B3LYP CPCM(CDCl_3_) ensemble.

## *O*-(2,3,4,6-Tetra-*O*-acetyl-β-d-altropyranosyl) trichloroacetimidate (3)

This subchapter presents Mercator plots of the conformer ensembles of *O*-(2,3,4,6-Tetra-*O*-acetyl-β-d-altropyranosyl) trichloroacetimidate (**3**), obtained at different levels of theory. All calculations have been carried out using a development version of the ORCA 6.0 program package.^[1]^ Figure S10 illustrates the results after an initial conformational search via GOAT at the xTB level of theory.^[2]^ The first refinement of the obtained conformers was carried out at the M06-2X-D3/def2-svp level of theory for CDCl_3_ (using CPCM)^[3]^ and the results are shown in Figure S11. Figure S12 displays the ensembles calculated at the B3LYP-D4/def2-tzvp level of theory,^[4]^ while Figure S13 summarizes the lowest energy structures for which DLPNO-CCSD(T1)/cc-pVTZ^[5]^ energies have been evaluated.


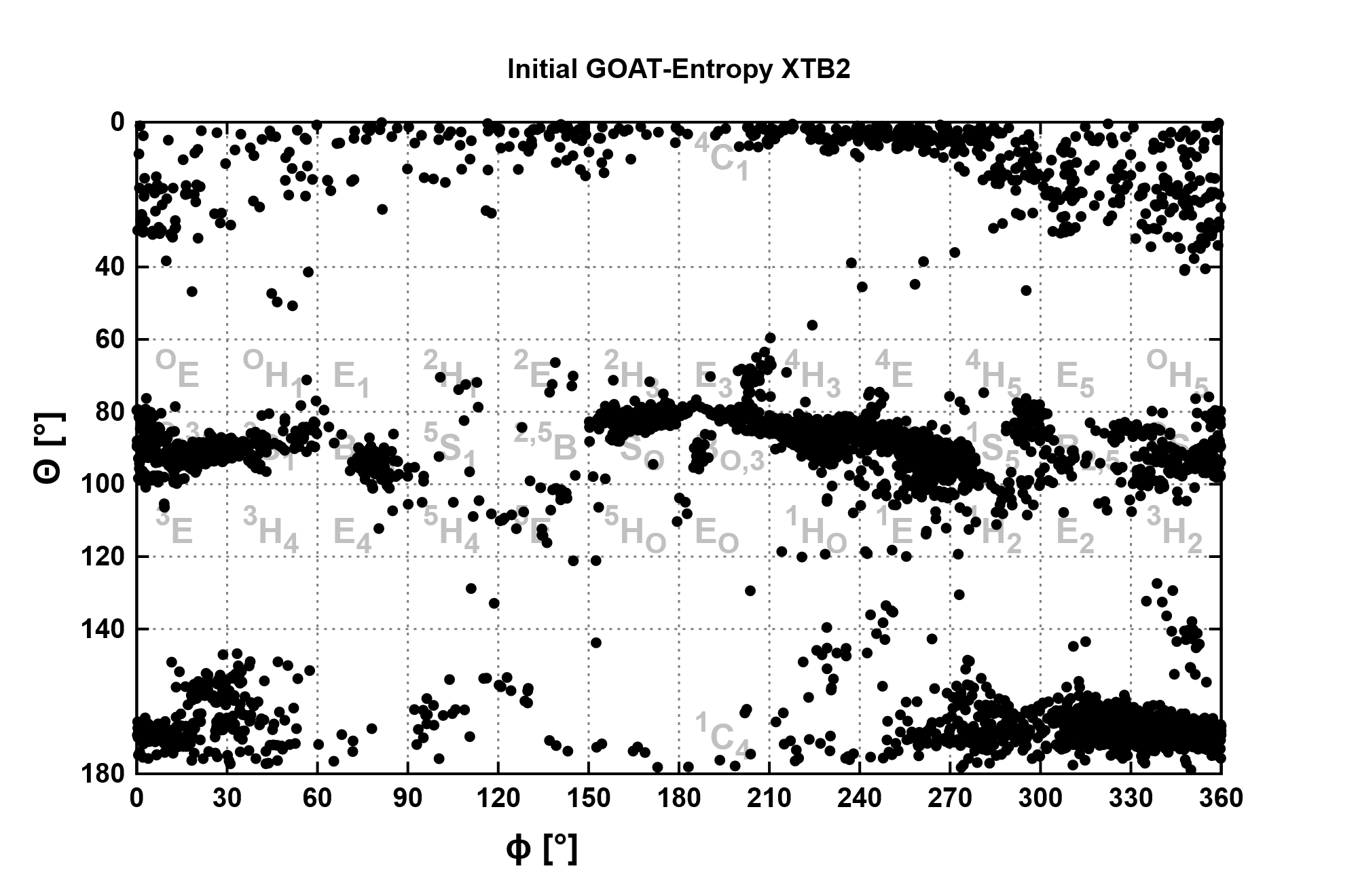


**Figure S10.** GOAT ensemble (5317 structures) obtained at the xTB level of theory.


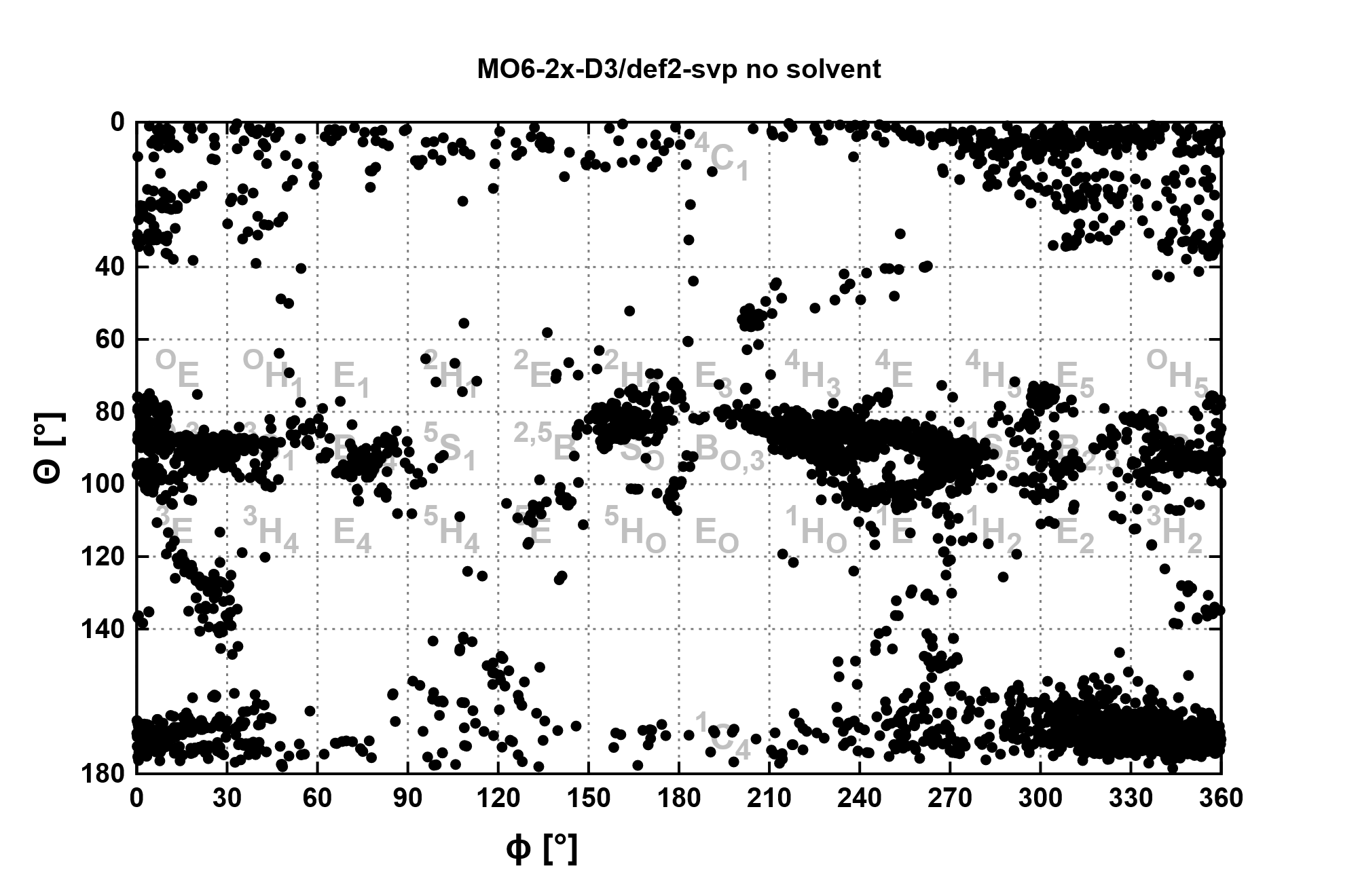


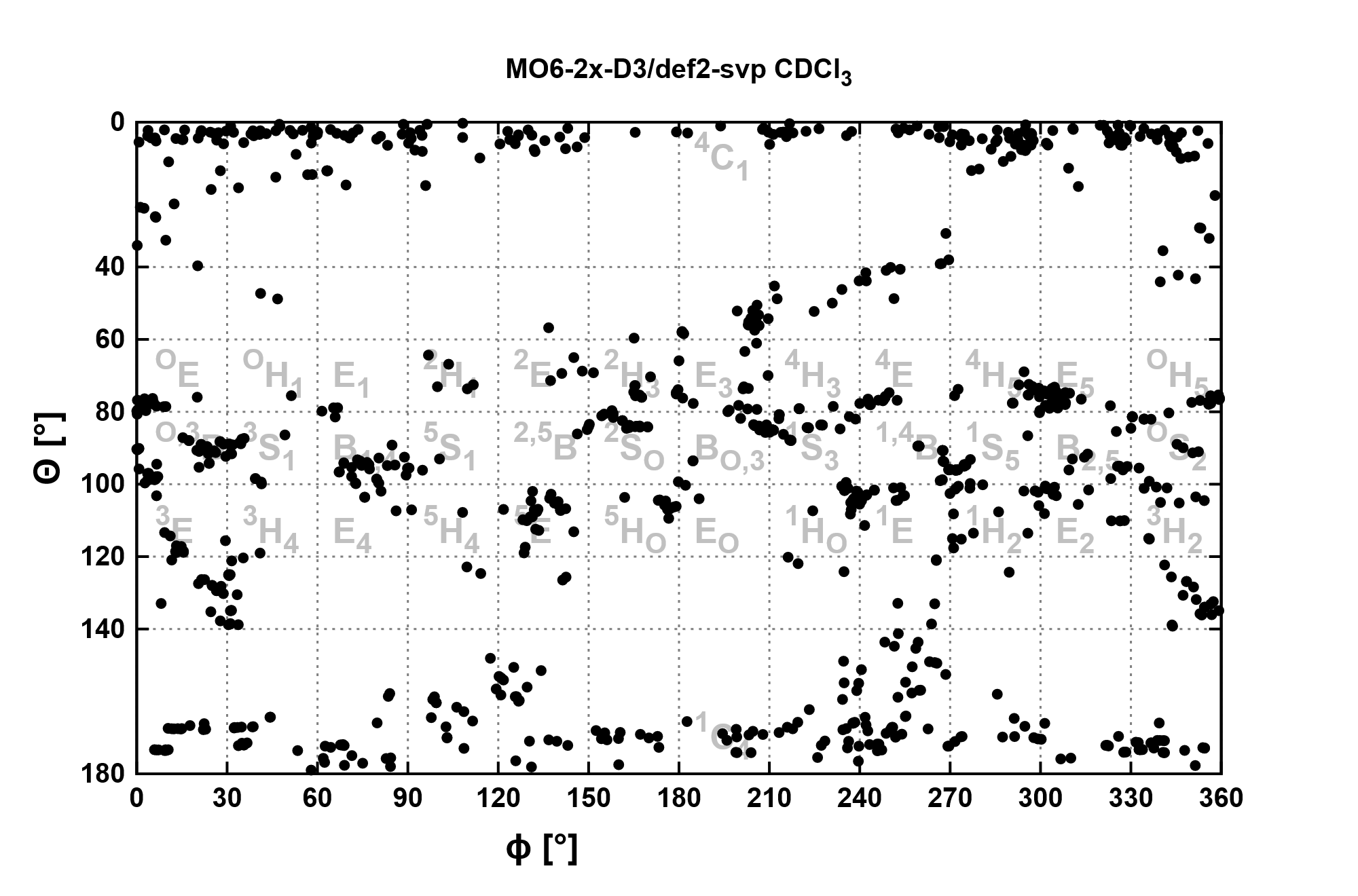


**Figure S11.** Refinement of 1012 (CDCl_3_) / 5317 (no solvent) structures at the M06-2X-D3/def2-svp level of theory for different solvents treated using CPCM (note: in this case, a test with no selection at this level of theory has been carried out without CPCM).


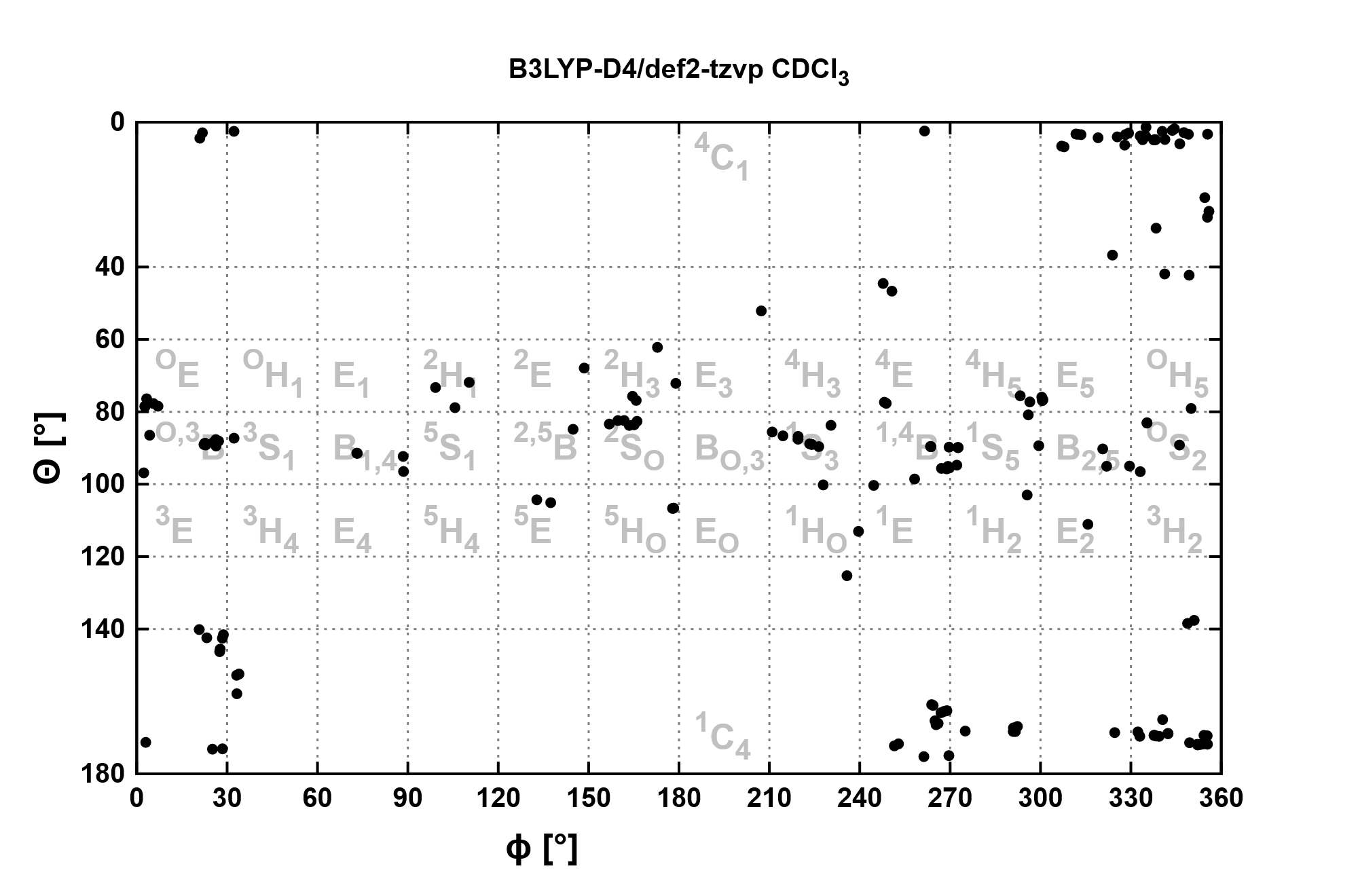

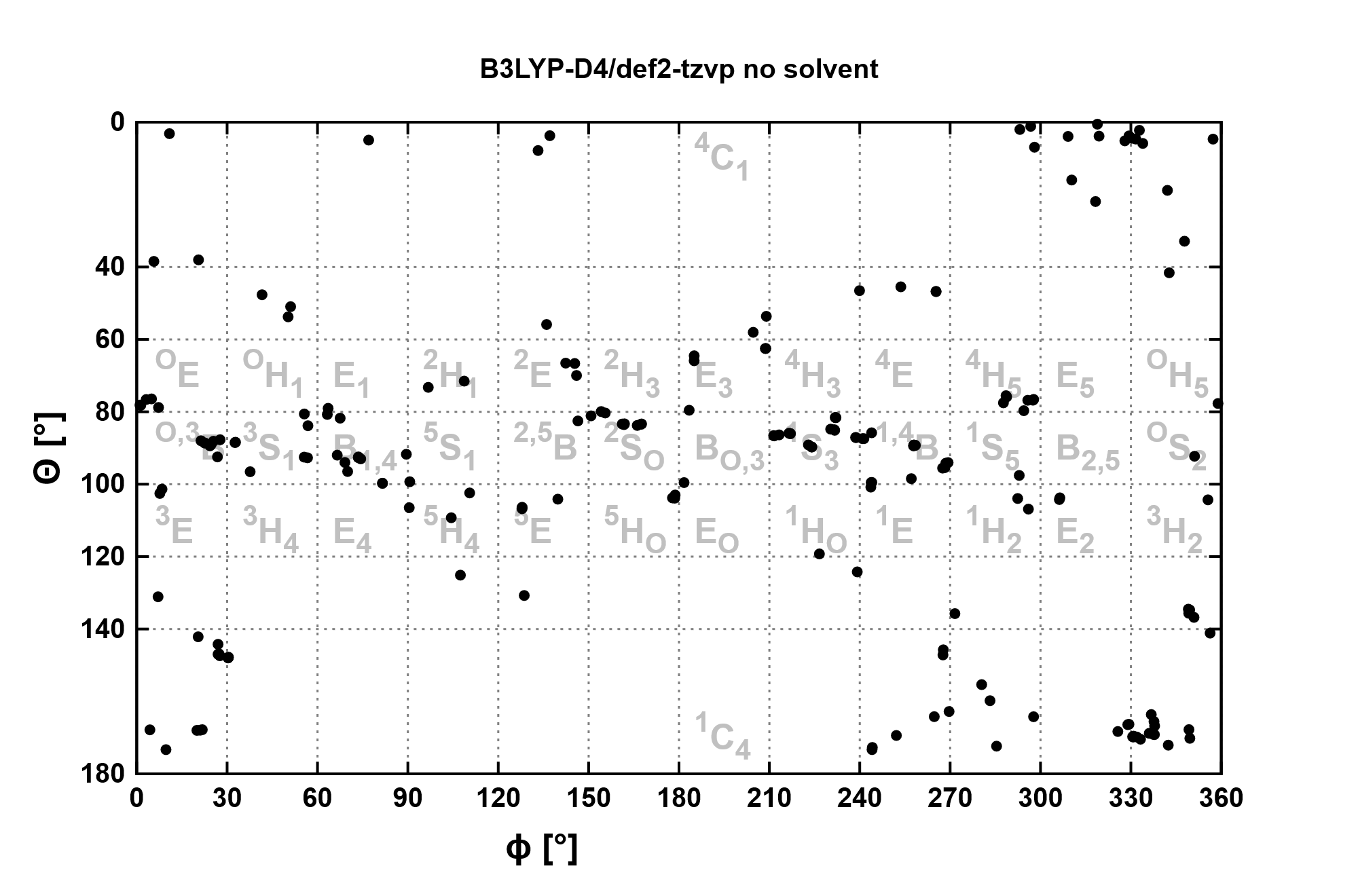
**Figure S12.** Refinement of 176 (CDCl_3_) / 203 (no solvent) structures at the B3LYP-D4/def2-tzpv level of theory.


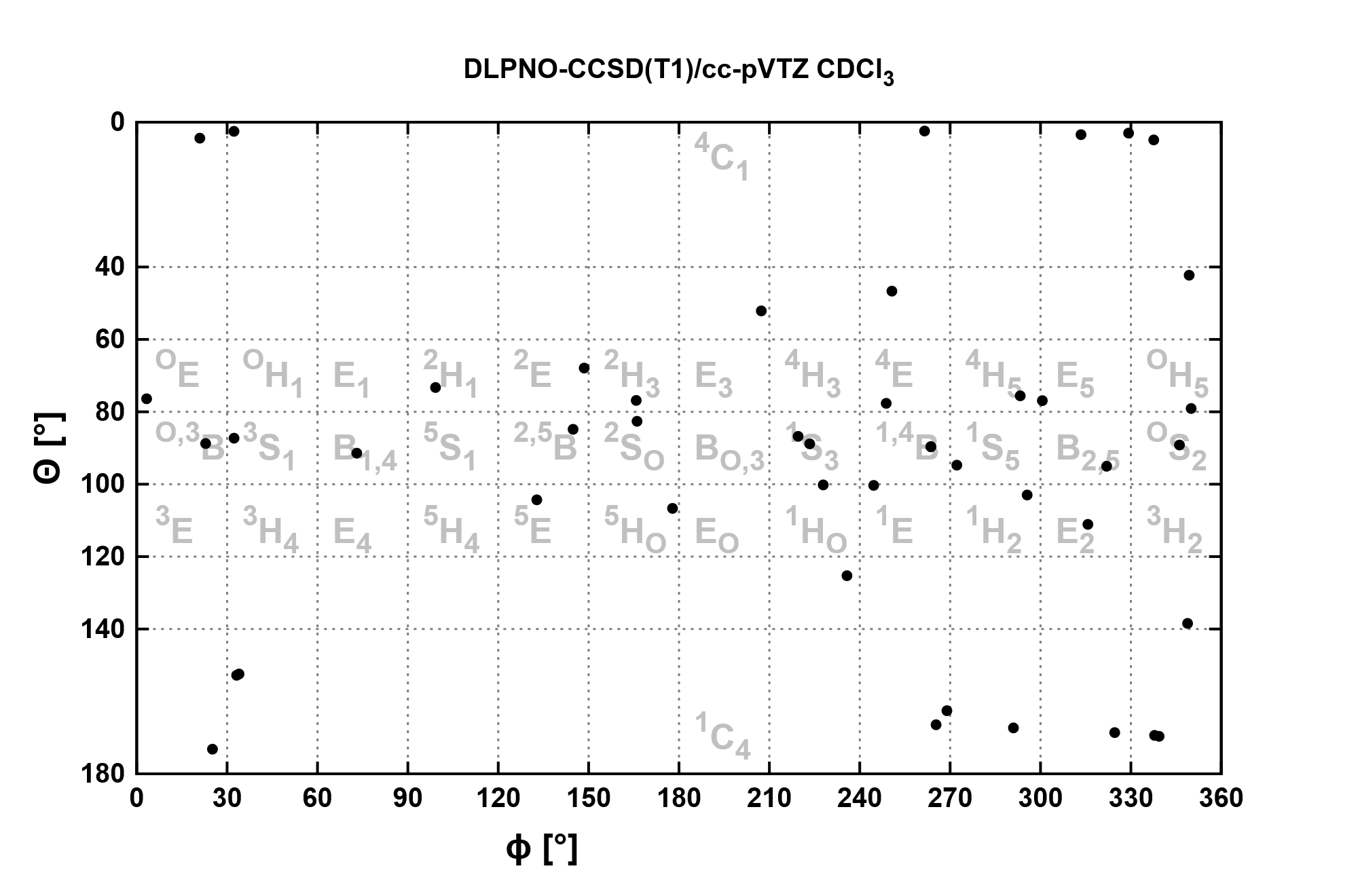

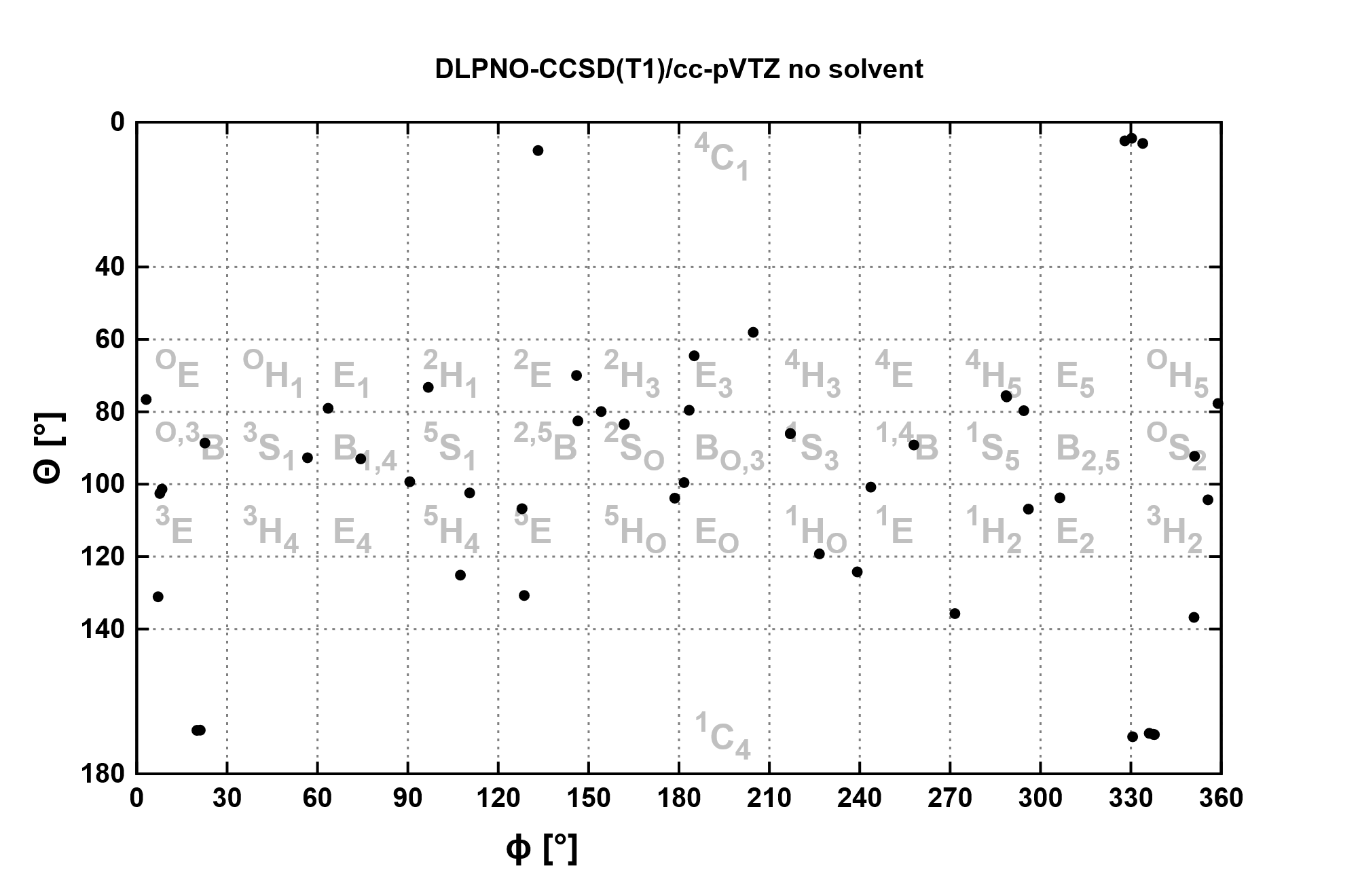


**Figure S13.** Final ensemble of 47 (CDCl_3_) / 50 (no solvent) lowest energy structures for which DLPNO-CCSD(T1)/cc-pVTZ energies have been evaluated.

In order to illustrate this in more detail, Figure S14 depicts the final ensemble of structures optimized at the B3LYP level of theory and the preceding structures optimized at lower levels of theory. This way, one can observe the changes upon increasing the level of theory from XTB to M06-2x-D3/svp to B3LYP-D4/tzvp and also compare the B3LYP results with and without CPCM. Here, clear differences upon refinement are visible as well as the general trend that higher levels of theory (refined functionals and corrections as well as larger basis sets) seem to deepen local minima effectively, reducing the scatter of the resulting structures. Furthermore, comparing results without and with CPCM (roughly speaking gas-phase vs. CDCl_3_) also seems to indicate a different bias. If solvent models are applied although the results do not change dramatically, which is probably because of screening of long-range electrostatic effects when using implicit solvation.


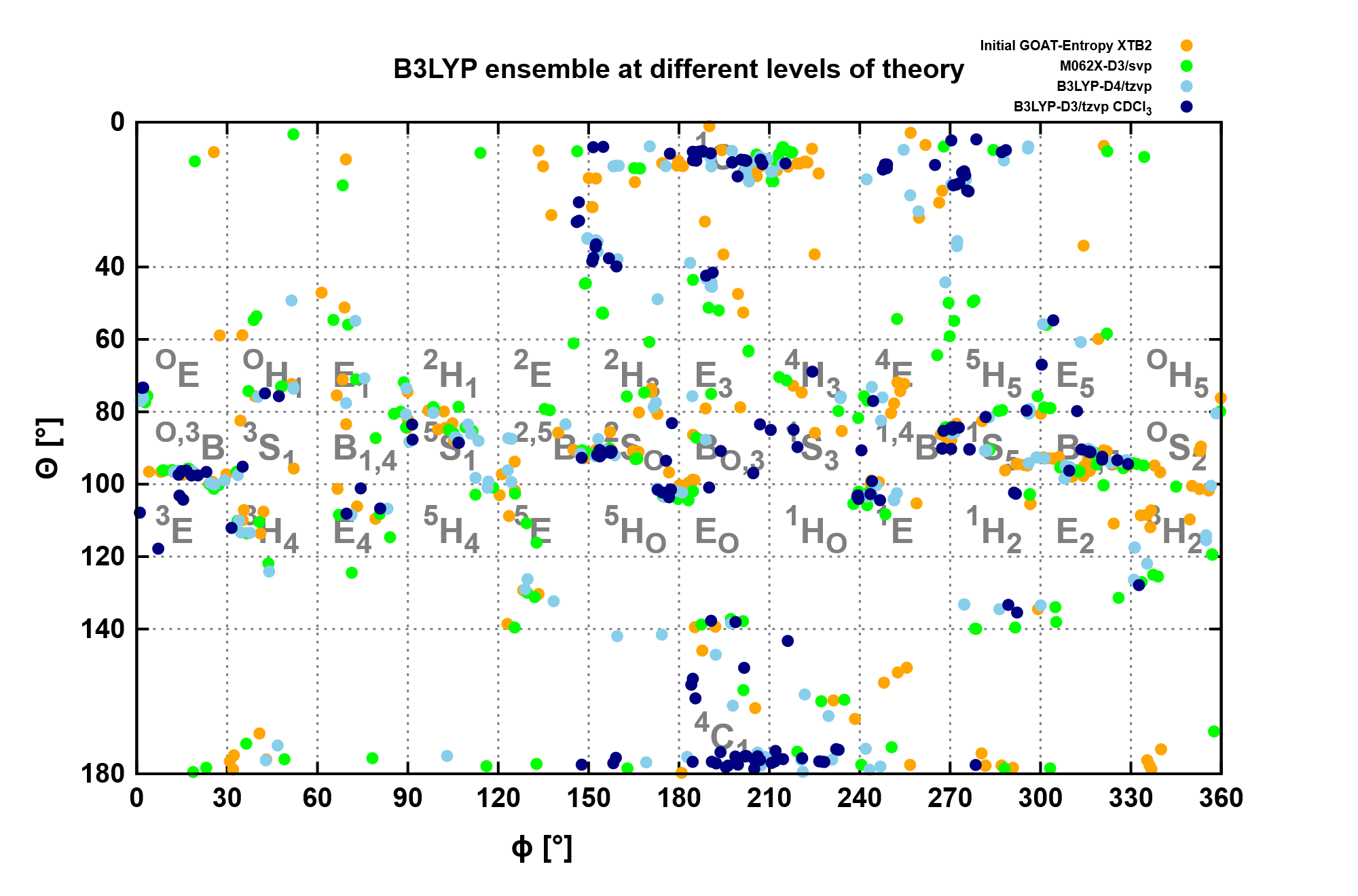
**Figure S14.** Mercator plot of the puckering angles of the ensemble of **3**. All structures of the final B3LYP (no solvent) ensemble are shown together with their preceding lower-level geometries (M06-2x and XTB) together with the B3LYP CPCM(CDCl_3_) ensemble.

## Example - transition state search for structures connecting different minima

The method outlined for the exploration of the conformational landscape yields all stable puckering conformers for a given species. For the distribution of conformers it is, however, also important to obtain information how the different conformers interconvert. In principle, it is possible to select all pairs of conformers adjacent in the mercator plot and perform transition state searches to map out (at least the majority) of possible paths between the conformers. While doing this comes at a significant price in terms of computational time, it can also be performed for certain interconversion of interest.

Here, we have carried out a transition state search for α-altropyranosyl trichloroacetimidate **2**, as for this system, the fit to the NMR spectroscopic data suggests an 80:20 mixture of ^4^*C*_1_ and *E*_3_. Given the fact that the *E*_3_ conformer is fairly high in energy (ΔG of more than + 40 kJ/mol) but close in puckering angles, the hypothesis is justified that a transition between these species is possible and would be observable if one assumes the relative energies might be overestimated.

Figure S15 displays the initial NEB path and the initial, final and optimized transition state structures between ^4^*C*_1_ and *E*_3_ in the mercator plot. The relative energies of the TS and product with respect to the ^4^*C*_1_ minimum are (B3LYP-D4/def2-tzvp CPCM(CDCl_3_) ) ΔE^*^ = 41.5 kJ/mol, ΔE = 38.5 kJ/mol.


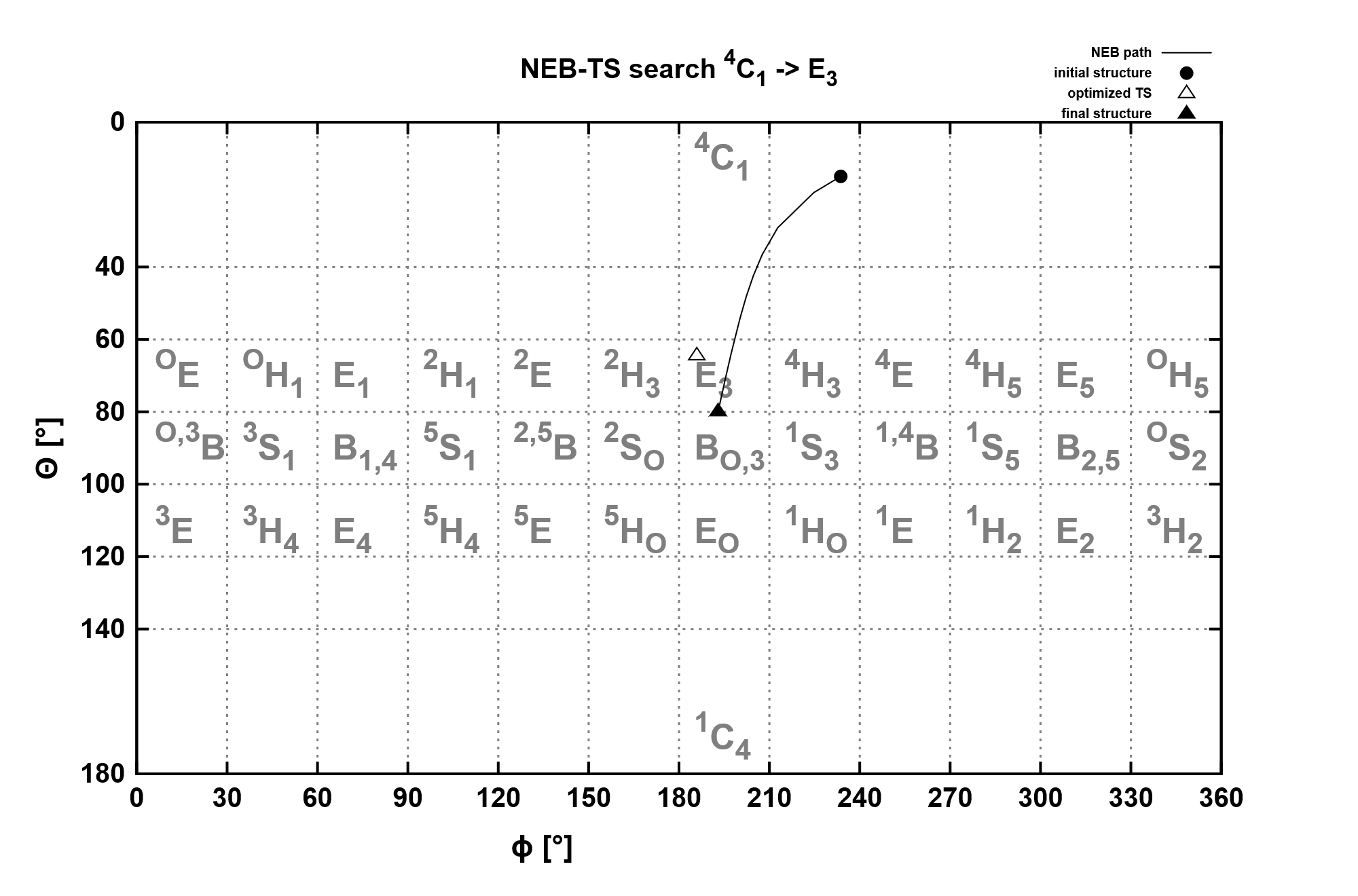


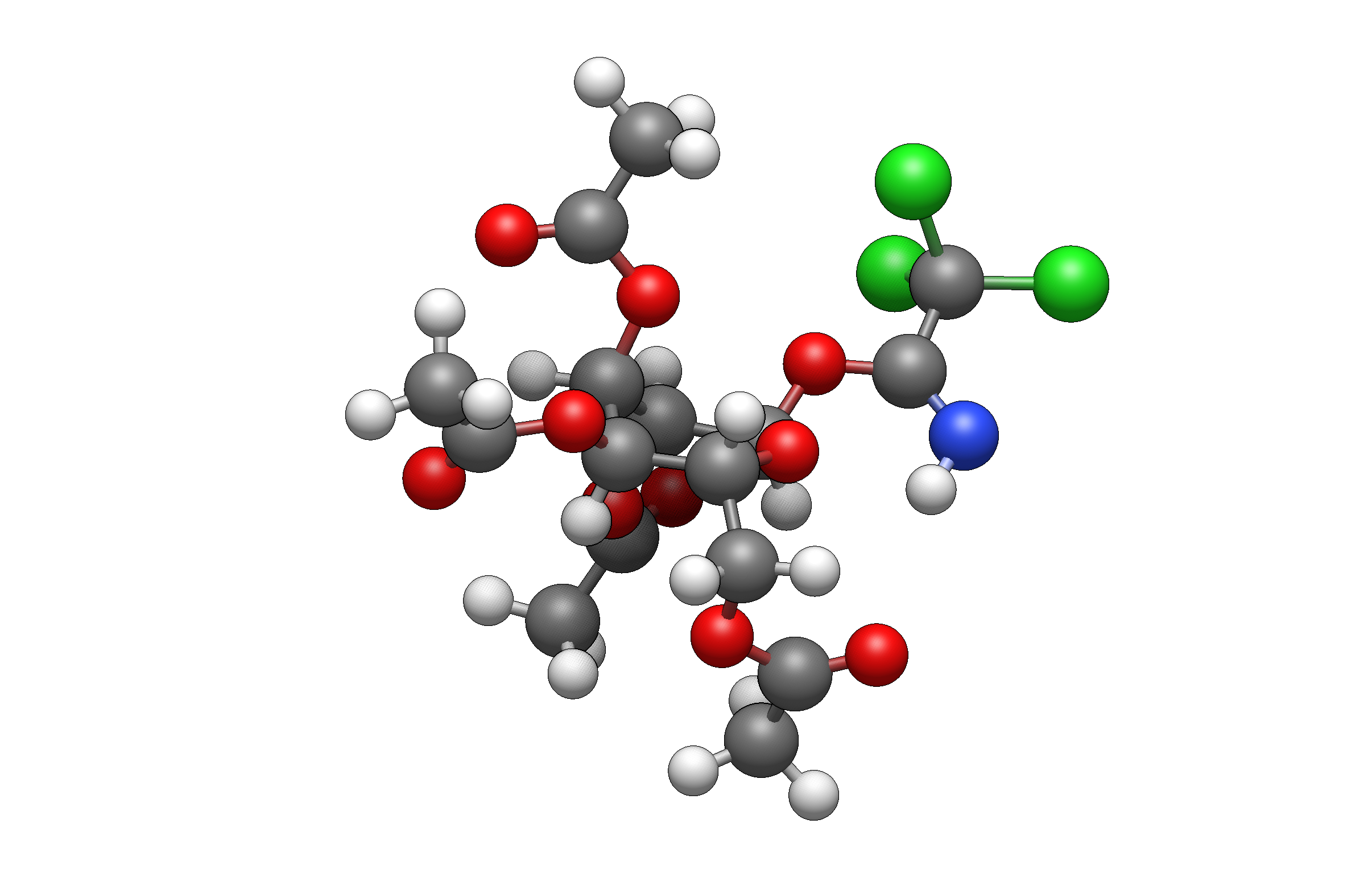


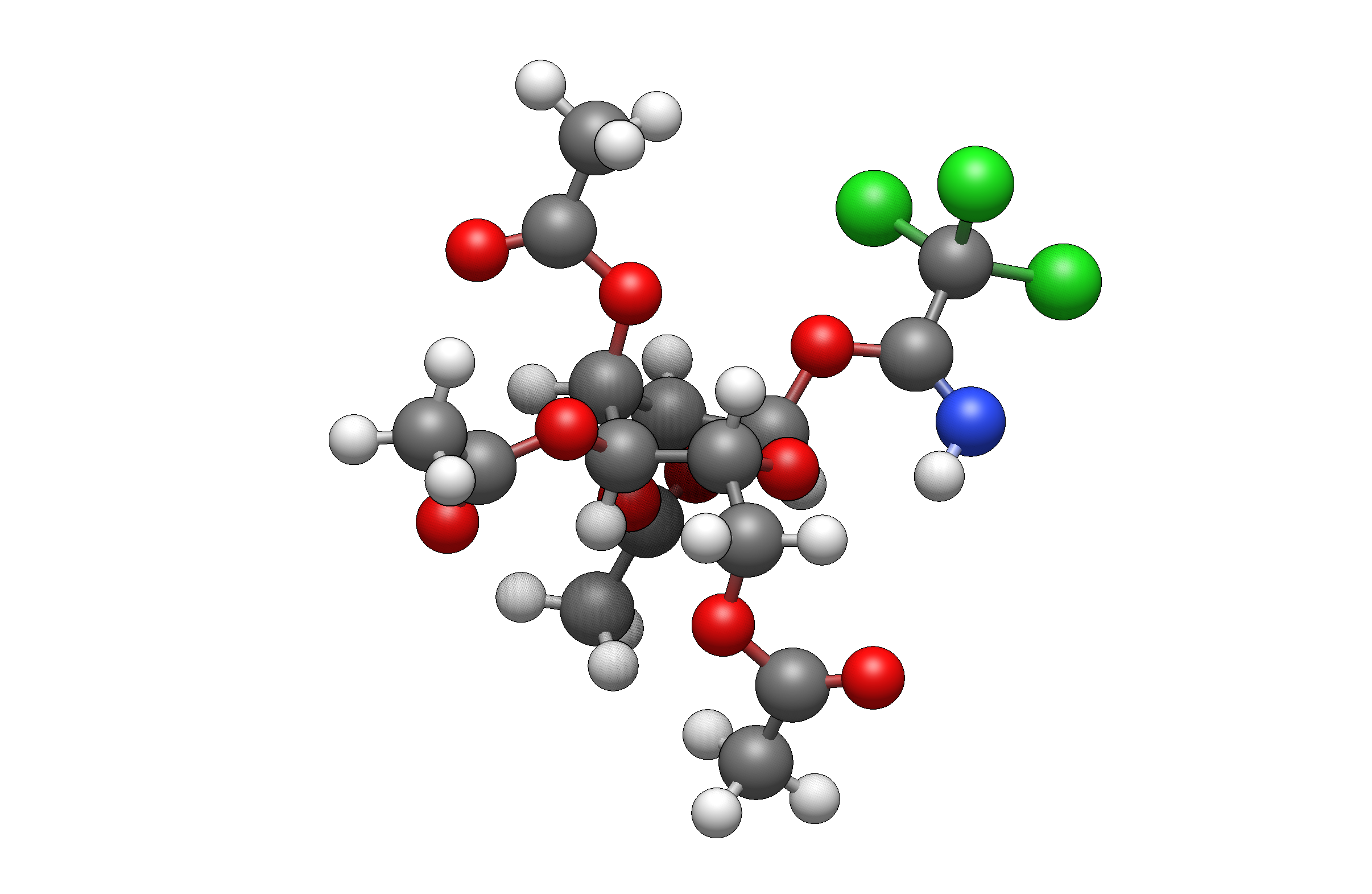


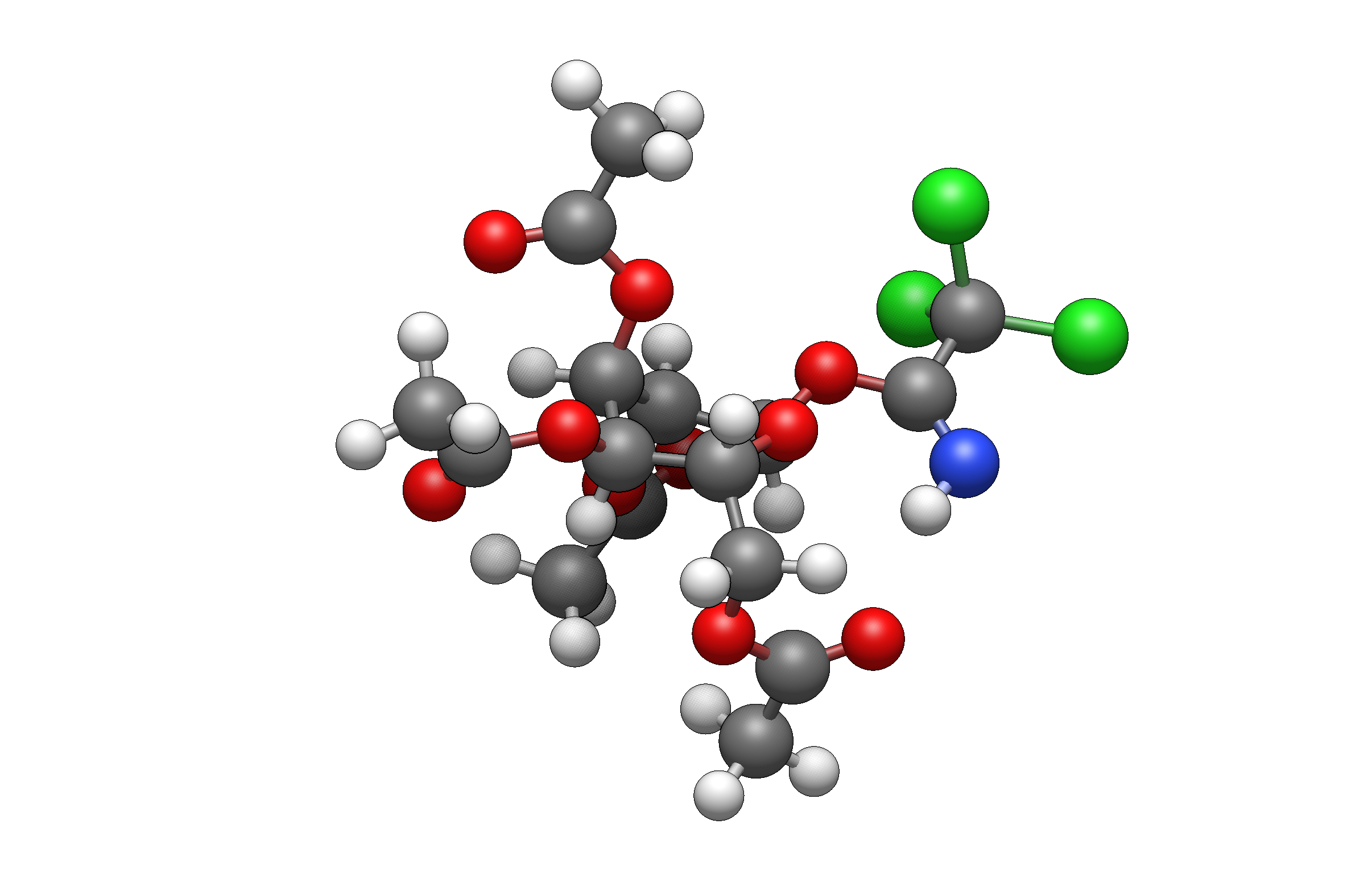


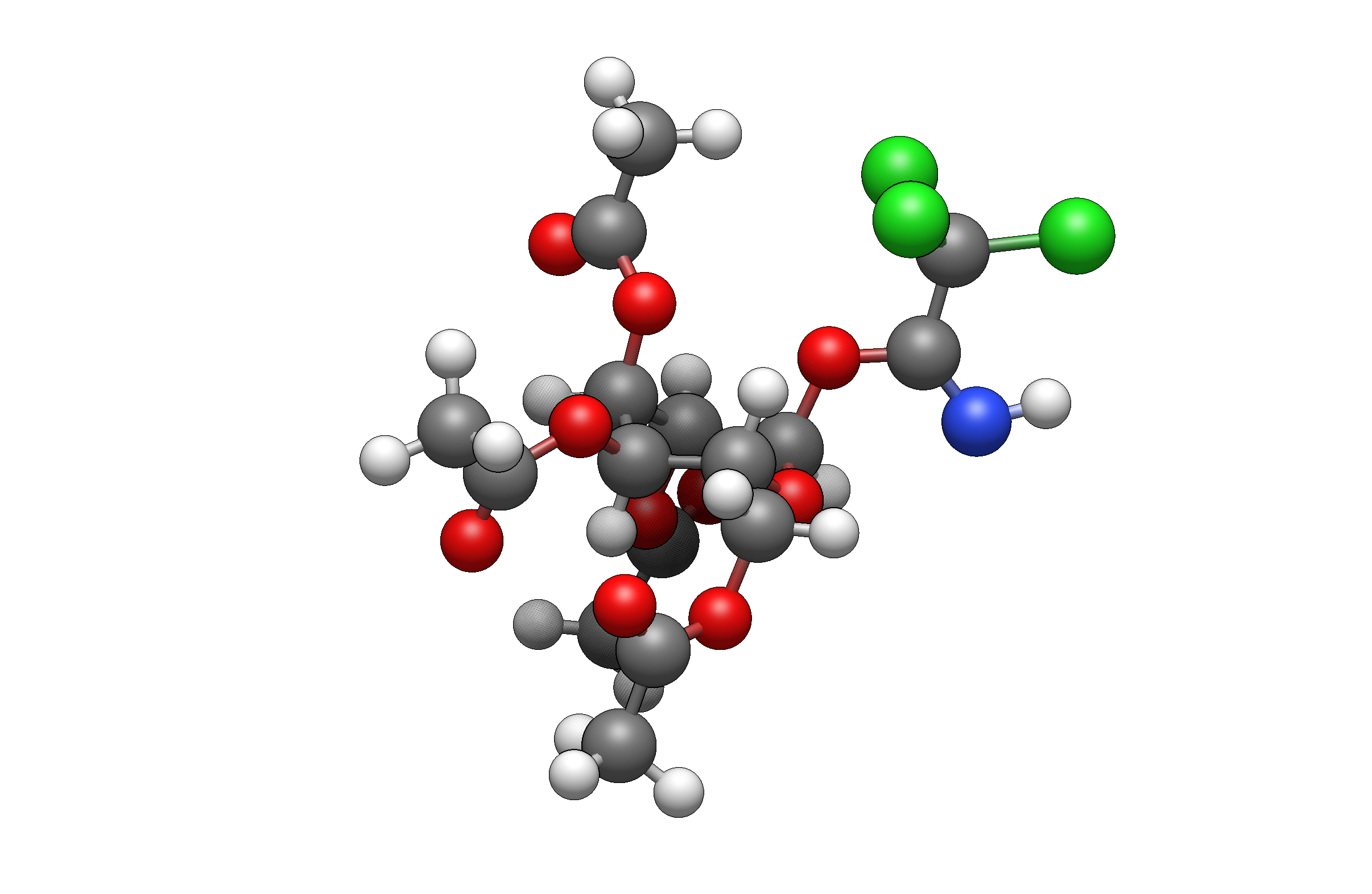


**Figure S15.** Results of the NEB-transition state search for α-altropyranosyl trichloroacetimidate **2** obtained at the B3LYP-D4/def2-tzvp CPCM(CDCl_3_) level of theory.

## Least squares fit procedure and error estimate for conformational equilibria

In order to arrive at a fit of the selected coupling constants obtained from electronic structure theory calculations to the measured values two selections are important. The first one is which coupling constants to choose for the fit. Here, our choice of four coupling constants that are very characteristic for the systems’ conformation has been outlined in a previous publication and its effectiveness has been demonstrated for a different set of compounds.^[6]^ The next choice is which computed compounds are used to fit the experimental values. As the hierarchical refinement procedure discussed here includes a very large number of possible conformers, overfitting can be a problem. Hence, we have adopted the following procedure: in a first step, a selection guided by the relative energy is carried out and only the lowest energy structures obtained at the highest level of theory are regarded for each puckering conformer. For this ensemble of typically 10-15 conformers, all structures and their corresponding couplings are fitted to the experiment. In every case, this has led to at most 4 conformers that contribute more than 1% to the overall fit. After this, the selection of all conformers that have contributions of more than 2% in the first fit are again used to obtain the final fit to experiment that are reported in the main paper. In all cases, decreasing the ensemble size never changed the distribution percentages by more than 5% and the RMSD by more than 0.2. Sensitivity tests in which the computed results of the most abundant conformer were altered by 1 Hz show that the final interpolated values will change roughly proportional, the percentages, however, can be more sensitive and changes of 10-15% have been observed. Hence, we assume that the interpolated couplings are accurate to 1 Hz, while the percentages may vary by +/- 5%.

For the LSQ fit procedure, a python script has been used that invokes the Sequential Least Squares Programming (SLSQP) algorithm scipy.optimize from the SciPy package.

As an example, we consider the fit reported in Table S1 for altroside **1** in D_2_O. Here, the hierarchical refinement procedure yields 22 structures, from which the lowest energy structures of each conformer are chosen, resulting in 13 sets of coupling constants, which are fitted to the experimental values. For this first fit, the following results are obtained:

**Table S1.** Details of the least squares fit of the coupling constants for compound **1** in D_2_O.

| LSQ weight | compound  number | corresponding  percentage | conformer |  |
| --- | --- | --- | --- | --- |
| **all minimum structures** | | | | |
| 0.8365 | 13 | 84.00% | ^4^*C*_1_ |  |
| 0.0000 | 33 |  |  |  |
| 0.0000 | 43 |  |  |  |
| 0.0696 | 59 | 7.00% | ^2^*S*_O_ |  |
| 0.0000 | 68 |  |  |  |
| 0.0715 | 124 | 7.00% | ^1^*C*_4_ |  |
| 0.0000 | 128 |  |  |  |
| 0.0000 | 134 |  |  |  |
| 0.0224 | 136 | 2.00% | ^O^*S*_2_ |  |
| 0.0000 | 166 |  |  |  |
| 0.0000 | 205 |  |  |  |
| 0.0000 | 264 |  |  |  |
| 0.0000 | 274 |  |  |  |
| experimental | 1.9 | 4.3 | 3.5 | 8.9 |
| interpolation | 1.9 | 4.2 | 3.9 | 8.9 |
| RMSD: | 0.214 |  |  |  |
| **reduced set of structures** | | | | |
| 0.8566 | 13 | 86.00% | ^4^*C*_1_ |  |
| 0.0631 | 59 | 6.00% | ^2^*S*_O_ |  |
| 0.0804 | 124 | 8.00% | ^1^*C*_4_ |  |
| experimental | 1.9 | 4.3 | 3.5 | 8.9 |
| interpolation | 1.9 | 4.3 | 3.9 | 8.9 |
| RMSD: | 0.221 |  |  |  |

(Note that conformer numbers are only for internal reference and correspond to initial numbering after applying GOAT)

Below, the results of the two stages of the fits are reported together with the fitted values and the RMSD for the three altrosides **1**-**3** and the corresponding solvents (Table S2-S5). The first dataset is the fit to all lowest energy structures, the second block is the final fit for the major components reported in the manuscript.

**Table S2.** Details of the least squares fit of the coupling constants for compound **1** in methanol-d_4._

| LSQ weight | compound  number | corresponding  percentage | conformer |  |  |  |
| --- | --- | --- | --- | --- | --- | --- |
| 0.8835 | 13 | 88.00% | ^4^*C*_1_ |  |  |  |
| 0.0000 | 33 |  |  |  |  |  |
| 0.0000 | 43 |  |  |  |  |  |
| 0.0643 | 59 | 6.00% | ^2^*S*_O_ |  |  |  |
| 0.0000 | 68 |  |  |  |  |  |
| 0.0085 | 124 | 1.00% | ^1^*C*_4_ |  |  |  |
| 0.0000 | 128 |  |  |  |  |  |
| 0.0000 | 134 |  |  |  |  |  |
| 0.0436 | 136 | 4.00% | ^O^*S*_2_ |  |  |  |
| 0.0000 | 166 |  |  |  |  |  |
| 0.0000 | 205 |  |  |  |  |  |
| 0.0000 | 264 |  |  |  |  |  |
| 0.0000 | 274 |  |  |  |  |  |
| experimental: | 1.6 | 3.9 | 3.3 | 9.5 |  |  |
| interpolation: | 1.7 | 3.8 | 3.9 | 9.4 | RMSD: | 0.31 |
| 0.8780 | 13 | ^4^*C*_1_ | 88.00% |  |  |  |
| 0.0716 | 59 | ^2^*S*_O_ | 7.00% |  |  |  |
| 0.0504 | 136 | ^O^*S*_2_ | 5.00% |  |  |  |
| experimental: | 1.6 | 3.9 | 3.3 | 9.5 |  |  |
| interpolation: | 1.6 | 3.7 | 3.9 | 9.4 | RMSD: | 0.312 |

(Note that conformer numbers are only for internal reference and correspond to initial numbering after applying GOAT)

**Table S3.** Details of the least squares fit of the coupling constants for compound **1** in DMSO-d_6_.

| LSQ weight | compound  number | corresponding  percentage | conformer |  |  |  |
| --- | --- | --- | --- | --- | --- | --- |
| 0.8243 | 13 | 82.00% | ^4^*C*_1_ |  |  |  |
| 0.0000 | 33 |  |  |  |  |  |
| 0.0000 | 43 |  |  |  |  |  |
| 0.0771 | 59 | 8.00% | ^2^*S*_O_ |  |  |  |
| 0.0000 | 68 |  |  |  |  |  |
| 0.0591 | 124 | 6.00% | ^1^*C*_4_ |  |  |  |
| 0.0000 | 128 |  |  |  |  |  |
| 0.0000 | 134 |  |  |  |  |  |
| 0.0395 | 136 | 4.00% | ^O^*S*_2_ |  |  |  |
| 0.0000 | 166 |  |  |  |  |  |
| 0.0000 | 205 |  |  |  |  |  |
| 0.0000 | 264 |  |  |  |  |  |
| 0.0000 | 274 |  |  |  |  |  |
| experimental: | 1.9 | 4.2 | 3.5 | 8.9 |  |  |
| interpolation: | 1.9 | 4.1 | 3.9 | 8.9 | RMSD: | 0.206 |
| 0.8242 | 82.00% | 13 | ^4^*C*_1_ |  |  |  |
| 0.0771 | 8.00% | 59 | ^2^*S*_O_ |  |  |  |
| 0.0591 | 6.00% | 124 | ^1^*C*_4_ |  |  |  |
| 0.0396 | 4.00% | 136 | ^O^*S*_2_ |  |  |  |
| experimental: | 1.9 | 4.2 | 3.5 | 8.9 |  |  |
| interpolation: | 1.9 | 4.1 | 3.9 | 8.9 | RMSD: | 0.206 |

(Note that conformer numbers are only for internal reference and correspond to initial numbering after applying GOAT)

**Table S4.** Details of the least squares fit of the coupling constants for compound **2** in CDCl_3_.

| LSQ weight | compound  number | corresponding  percentage | conformer |  |  |  |
| --- | --- | --- | --- | --- | --- | --- |
| 0.0000 | 4594 |  |  |  |  |  |
| 0.0000 | 3213 |  |  |  |  |  |
| 0.8385 | 25 | 83.00% | ^4^*C*_1_ |  |  |  |
| 0.0000 | 377 |  |  |  |  |  |
| 0.0000 | 594 |  |  |  |  |  |
| 0.0000 | 617 |  |  |  |  |  |
| 0.0000 | 747 |  |  |  |  |  |
| 0.0000 | 959 |  |  |  |  |  |
| 0.0102 | 1004 | 1.00% | ^O,3^*B* |  |  |  |
| 0.0000 | 1217 |  |  |  |  |  |
| 0.0000 | 1222 |  |  |  |  |  |
| 0.0061 | 1372 | 1.00% | ^2^*S*_O_ |  |  |  |
| 0.0000 | 1619 |  |  |  |  |  |
| 0.1453 | 1719 | 15.00% | ^1^*H*_2_ |  |  |  |
| 0.0000 | 1753 |  |  |  |  |  |
| 0.0000 | 1977 |  |  |  |  |  |
| 0.0000 | 2364 |  |  |  |  |  |
| 0.0000 | 2491 |  |  |  |  |  |
| experimental: | 1 | 3.4 | 3.3 | 10.2 |  |  |
| interpolation: | 1.2 | 3.4 | 3.4 | 10.2 | RMSD: | 0.123 |
| 0.8017 | 80.00% | ^4^*C*_1_ |  |  |  |  |
| 0.1983 | 20.00% | ^1^*H*_2_ |  |  |  |  |
| experimental: | 1 | 3.4 | 3.3 | 10.2 |  |  |
| interpolation: | 1.2 | 3.4 | 3.4 | 10.2 | RMSD: | 0.128 |

(Note that conformer numbers are only for internal reference and correspond to initial numbering after applying GOAT)

**Table S5.** Details of the least squares fit of the coupling constants for compound **3** in CDCl_3_.

|  |  |  |  |  |  |  |
| --- | --- | --- | --- | --- | --- | --- |
| LSQ weight | compound  number | corresponding  percentage | conformer |  |  |  |
| 0.0000 | 73 |  |  |  |  |  |
| 0.5128 | 81 | 51.00% | ^1^*C*_4_ |  |  |  |
| 0.0000 | 249 |  |  |  |  |  |
| 0.0000 | 578 |  |  |  |  |  |
| 0.4213 | 678 | 42.00% | ^4^*C*_1_ |  |  |  |
| 0.0000 | 965 |  |  |  |  |  |
| 0.0000 | 991 |  |  |  |  |  |
| 0.0000 | 1093 |  |  |  |  |  |
| 0.0000 | 1505 |  |  |  |  |  |
| 0.0660 | 1567 | 7.00% | ^2,5^*B* |  |  |  |
| 0.0000 | 1610 |  |  |  |  |  |
| 0.0000 | 1618 |  |  |  |  |  |
| 0.0000 | 1629 |  |  |  |  |  |
| 0.0000 | 1755 |  |  |  |  |  |
| 0.0000 | 1793 |  |  |  |  |  |
| 0.0000 | 1815 |  |  |  |  |  |
| 0.0000 | 1909 |  |  |  |  |  |
| 0.0000 | 2070 |  |  |  |  |  |
| 0.0000 | 2695 |  |  |  |  |  |
| 0.0000 | 2749 |  |  |  |  |  |
| 0.0000 | 2831 |  |  |  |  |  |
| 0.0000 | 3043 |  |  |  |  |  |
| 0.0000 | 3472 |  |  |  |  |  |
| 0.0000 | 3632 |  |  |  |  |  |
| 0.0000 | 3671 |  |  |  |  |  |
| 0.0000 | 4556 |  |  |  |  |  |
| experimental: | 2.8 | 8.1 | 3.3 | 5 |  |  |
| interpolation: | 3.1 | 8.1 | 3.7 | 5.1 | RMSD: | 0.277 |
| 0.5128 | 81 | ^1^*C*_4_ | 51.00% |  |  |  |
| 0.4213 | 678 | ^4^*C*_1_ | 42.00% |  |  |  |
| 0.0660 | 1567 | ^2,5^*B* | 7.00% |  |  |  |
| experimental: | 2.8 | 8.1 | 3.3 | 5 |  |  |
| interpolation: | 3.1 | 8.1 | 3.7 | 5.1 | RMSD: | 0.277 |

(Note that conformer numbers are only for internal reference and correspond to initial numbering after applying GOAT)

## File attachment: Molecular structures and energies

Relative energies (see attached spreadsheet) and molecular structures (see attached zip archive) of all molecular species obtained at the B3LYP-D4/def2-TVZP level of theory. These are the basis for DLPNO-CCSD(T1)/cc-pVTZ energy calculations and PBE0/pcJ-3 spin-spin coupling constant calculations^[7]^ outlined in the main manuscript. *Note that all obtained geometries and further information on properties and energies are available in the form of a compressed archive online/ upon request.*

All calculations have been carried out using a development version of the ORCA 6.0 program package.^[1]^ The corresponding input files for all steps of the hierarchy of the ensemble optimization procedure and the successive property calculations are sketched below :

Step 1: GOAT-Entropy conformational search:

!XTB2 GOAT-ENTROPY PAL16

%maxcore 2000

*xyzfile 0 1 altrose.xyz

Step 2: M06-2x-D3/def2-svp optimization with optional solvent

!M062X D3zero def2-svp def2/J tightopt defgrid3 CPCM(methanol)

*xyzfile 0 1 conformer009_Compound_1.xyz

Step 3: B3LYP-D3/def2-tzvp optimization and frequency calculation (optional CPCM implicit solvation treatement)

!B3LYP D4 def2-tzvp def2/J def2/JK tightopt freq CPCM(methanol)

*xyzfile 0 1 conformer128_Compound_1_Compound_1.xyz

Step 4: DLPNO-CCSD(T1)/cc-pVTZ energy calculations (optional CPCM)

!DLPNO-CCSD(T1) cc-pVTZ cc-pVTZ/C CPCM(DMSO) TIGHTSCF PAL4

%maxcore 8000

* xyzfile 0 1 conformer004_Compound_1.xyz

Step 5: PBE/pcJ-3 spin-spin coupling calculation

!PBE pcJ-3 autoaux tightscf CPCM(DMSO) PAL4

%maxcore 4000

* xyzfile 0 1 conformer004_Compound_1.xyz

%eprnmr

Nuclei = all C { ssall, ist = 13 }

Nuclei = all H { ssall }

end

Step 6: MP2/aug-cc-pVTZ^[8]^ dipole moments for selected structures

!MP2 aug-cc-pVTZ aug-cc-pVTZ/C tightscf PAL4

%maxcore 7000

%mp2 density relaxed end

%elprop dipole true

end

* xyzfile 0 1 conformer000_Compound_1.xyz

# Microwave spectroscopy of methyl α-d-altropyranoside (1)

## Experimental method – Chirped pulse Fourier transform microwave spectroscopy

The sample of methyl α-d-altropyranoside (**1**), a yellowish-coloured syrup, was synthetized according to the procedure described by Lütjohann et al.^[8]^ Its rotational spectrum was recorded using the chirped-pulse Fourier transform microwave spectrometer COMPACT in Hamburg, operating in the 2-18 GHz frequency range (for details see publications of Schmitz et al.^[10]^ and Pérez et al.^[11]^).

To achieve sufficient gas-phase concentration of the sample, approximately 400 mg of sample was placed in a custom-built reservoir and heated to around 175 °C using a conventional heating system. The vaporized sample was then introduced into the vacuum chamber through supersonic expansion. This was done by positioning the reservoir directly at the orifice of a pulsed valve and flowing neon gas, at a backing pressure of 2.5 bar, over the reservoir to carry the vaporized molecules. The created mixture was then adiabatically expanded into the vacuum chamber.

The rotational spectrum of methyl α-d-altropyranoside was recorded by probing each gas pulse with a train of eight chirped microwave pulses, each with a duration of 4 μs and covering the 2-8 GHz region of the electromagnetic spectrum. These pulses were generated using an arbitrary waveform generator. Before interacting with the gas pulse, each chirped microwave pulse is amplified by using a 300 W traveling wave tube amplifier and transmitted perpendicularly to the propagation of the jet expansion into the vacuum chamber using a horn antenna. Whenever a frequency within the excitation chirped pulse was resonant with the difference in energy between two rotational levels of the investigated molecule, a coherent superposition was created. The decay of this coherent superposition was detected by a second horn antenna positioned opposite the transmitting horn antenna. The signal was then subsequently amplified with a low-noise microwave amplifier and recorded for 40 μs as free-induction decay (FID) using a fast oscilloscope. Finally, the rotational spectrum in the frequency domain was obtained by fast Fourier transformation of the FID. The rotational spectrum of methyl α-D-altropyranoside was measured at an operating repetition rate of 8 Hz, which resulted in an effective repetition rate of 64 Hz, given that each gas pulse was probed with eight microwave chirped pulses. The final rotational spectrum was obtained by averaging together 4.5⸱10^6^ FIDs.

The rotational spectrum was fitted using the *A*-reduction of the Watson’s Hamiltonian in the *I*^r^ representation, as implemented in the SPFIT/SPCAT program.^[12]^

## Theoretical results

**Table S6.** Rotational constants (A, B, and C in MHz) and electronic dipole moment components (μ_a_, μ_b_, and μ_c_ in Debye) for the nine low-lying energy conformers of methyl α-D-altropyranoside calculated at the B3LYP-D4/def2-TZVP level of theory. The table also includes relative electronic energies (ΔE_0_ in kJ/mol) with vibrational zero-point energy corrections calculated at the B3LYP-D4/def2-TZVP level of theory and the relative Gibbs energies (ΔG in kJ/mol). The relative Gibbs energies were obtained by combining the DLPNO-CCSD(T1) energies with free energy corrections calculated at the B3LYP-D4/def2-TZVP level of theory.

| Conformer | ΔE_0_ | ΔG | *A* | *B* | *C* | *μ_a_* | *μ_b_* | *μ_b_* |
| --- | --- | --- | --- | --- | --- | --- | --- | --- |
| G^+^g^-^\cc\G^+^ | 0.0 | 0.0 | 867.4 | 755.3 | 581.6 | 0.4 | 3.1 | -0.3 |
| G^-^g^+^\cc\G^+^ | 0.4 | 0.3 | 911.2 | 715.0 | 612.4 | -2.8 | -3.3 | 1.1 |
| Tg^+^\cc\G^+^ | 0.3 | 1.5 | 971.8 | 684.2 | 570.1 | -2.6 | 1.8 | 0.1 |
| G^-^g^+^\cc\T | 3.6 | 3.5 | 916.3 | 713.0 | 608.5 | -3.9 | -1.0 | 0.1 |
| G^+^g^-^\cc\T | 4.1 | 3.8 | 869.0 | 758.0 | 575.3 | 2.7 | 2.1 | -0.4 |
| G^+^g^-^\cc\G^-^ | 4.6 | 4.4 | 868.1 | 757.5 | 575.4 | 2.1 | 4.0 | -1.1 |
| G^-^g^+^\cc\G^-^ | 5.5 | 5.4 | 914.0 | 714.7 | 607.3 | -4.7 | -2.9 | -0.4 |
| Tg^+^\cc\T | 4.5 | 5.4 | 978.4 | 681.5 | 562.2 | -3.8 | -0.5 | 0.3 |
| Tg^+^\cc\G^-^ | 4.8 | 5.7 | 976.2 | 683.8 | 563.3 | -4.5 | 1.4 | -0.5 |


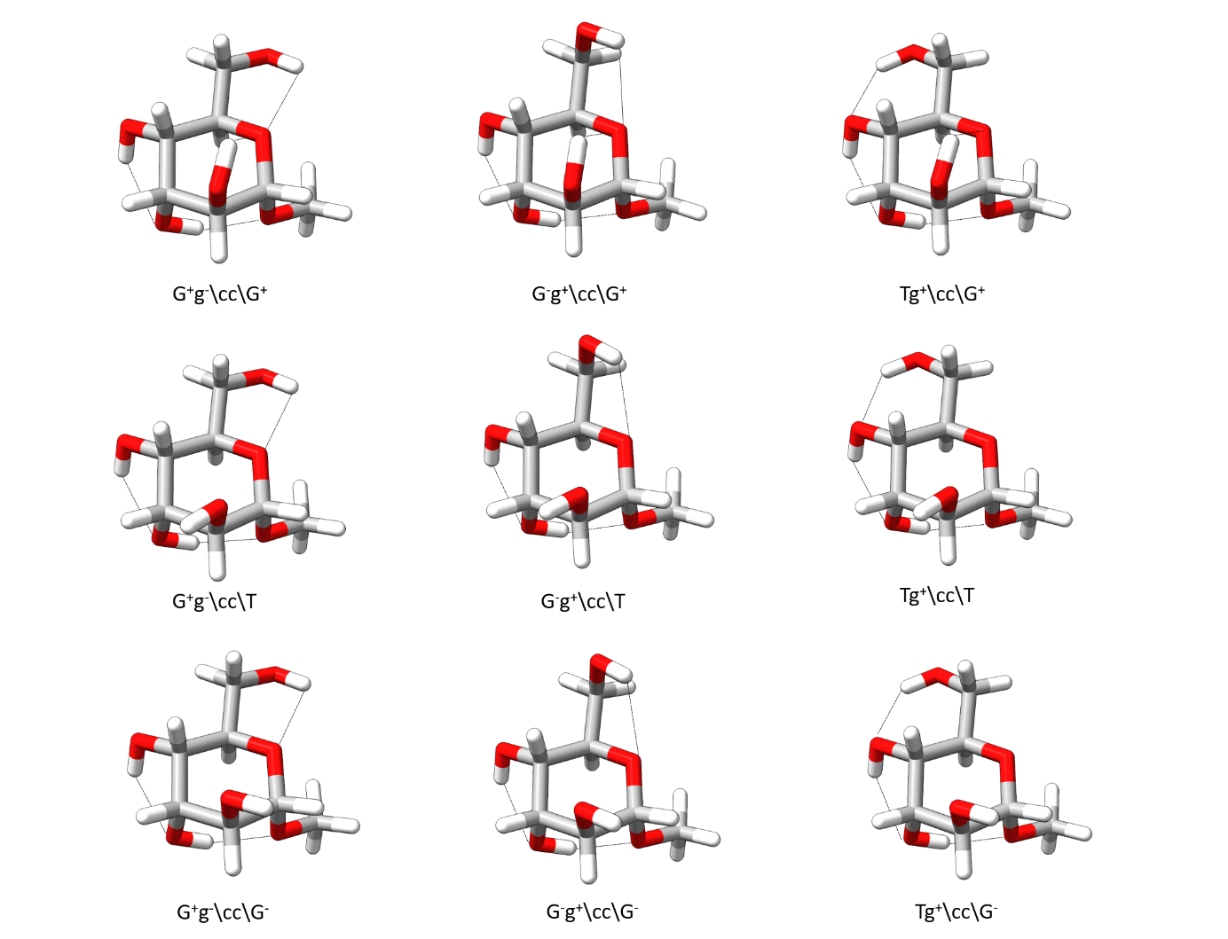


**Figure S16.** Theoretical structures of the nine energetically low-lying conformers of methyl α-d-altropyranoside (**1**) within an energy window of 10 kJ/mol calculated at the B3LYP-D4/def2-TZVP level of theory.

### Methyl internal rotation


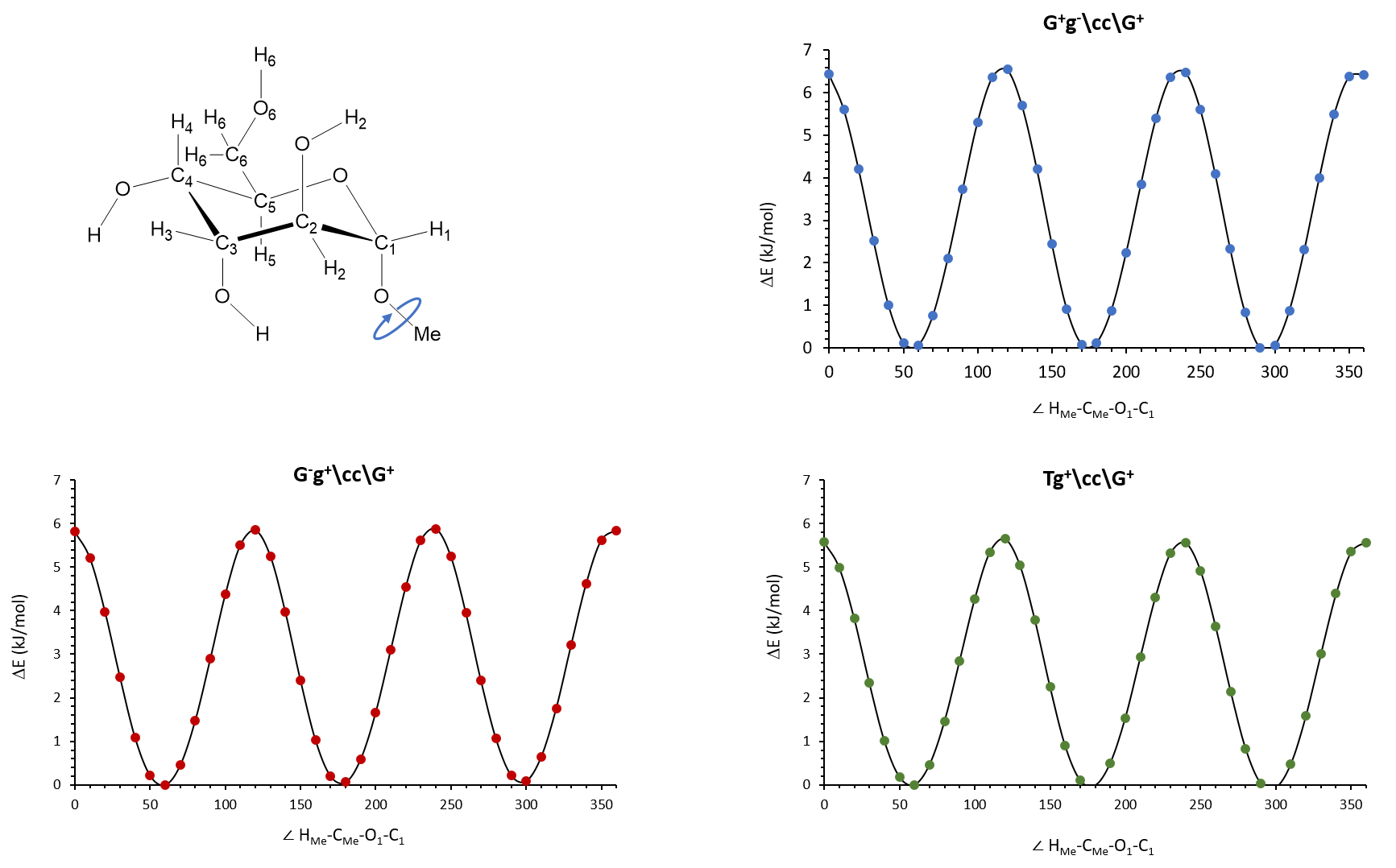
The structure of methyl α-d-altropyranoside features one methyl group belonging to the methoxy group (MeO) bonded to the anomeric carbon (C_1_). The rotation of this methyl group results in three equivalent rotamers. The coupling between the methyl internal rotation and the overall rotation of the molecule could, depending on the height of the barrier hindering methyl internal rotation, split each rotational transition into two components, E and A. To investigate the barriers to methyl internal rotation in the three observed conformers of methyl α-d-altropyranoside, 360° relaxed scans were performed in steps of 10° along the relevant dihedral angle. The scans were performed at the B3LYP-D4/def2-TZVP level of theory. The calculated barriers for the methyl internal rotation of the three observed conformers range between 5.6 kJ/mol and 6.5 kJ/mol and are shown in Figure S17.

**Figure S17.** Methyl internal rotation barriers calculated at the B3LYP-D4/def2-TZVP level of theory for the three experimentally observed conformers of methyl α-d-altropyranoside: G^+^g^-^/cc/G^+^ (blue), G^-^g^+^/cc/G^+^ (red), and Tg^+^/cc/G^+^ (green).

### Conformational relaxation barriers


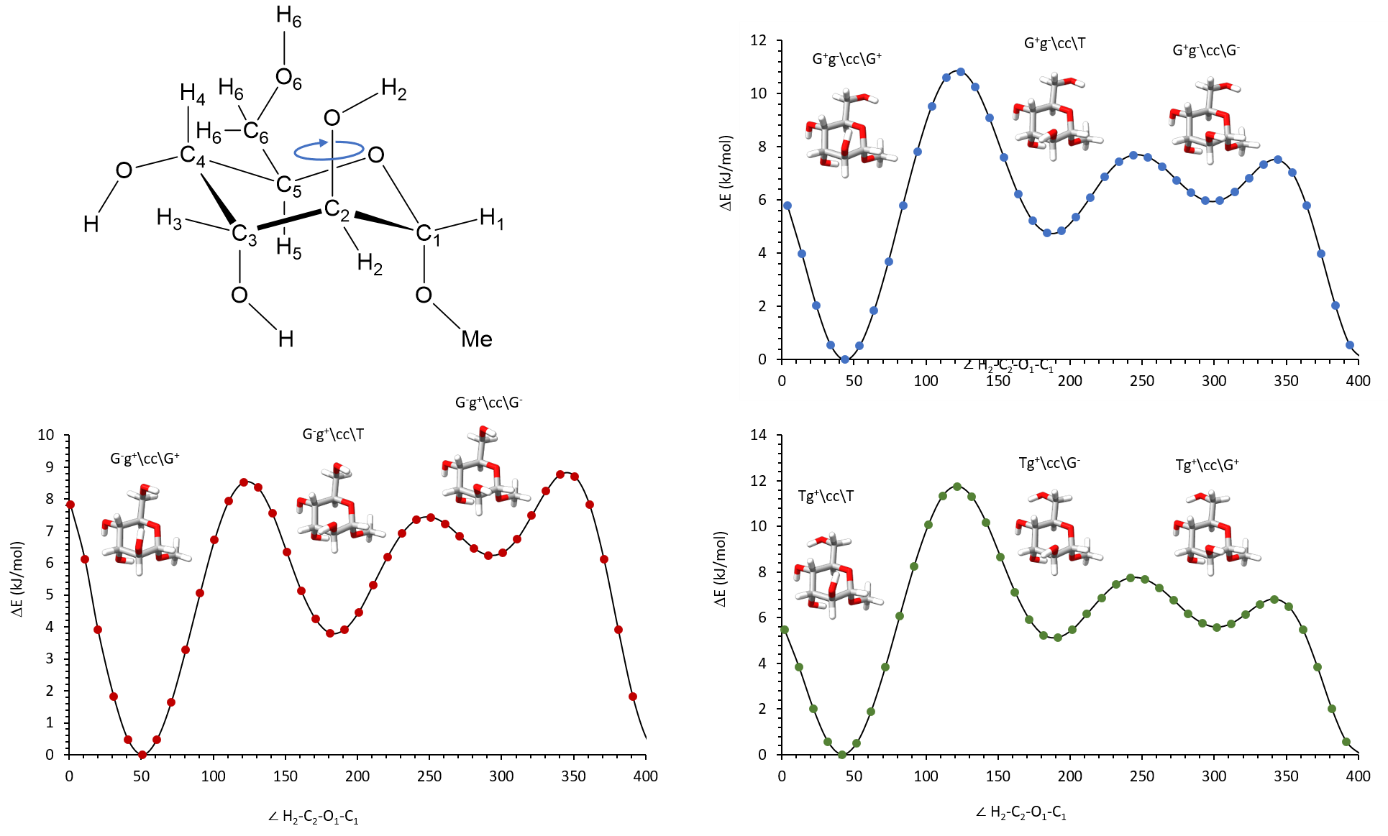
Exploration of the potential energy surface of methyl α-d-altropyranoside yielded nine conformers within an energy window of 10 kJ/mol. The nine conformers differ in the orientation of the -CH2OH group (G+g-, G-g+, G+T) and in the orientation of the 2-OH group (G+, G-, and T), as it can be seen in Figure S18. Experimentally we observed three conformers with different orientations of the CH_2_OH group (G+g-, G-g+, G+T) but an equivalent orientation of the 2-OH group (G+). To account for the absence of the other six conformers in the experimental data, we conducted relaxed potential energy scans for the rotation of the 2-OH group. These scans were initiated from the geometries of the three experimentally observed conformers and performed using the B3LYP-D4/def2-TZVP level of theory.

**Figure S18.** Conformational relaxation barriers for the 2-OH rotation calculated at the B3LYP-D4/def2-TZVP level of theory for the three experimentally observed conformers of methyl α-d-altropyranoside: G^+^g^-^/cc/G^+^ (blue), G^-^g^+^/cc/G^+^ (red), and Tg^+^/cc/G^+^ (green).

### Frequency list of the G^+^g^-^/cc/G^+^ conformer of methyl α-d-altropyranoside (1)

**Table S7.** Measured (_obs_) and calculated frequencies (_calc_) of the observed rotational transitions for the conformer G^+^g^-^\cc\G^+^, along with their residuals ()

| ***J’*** | ***K_a_*’** | ***K_c_’*** | ***J’’*** | ***K_a_’’*** | ***K_c_’’*** | ****_obs_/MHz** | ****_calc_/MHz** | **Δ**/MHz** |
| --- | --- | --- | --- | --- | --- | --- | --- | --- |
| 7 | 4 | 4 | 7 | 3 | 5 | 2009.7525 | 2009.7530 | -0.0005 |
| 9 | 4 | 5 | 9 | 3 | 6 | 2220.6333 | 2220.6379 | -0.0045 |
| 7 | 7 | 0 | 7 | 6 | 1 | 2278.6465 | 2278.6446 | 0.0018 |
| 7 | 7 | 1 | 7 | 6 | 2 | 2298.0456 | 2298.0354 | 0.0102 |
| 8 | 3 | 5 | 8 | 2 | 6 | 2357.1930 | 2357.1952 | -0.0021 |
| 8 | 4 | 5 | 8 | 3 | 6 | 2422.2481 | 2422.2358 | 0.0123 |
| 7 | 2 | 5 | 7 | 1 | 6 | 2432.7586 | 2432.7747 | -0.0160 |
| 2 | 0 | 2 | 1 | 1 | 1 | 2469.2753 | 2469.2761 | -0.0008 |
| 6 | 1 | 5 | 6 | 0 | 6 | 2472.4372 | 2472.4299 | 0.0072 |
| 2 | 1 | 2 | 1 | 0 | 1 | 2620.9309 | 2620.9344 | -0.0034 |
| 10 | 4 | 6 | 10 | 3 | 7 | 2766.5054 | 2766.5175 | -0.0120 |
| 9 | 4 | 6 | 9 | 3 | 7 | 2867.9795 | 2867.9890 | -0.0095 |
| 8 | 2 | 6 | 8 | 1 | 7 | 2899.0221 | 2899.0240 | -0.0018 |
| 8 | 3 | 6 | 8 | 2 | 7 | 2902.9618 | 2902.9637 | -0.0019 |
| 7 | 1 | 6 | 7 | 0 | 7 | 2928.9421 | 2928.9349 | 0.0071 |
| 7 | 2 | 6 | 7 | 1 | 7 | 2929.5410 | 2929.5371 | 0.0040 |
| 2 | 2 | 1 | 1 | 1 | 0 | 3200.9701 | 3200.9719 | -0.0017 |
| 9 | 2 | 7 | 9 | 1 | 8 | 3357.9919 | 3357.9977 | -0.0057 |
| 8 | 1 | 7 | 8 | 0 | 8 | 3383.8741 | 3383.8713 | 0.0027 |
| 2 | 2 | 0 | 1 | 1 | 1 | 3482.2580 | 3482.2589 | -0.0008 |
| 3 | 0 | 3 | 2 | 1 | 2 | 3699.6502 | 3699.6550 | -0.0047 |
| 3 | 1 | 3 | 2 | 0 | 2 | 3747.8105 | 3747.8115 | -0.0009 |
| 3 | 1 | 2 | 2 | 2 | 1 | 3856.7689 | 3856.7707 | -0.0017 |
| 3 | 2 | 2 | 2 | 2 | 1 | 4028.6191 | 4028.6198 | -0.0006 |
| 3 | 1 | 2 | 2 | 1 | 1 | 4194.5753 | 4194.5773 | -0.0019 |
| 3 | 2 | 2 | 2 | 1 | 1 | 4366.4219 | 4366.4264 | -0.0045 |
| 4 | 0 | 4 | 3 | 1 | 3 | 4883.8168 | 4883.8322 | -0.0153 |
| 4 | 1 | 4 | 3 | 1 | 3 | 4885.5648 | 4885.5739 | -0.0090 |
| 4 | 1 | 4 | 3 | 0 | 3 | 4894.6836 | 4894.6744 | 0.0092 |
| 3 | 3 | 1 | 2 | 2 | 0 | 4985.0346 | 4985.0413 | -0.0066 |
| 4 | 2 | 2 | 3 | 3 | 1 | 5078.3575 | 5078.3580 | -0.0004 |
| 3 | 3 | 0 | 2 | 2 | 1 | 5135.3412 | 5135.3396 | 0.0016 |
| 4 | 1 | 3 | 3 | 2 | 2 | 5224.7475 | 5224.7680 | -0.0205 |
| 4 | 2 | 3 | 3 | 2 | 2 | 5283.4773 | 5283.4836 | -0.0063 |
| 3 | 2 | 1 | 2 | 1 | 2 | 5292.4548 | 5292.4553 | -0.0004 |
| 4 | 2 | 3 | 3 | 1 | 2 | 5455.3344 | 5455.3328 | 0.0016 |
| 4 | 3 | 2 | 3 | 3 | 1 | 5502.5658 | 5502.5668 | -0.0009 |
| 9 | 2 | 8 | 8 | 3 | 5 | 5914.0863 | 5914.0972 | -0.0108 |
| 5 | 0 | 5 | 4 | 1 | 4 | 6053.3853 | 6053.3872 | -0.0018 |
| 5 | 1 | 5 | 4 | 0 | 4 | 6055.4316 | 6055.4292 | 0.0024 |
| 5 | 3 | 2 | 4 | 4 | 1 | 6151.3194 | 6151.3066 | 0.0128 |
| 4 | 3 | 2 | 3 | 2 | 1 | 6169.0706 | 6169.0796 | -0.0090 |
| 5 | 1 | 4 | 4 | 2 | 3 | 6475.8752 | 6475.8728 | 0.0024 |
| 5 | 2 | 4 | 4 | 2 | 3 | 6490.7443 | 6490.7408 | 0.0035 |
| 6 | 3 | 4 | 5 | 4 | 1 | 6516.4346 | 6516.4312 | 0.0033 |
| 5 | 1 | 4 | 4 | 1 | 3 | 6534.5950 | 6534.5884 | 0.0066 |
| 5 | 2 | 3 | 4 | 3 | 2 | 6618.3098 | 6618.3060 | 0.0037 |
| 6 | 4 | 3 | 5 | 5 | 0 | 6689.6222 | 6689.6187 | 0.0034 |
| 4 | 4 | 1 | 3 | 3 | 0 | 6768.4933 | 6768.4963 | -0.0030 |
| 4 | 3 | 1 | 3 | 2 | 2 | 6807.9060 | 6807.9096 | -0.0035 |
| 5 | 3 | 3 | 4 | 3 | 2 | 6818.0978 | 6818.1045 | -0.0066 |
| 4 | 4 | 0 | 3 | 3 | 1 | 6832.1016 | 6832.0912 | 0.0103 |
| 5 | 4 | 2 | 4 | 4 | 1 | 6937.9644 | 6937.9568 | 0.0075 |
| 6 | 0 | 6 | 5 | 1 | 5 | 7219.5581 | 7219.5465 | 0.0116 |
| 6 | 1 | 6 | 5 | 0 | 5 | 7219.9054 | 7219.8953 | 0.0101 |
| 5 | 3 | 3 | 4 | 2 | 2 | 7242.3084 | 7242.3133 | -0.0049 |
| 4 | 2 | 2 | 3 | 1 | 3 | 7328.5702 | 7328.5706 | -0.0004 |
| 6 | 1 | 5 | 5 | 2 | 4 | 7667.0922 | 7667.0927 | -0.0005 |
| 6 | 2 | 5 | 5 | 2 | 4 | 7670.2697 | 7670.2642 | 0.0055 |
| 6 | 2 | 5 | 5 | 1 | 4 | 7685.1367 | 7685.1322 | 0.0044 |
| 4 | 3 | 2 | 3 | 0 | 3 | 7761.8811 | 7761.8799 | 0.0012 |

### Frequency list of the G^-^g^+^/cc/G^+^ conformer of methyl α-d-altropyranoside (1)

**Table S8.** Measured (_obs_) and calculated frequencies (_calc_) of the observed rotational transitions for the conformer G^-^g^+^\cc\G^+^, along with their residuals ()

| ***J’*** | ***K_a_*’** | ***K_c_’*** | ***J’’*** | ***K_a_’’*** | ***K_c_’’*** | ****_obs_/MHz** | ****_calc_/MHz** | **Δ**/MHz** |
| --- | --- | --- | --- | --- | --- | --- | --- | --- |
| 6 | 5 | 1 | 6 | 4 | 2 | 2068.2195 | 2068.2282 | -0.0086 |
| 7 | 5 | 3 | 7 | 4 | 4 | 2105.5009 | 2105.5068 | -0.0059 |
| 6 | 5 | 2 | 6 | 4 | 3 | 2112.3998 | 2112.4018 | -0.0019 |
| 8 | 5 | 4 | 8 | 4 | 5 | 2123.9348 | 2123.9444 | -0.0095 |
| 2 | 0 | 2 | 1 | 1 | 1 | 2449.3512 | 2449.3539 | -0.0027 |
| 2 | 1 | 2 | 1 | 1 | 1 | 2568.5899 | 2568.5925 | -0.0026 |
| 2 | 0 | 2 | 1 | 0 | 1 | 2640.7261 | 2640.7245 | 0.0016 |
| 2 | 1 | 2 | 1 | 0 | 1 | 2759.9644 | 2759.9630 | 0.0014 |
| 2 | 1 | 1 | 1 | 1 | 0 | 2778.3076 | 2778.3094 | -0.0018 |
| 2 | 2 | 1 | 1 | 1 | 0 | 3352.4190 | 3352.4210 | -0.0020 |
| 6 | 4 | 2 | 6 | 1 | 5 | 3483.7766 | 3483.7840 | -0.0073 |
| 2 | 2 | 0 | 1 | 1 | 1 | 3490.0063 | 3490.0060 | 0.0002 |
| 3 | 0 | 3 | 2 | 1 | 2 | 3776.6861 | 3776.6900 | -0.0039 |
| 3 | 1 | 3 | 2 | 1 | 2 | 3834.8294 | 3834.8274 | 0.0020 |
| 3 | 0 | 3 | 2 | 0 | 2 | 3895.9340 | 3895.9286 | 0.0054 |
| 3 | 1 | 3 | 2 | 0 | 2 | 3954.0696 | 3954.0660 | 0.0036 |
| 3 | 2 | 2 | 2 | 2 | 1 | 4010.1634 | 4010.1754 | -0.0120 |
| 3 | 2 | 1 | 2 | 2 | 0 | 4124.4188 | 4124.4223 | -0.0034 |
| 3 | 1 | 2 | 2 | 1 | 1 | 4142.6318 | 4142.6341 | -0.0022 |
| 4 | 2 | 2 | 3 | 3 | 1 | 4454.7933 | 4454.8070 | -0.0137 |
| 3 | 1 | 2 | 2 | 0 | 2 | 4576.4418 | 4576.4480 | -0.0062 |
| 3 | 2 | 2 | 2 | 1 | 1 | 4584.3010 | 4584.2870 | 0.0140 |
| 3 | 2 | 2 | 2 | 1 | 2 | 4898.8693 | 4898.8624 | 0.0069 |
| 3 | 2 | 1 | 2 | 1 | 2 | 5045.8283 | 5045.8357 | -0.0074 |
| 4 | 0 | 4 | 3 | 1 | 3 | 5063.5369 | 5063.5401 | -0.0032 |
| 5 | 3 | 3 | 4 | 4 | 0 | 5078.1746 | 5078.1801 | -0.0054 |
| 4 | 1 | 4 | 3 | 1 | 3 | 5087.1375 | 5087.1375 | 0.0000 |
| 4 | 0 | 4 | 3 | 0 | 3 | 5121.6767 | 5121.6775 | -0.0007 |
| 4 | 1 | 4 | 3 | 0 | 3 | 5145.2826 | 5145.2749 | 0.0076 |
| 3 | 2 | 1 | 2 | 0 | 2 | 5165.0650 | 5165.0743 | -0.0093 |
| 3 | 3 | 1 | 2 | 2 | 0 | 5214.5004 | 5214.5080 | -0.0076 |
| 3 | 3 | 1 | 2 | 2 | 1 | 5247.2291 | 5247.2345 | -0.0054 |
| 5 | 2 | 4 | 4 | 3 | 1 | 5253.8872 | 5253.8918 | -0.0045 |
| 3 | 3 | 0 | 2 | 2 | 1 | 5254.0023 | 5254.0032 | -0.0008 |
| 4 | 2 | 3 | 3 | 2 | 2 | 5320.8229 | 5320.8229 | 0.0000 |
| 4 | 3 | 2 | 3 | 3 | 1 | 5396.9427 | 5396.9429 | -0.0001 |
| 4 | 1 | 3 | 3 | 1 | 2 | 5468.8925 | 5468.8944 | -0.0019 |
| 4 | 2 | 2 | 3 | 2 | 1 | 5544.8884 | 5544.8927 | -0.0043 |
| 4 | 2 | 3 | 3 | 1 | 2 | 5762.4828 | 5762.4758 | 0.0070 |
| 5 | 1 | 4 | 4 | 2 | 2 | 6076.8996 | 6076.9034 | -0.0038 |
| 4 | 2 | 2 | 3 | 1 | 2 | 6133.5114 | 6133.5190 | -0.0076 |
| 5 | 0 | 5 | 4 | 1 | 4 | 6320.6119 | 6320.6252 | -0.0132 |
| 5 | 1 | 5 | 4 | 1 | 4 | 6329.1755 | 6329.1772 | -0.0016 |
| 5 | 0 | 5 | 4 | 0 | 4 | 6344.2204 | 6344.2226 | -0.0022 |
| 5 | 1 | 5 | 4 | 0 | 4 | 6352.7963 | 6352.7746 | 0.0216 |
| 5 | 1 | 4 | 4 | 2 | 3 | 6447.9556 | 6447.9466 | 0.0090 |
| 4 | 3 | 2 | 3 | 2 | 1 | 6487.0296 | 6487.0286 | 0.0009 |
| 4 | 3 | 1 | 3 | 2 | 1 | 6531.4714 | 6531.4742 | -0.0027 |
| 5 | 2 | 4 | 4 | 2 | 3 | 6611.5102 | 6611.5164 | -0.0061 |
| 4 | 3 | 2 | 3 | 2 | 2 | 6634.0072 | 6634.0020 | 0.0052 |
| 4 | 3 | 1 | 3 | 2 | 2 | 6678.4468 | 6678.4475 | -0.0007 |
| 5 | 1 | 4 | 4 | 1 | 3 | 6741.5211 | 6741.5280 | -0.0068 |
| 5 | 3 | 3 | 4 | 3 | 2 | 6743.8903 | 6743.9008 | -0.0104 |
| 5 | 4 | 2 | 4 | 4 | 1 | 6755.7185 | 6755.7240 | -0.0054 |
| 5 | 4 | 1 | 4 | 4 | 0 | 6764.5114 | 6764.5147 | -0.0033 |
| 6 | 3 | 3 | 5 | 4 | 2 | 6779.7166 | 6779.7060 | 0.0106 |
| 5 | 3 | 2 | 4 | 3 | 1 | 6855.6786 | 6855.6658 | 0.0128 |
| 5 | 2 | 4 | 4 | 1 | 3 | 6905.0989 | 6905.0977 | 0.0011 |
| 5 | 2 | 3 | 4 | 2 | 2 | 6942.1407 | 6942.1233 | 0.0174 |
| 4 | 4 | 1 | 3 | 3 | 0 | 7054.7497 | 7054.7436 | 0.0060 |
| 4 | 4 | 0 | 3 | 3 | 0 | 7055.9041 | 7055.8949 | 0.0091 |
| 4 | 4 | 1 | 3 | 3 | 1 | 7061.5307 | 7061.5123 | 0.0184 |
| 4 | 4 | 0 | 3 | 3 | 1 | 7062.6811 | 7062.6636 | 0.0175 |
| 4 | 3 | 1 | 3 | 1 | 2 | 7120.1043 | 7120.1004 | 0.0038 |
| 6 | 0 | 6 | 5 | 1 | 5 | 7562.4626 | 7562.4639 | -0.0012 |
| 6 | 1 | 6 | 5 | 1 | 5 | 7565.3550 | 7565.3542 | 0.0008 |
| 6 | 0 | 6 | 5 | 0 | 5 | 7571.0106 | 7571.0159 | -0.0052 |
| 6 | 1 | 6 | 5 | 0 | 5 | 7573.9063 | 7573.9062 | 0.0001 |
| 5 | 2 | 3 | 4 | 1 | 3 | 7606.7377 | 7606.7478 | -0.0101 |
| 5 | 3 | 3 | 4 | 2 | 2 | 7686.0196 | 7686.0367 | -0.0170 |
| 4 | 3 | 2 | 3 | 0 | 3 | 7756.1762 | 7756.1743 | 0.0019 |
| 6 | 1 | 5 | 5 | 2 | 4 | 7805.1562 | 7805.1457 | 0.0104 |
| 5 | 3 | 2 | 4 | 2 | 2 | 7842.2594 | 7842.2472 | 0.0121 |
| 6 | 2 | 5 | 5 | 2 | 4 | 7882.1069 | 7882.1127 | -0.0057 |
| 6 | 1 | 5 | 5 | 1 | 4 | 7968.7059 | 7968.7155 | -0.0096 |

### Frequency list of the Tg^+^/cc/G^+^ conformer of methyl α-d-altropyranoside (1)

**Table S9*.*** Measured (_obs_) and calculated frequencies (_calc_) of the observed rotational transitions for the conformer Tg^+^\cc\G^+^, along with their residuals ()

| *J’* | *K_a_*’ | *K_c_’* | *J’’* | *K_a_’’* | *K_c_’’* | **_obs_/MHz | **_calc_/MHz | Δ**/MHz |
| --- | --- | --- | --- | --- | --- | --- | --- | --- |
| 6 | 3 | 4 | 6 | 1 | 5 | 2285.9147 | 2285.9261 | -0.0114 |
| 5 | 4 | 2 | 5 | 3 | 3 | 2324.8128 | 2324.8054 | 0.0074 |
| 6 | 4 | 3 | 6 | 3 | 4 | 2358.4954 | 2358.4988 | -0.0034 |
| 2 | 1 | 2 | 1 | 1 | 1 | 2409.9015 | 2409.9122 | -0.0107 |
| 7 | 4 | 4 | 7 | 3 | 5 | 2439.4991 | 2439.5100 | -0.0109 |
| 2 | 0 | 2 | 1 | 0 | 1 | 2499.0632 | 2499.0620 | 0.0011 |
| 2 | 1 | 1 | 1 | 1 | 0 | 2651.6496 | 2651.6512 | -0.0015 |
| 2 | 1 | 2 | 1 | 0 | 1 | 2686.9746 | 2686.9818 | -0.0071 |
| 7 | 1 | 6 | 7 | 0 | 7 | 2690.4851 | 2690.4799 | 0.0052 |
| 8 | 5 | 4 | 8 | 4 | 5 | 2938.6570 | 2938.6594 | -0.0024 |
| 6 | 5 | 2 | 6 | 4 | 3 | 2954.1901 | 2954.1895 | 0.0006 |
| 5 | 5 | 0 | 5 | 4 | 1 | 2966.4393 | 2966.4383 | 0.0010 |
| 2 | 2 | 1 | 1 | 1 | 0 | 3482.8612 | 3482.8599 | 0.0013 |
| 3 | 0 | 3 | 2 | 1 | 2 | 3493.5336 | 3493.5298 | 0.0037 |
| 3 | 1 | 3 | 2 | 1 | 2 | 3597.0846 | 3597.0808 | 0.0038 |
| 2 | 2 | 0 | 1 | 1 | 1 | 3635.4494 | 3635.4491 | 0.0003 |
| 3 | 0 | 3 | 2 | 0 | 2 | 3681.4491 | 3681.4496 | -0.0005 |
| 3 | 1 | 3 | 2 | 0 | 2 | 3784.9977 | 3785.0007 | -0.0029 |
| 3 | 2 | 2 | 2 | 2 | 1 | 3796.1663 | 3796.1700 | -0.0037 |
| 3 | 2 | 1 | 2 | 2 | 0 | 3910.8888 | 3910.8905 | -0.0017 |
| 3 | 1 | 2 | 2 | 1 | 1 | 3954.2710 | 3954.2663 | 0.0046 |
| 4 | 1 | 3 | 3 | 2 | 2 | 4548.7949 | 4548.8121 | -0.0172 |
| 3 | 2 | 2 | 2 | 1 | 1 | 4627.3847 | 4627.3788 | 0.0059 |
| 4 | 0 | 4 | 3 | 1 | 3 | 4721.2221 | 4721.2294 | -0.0073 |
| 4 | 1 | 4 | 3 | 1 | 3 | 4769.3723 | 4769.3757 | -0.0034 |
| 4 | 0 | 4 | 3 | 0 | 3 | 4824.7763 | 4824.7805 | -0.0041 |
| 4 | 1 | 4 | 3 | 0 | 3 | 4872.9460 | 4872.9268 | 0.0192 |
| 4 | 2 | 3 | 3 | 2 | 2 | 5036.4630 | 5036.4603 | 0.0026 |
| 4 | 3 | 2 | 3 | 3 | 1 | 5111.9949 | 5111.9961 | -0.0011 |
| 4 | 3 | 1 | 3 | 3 | 0 | 5142.9201 | 5142.9251 | -0.0050 |
| 4 | 1 | 3 | 3 | 1 | 2 | 5221.9470 | 5221.9246 | 0.0224 |
| 5 | 2 | 3 | 4 | 3 | 2 | 5221.8925 | 5221.8767 | 0.0158 |
| 4 | 2 | 2 | 3 | 2 | 1 | 5272.4556 | 5272.4712 | -0.0156 |
| 3 | 2 | 1 | 2 | 0 | 2 | 5324.3533 | 5324.3471 | 0.0061 |
| 3 | 3 | 1 | 2 | 2 | 0 | 5469.7613 | 5469.7569 | 0.0043 |
| 3 | 3 | 0 | 2 | 2 | 1 | 5506.9062 | 5506.8995 | 0.0067 |
| 4 | 2 | 3 | 3 | 1 | 2 | 5709.5604 | 5709.5728 | -0.0123 |
| 5 | 0 | 5 | 4 | 1 | 4 | 5909.4717 | 5909.4732 | -0.0015 |
| 5 | 1 | 5 | 4 | 1 | 4 | 5929.4535 | 5929.4575 | -0.0040 |
| 5 | 1 | 4 | 4 | 2 | 3 | 5949.3501 | 5949.3652 | -0.0151 |
| 5 | 0 | 5 | 4 | 0 | 4 | 5957.6205 | 5957.6195 | 0.0009 |
| 5 | 1 | 5 | 4 | 0 | 4 | 5977.6073 | 5977.6038 | 0.0034 |
| 5 | 2 | 4 | 4 | 2 | 3 | 6256.8755 | 6256.8727 | 0.0028 |
| 5 | 3 | 3 | 4 | 3 | 2 | 6391.5936 | 6391.5949 | -0.0013 |
| 5 | 4 | 2 | 4 | 4 | 1 | 6397.0016 | 6397.0008 | 0.0008 |
| 5 | 4 | 1 | 4 | 4 | 0 | 6402.9464 | 6402.9227 | 0.0237 |
| 5 | 1 | 4 | 4 | 1 | 3 | 6437.0105 | 6437.0134 | -0.0028 |
| 5 | 3 | 2 | 4 | 3 | 1 | 6488.2646 | 6488.2893 | -0.0246 |
| 5 | 2 | 3 | 4 | 2 | 2 | 6620.2591 | 6620.2679 | -0.0087 |
| 4 | 3 | 2 | 3 | 2 | 1 | 6670.8656 | 6670.8625 | 0.0031 |
| 5 | 2 | 4 | 4 | 1 | 3 | 6744.5173 | 6744.5209 | -0.0035 |
| 6 | 2 | 4 | 5 | 3 | 3 | 6759.6560 | 6759.6432 | 0.0128 |
| 4 | 3 | 1 | 3 | 2 | 2 | 6853.6641 | 6853.6546 | 0.0094 |
| 6 | 0 | 6 | 5 | 1 | 5 | 7073.8398 | 7073.8361 | 0.0036 |
| 6 | 1 | 6 | 5 | 1 | 5 | 7081.5586 | 7081.5565 | 0.0021 |
| 6 | 0 | 6 | 5 | 0 | 5 | 7093.8183 | 7093.8204 | -0.0021 |
| 6 | 1 | 6 | 5 | 0 | 5 | 7101.5476 | 7101.5408 | 0.0068 |
| 6 | 1 | 5 | 5 | 2 | 4 | 7288.5920 | 7288.5977 | -0.0057 |
| 4 | 4 | 1 | 3 | 3 | 0 | 7425.9725 | 7425.9727 | -0.0002 |
| 4 | 4 | 0 | 3 | 3 | 1 | 7432.1699 | 7432.1596 | 0.0102 |
| 6 | 2 | 5 | 5 | 2 | 4 | 7455.9761 | 7455.9687 | 0.0074 |
| 4 | 3 | 1 | 3 | 1 | 2 | 7526.7646 | 7526.7670 | -0.0024 |
| 6 | 1 | 5 | 5 | 1 | 4 | 7596.1061 | 7596.1052 | 0.0009 |
| 6 | 3 | 4 | 5 | 3 | 3 | 7658.9684 | 7658.9593 | 0.0091 |
| 6 | 5 | 2 | 5 | 5 | 1 | 7673.8091 | 7673.8151 | -0.0059 |
| 6 | 5 | 1 | 5 | 5 | 0 | 7674.7609 | 7674.7633 | -0.0023 |
| 6 | 4 | 3 | 5 | 4 | 2 | 7692.6519 | 7692.6526 | -0.0007 |
| 6 | 2 | 5 | 5 | 1 | 4 | 7763.4727 | 7763.4761 | -0.0034 |
| 5 | 3 | 3 | 4 | 2 | 2 | 7789.9834 | 7789.9862 | -0.0027 |
| 6 | 2 | 4 | 5 | 2 | 3 | 7929.3466 | 7929.3615 | -0.0148 |

# Crystal structure of methyl α-d-altropyranoside (1)

Crystals were grown by vapor diffusion using Acetonitrile as solvent and *n*-hexane as precipitant. Additionally, a seed crystal was added which was obtained during synthesis.

The data collection for compound **1** was performed with a STOE Imaging Plate Diffraction System (IPDS)2 using CuKα radiation (λ = 1.54184). The structure was solved with SHELXT^[12]^ and refined with SHELXL^[14]^ using Least Squares minimisation. A numerical absorption correction was applied using programs X-RED and X-SHAPE of the program package X-Area (T_min/max_: 0.7995/0.9824).^[15]^ All non-hydrogen atoms were refined with anisotropic displacement parameters.

The C-H and O-H hydrogen atoms were located in difference map but were positioned with idealized geometry (methyl and O-H H atoms allowed to rotate but not to tip) and were refined isotropically with Uiso(H) = 1.2 Ueq(C) (1.5 for methyl H atoms) using a riding model. The asymmetric unit consists of two crystallographically independent molecules.

Because no strong anomalous scattering atoms are present and Mo radiation was used the absolute configuration cannot be determined but was selected according to the known absolute configuration of the starting material.

An ORTEP plot can be found in Figure S19 and Tables with selected crystal data and details of the structure determination can be found in Table S10.

CCDC-2417766 contain the supplementary crystallographic data for this paper. These data can be obtained free charge from the Cambridge Crystallographic Data Centre via http://www.ccdc.cam.ac.uk/data_request/cif.


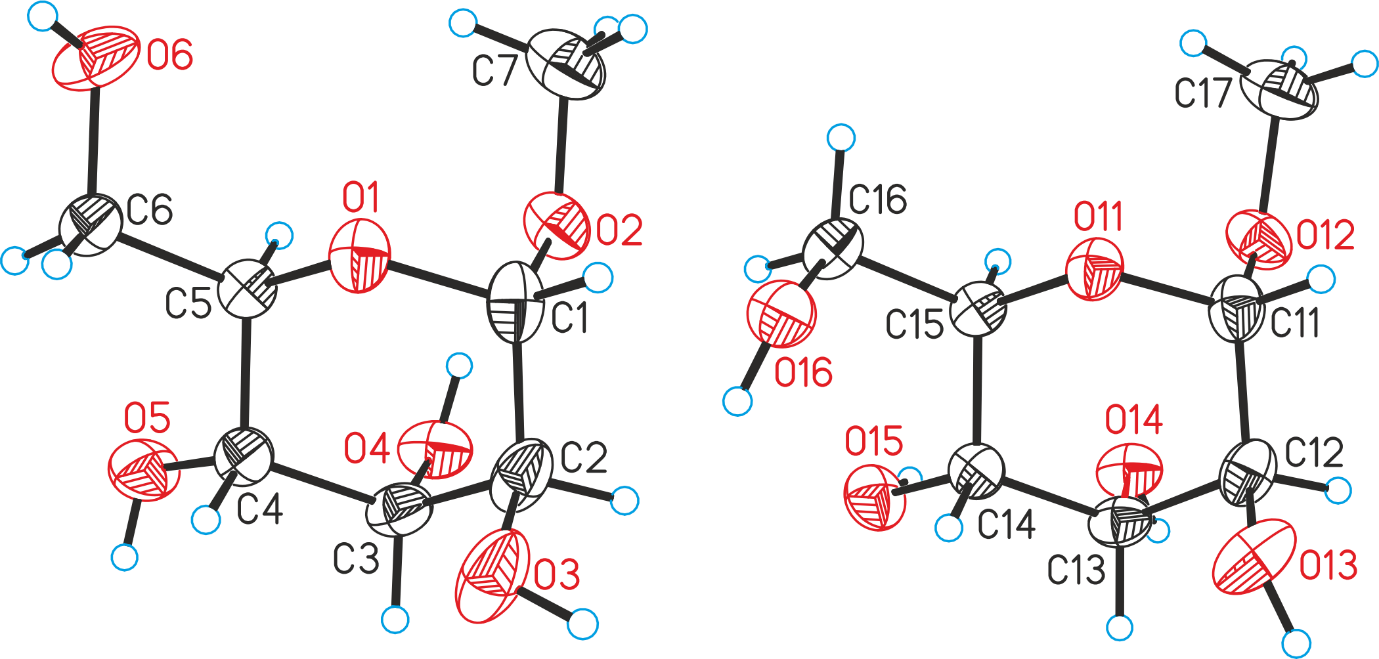


**Figure S19.** Crystal structure of the two crystallographically independent molecules in compound **1** with labeling and displacement ellipsoids drawn at the 50% probability level.

**Table S10.** Selected crystal data and results of the structure refinement for compound methyl α-d-altropyranoside (**1**).

| Empirical formula | C_7_H_14_O_6_ |
| --- | --- |
| Formula weight | 194.18 |
| Temperature/K | 200(2) |
| Crystal system | monoclinic |
| Space group | *P*2_1_ |
| a/Å | 7.7198(3) |
| b/Å | 13.9115(4) |
| c/Å | 8.1686(3) |
| α/° | 90 |
| β/° | 93.606(3) |
| γ/° | 90 |
| Volume/Å^3^ | 875.52(5) |
| Z | 4 |
| ρ_calc_g/cm^3^ | 1.473 |
| μ/mm^‑1^ | 0.130 |
| Crystal size/mm^3^ | 0.2 × 0.3 × 0.4 |
| Θ range for data collection/° | 2.50 to 28.0 |
| Index ranges | -9 ≤ h ≤ 10, -18 ≤ k ≤ 18, -10 ≤ l ≤ 10 |
| Reflections collected | 13330 |
| Independent reflections | 4227 |
| R_int._ | 0.0356 |
| Reflections with [I>=2σ (I)] | 3883 |
| Parameters | 245 |
| Goodness-of-fit on F^2^ | 1.071 |
| Final R indexes [I>=2σ (I)] | R_1_ = 0.0289, wR_2_ = 0.0720 |
| Final R indexes [all data] | R_1_ = 0.0325, wR_2_ = 0.0733 |
| Largest diff. peak/hole / e Å^-3^ | 0.125/-0.136 |

# NMR studies

All NMR spectra were recorded on a Bruker AvanceNeo 500 spectrometer (^1^H NMR: 500 MHz, 298 K, ^13^C NMR: 125 MHz, 298 K). For the NMR measurements TMS was used as standard, except for measurements in D_2_O for which DSS (sodium trimethylsilylpropanesulfonate) was used. For data representation the chemical shift, the multiplicity (s = singlet, d = doublet, t = triplet, q = quartet, m = multiplet), and the coupling constants in Hertz (Hz) are given. For the full assignment common 2D techniques (^1^H-^1^H COSY, ^1^H-^13^C HSQC, ^1^H-^13^C HMBC) were used. The coupling constants were measured only from ^1^H NMR spectra as these typically have lower errors than 2D techniques like COSY-DQF. However, ^1^H NMR spectra of methyl α-d-altropyranoside (**1**) were processed by apodization (Exponential: ‑0.5 Hz, Gaussian: 0.3 Hz) to increase the resolution of the spectrum. As the pyranose signals of methyl α-d-altroside **1** were strongly overlapped by hydroxy signals in DMSO-*d*_6_ (Figure S26, S27) and THF-d_8_ (Figure S32, S33), a drop of D_2_O was added to the NMR sample. Indeed, this masked the hydroxy groups in DMSO-d_6_ without affecting either the chemical shift or the ring coupling constants (Figure S29). Thus, a more precise determination of the coupling constants was allowed. However, adding a drop of D_2_O to **1** in THF-d_8_ led to a slight change of the chemical shift (Figure S35). Consequently, this spectrum was not considered as a source of coupling constants.

For synthetic procedures, please see our previous publication on the synthesis of altrobiosides by Lütjohann et al.^[9]^

### Methyl α-d-altropyranoside (1)

^1^H NMR (500 MHz, D_2_O, 298K, DSS, ppm): δ = 4.66 (d, ^3^*J*_1,2_ = 1.9 Hz 1H, H-1), 3.87-3.93 (m, 3H, H-3, H-5, H-6), 3.86 (dd, ^3^*J*_1,2_ = 1.9 Hz, ^3^*J*_2,3_ = 4.3 Hz, 1H, H-2), 3.83 (dd, ^3^*J*_3,4_ = 3.5 Hz, ^3^*J*_4,5_ = 8.9 Hz, 1H, H-4), 3.76 (dd, ^2^*J*_6a,6b_ = 12.1 Hz, ^3^*J*_5,6a_ = 6.4 Hz, 1H, H-6), 3.40 (s, 3H, OCH_3_); ^13^C NMR (125.8 MHz, D_2_O; 298K, DSS, ppm): δ 103.5 (1C, C-1), 72.8 (1C, C-5), 72.5 (1C, C-2), 72.2 (1C, C-3), 67.2 (1C, C-4), 63.8 (1C, C-6), 58.0 (1C, OCH_3_); ^1^H NMR (500 MHz, methanol-d_4_, 298K, TMS, ppm): δ = 4.58 (s, 1H, H*-*1), 3.84 (dd, ^2^*J*_6a,6b_ = 11.5 Hz, ^3^*J*_5,6a_ = 2.4 Hz, 1H, H-6b), 3.71-3.77 (m, 2H, H-3, H‑5), 3.78 (dd, ^3^*J*_1,2_ = 1.6 Hz, ^3^*J*_2,3_ = 3.9 Hz, 1H, H‑2), 3.74 (dd, ^3^*J*_3,4_ = 3.3, ^3^*J*_4,5_ = 9.5 Hz, 1H, H-4), 3.71 (dd, ^3^*J*_6b,6a_ = 11.5 Hz, ^3^*J*_5,6b_ = 5.7 Hz, 1H, *H-*6a), 3.39 (s, 3H, OCH_3_); ^13^C NMR: (125.8 MHz, methanol-d_4_, 298K, TMS, ppm):  δ =102.82 (C-1), 72.39 (C*-*5), 71.45 (C-2), 70.53 (C-3), 65.83 (C-4), 63.20 (C-6), 55.66 (OCH_3_) ppm; ^1^H NMR (500 MHz, DMSO-d_6_, 298K, TMS, ppm): δ = 5.04 (d, ^3^J = 3.9 Hz, 1H, OH), 4.47 (m, 1H, OH), 4.39 (m, 2H, H*-*1, OH), 4.20 (d, ^3^*J* = 5.1 Hz, 1H, OH), 3.5-3.65 (m, 5H, H-2, H-3, H-4, H-5, H-6b), 3.46 (dt, ^2^*J*_6a,6b_ = 12.5, ^3^*J*_5,6_ = 6.3 Hz, ^3^*J*_6,OH_ = 6.3 Hz, 1H, H-6a), 3.23 (s, 3H, OCH_3_); ^13^C NMR: (125.8 MHz, DMSO-d_6_, 298K, TMS, ppm):  δ =101.1 (C-1), 70.2 (C*-*5), 69.9 (C-2), 69.5 (C-3), 64.3 (C-4), 61.1 (C-6), 54.2 (OCH_3_); ^1^H NMR (500 MHz, DMSO-d_6_ + 1 drop D_2_O, 298K, TMS, ppm): δ = 4.39 (s, 1H, H*-*1), 3.6-3.63 (m, 3H, H-3, H-5, H-6b), 3.60 (dd, ^3^*J*_1,2_ = 1.9 Hz, ^3^*J*_2,3_ = 4.2 Hz, 1H, H-2), 3.53 (dd, ^3^*J*_2,3_ = 3.5 Hz, ^3^*J*_4,5_ = 8.9 Hz, 1H, H‑4), 3.45 (dd, ^2^*J*_6a,6b_ = 12.3, ^3^*J*_5,6_ = 6.8 Hz, 1H, H-6a), 3.22 (s, 3H, OCH_3_); ^1^H NMR (500 MHz, THF-d_8_, 298K, TMS, ppm): δ = 4.40 (dd, ^3^*J*_1,2_ = 1.7 Hz, ^4^*J*_2,3_ = 0.8 Hz 1H, H*-*1), 4.22 (d, ^3^*J* = 5.3 Hz, 1H, OH), 3.63 (m, 1H, H-6), 3.58 (ddd, ^3^*J*_1,2_ = 1.7 Hz, ^3^*J*_2,3_ = 3.7 Hz, ^3^*J*_2,OH_ = 5.4 Hz, 1H, H-2), 3.49-3.56 (m, 4H, H-3, H-4, H-5, H-6), 3.46 (m, 1H, OH), 3,41 (m, 1H, OH), 3.31 (d, ^3^*J* = 7.7 Hz, 1H, OH), 3.23 (s, 3H, OCH_3_); ^13^C NMR: (125.8 MHz, THF-*d*_8_, 298K, TMS, ppm):  δ = 102.9 (C-1), 72.3 (C-3 or C-5), 71.1 (C-2), 70.7 (C-4), 65.7 (C-3 or C-5), 63.4 (C-6), 55.0 (OCH_3_); HRMS (ESI): *m/z* calc for C_7_H_14_O_6_+Na^+^: 217.0688 [M+Na]^+^; found 217.0681.

### *O*-(2,3,4,6-Tetra-*O*-acetyl-α-d-altropyranosyl)trichloroacetimidate (2)

^1^H NMR (500 MHz, CDCl_3_, 298K, TMS, ppm): δ = 8.74 (s, 1H, C=N–H), 6.21 (s, 1H, H-1), 5.37 (dd ∼ t, ^3^*J*_2,3_ = 3.3 Hz, ^3^*J*_3,4_ = 3.3 Hz, 1H, H-3), 5.24 (dd, ^3^*J*_3,4_ = 3.3 Hz, ^3^*J*_4,5_ = 10.2 Hz, 1H, H-4), 5.14 (dd, ^3^*J*_1,2_ = 1.0 Hz, ^3^*J*_2,3_ = 3.4 Hz, 1H, H-2), 4.52 (ddd, ^3^*J*_4,5_ = 10.2 Hz, ^3^*J*_5,6_ = 5.0 Hz, ^3^*J*_5,6_ = 2.3 Hz 1H, H-5), 4.30 (dd, ^2^*J*_6a,6b_ = 12.3 Hz, ^3^*J*_5,6a_ = 5.0 Hz, 1H, H-6a), 4.21 (dd, ^2^*J*_6a,6b_ = 12.3 Hz, ^3^*J*_5,6b_ = 2.3 Hz, 1H, H-6b), 2.19, 2.16, 2.08, 2.04, (each s, each 3H, 4 C(O)CH_3_); ^13^C NMR (125.8 MHz, CDCl_3_, 298K, TMS, ppm): δ = 170.7, 169.7, 169.4, 169.1 (each 1C, 4 C(O)), 160.0 (1C, C=NH), 93.7 (1C, C-1), 90.9 (1C, CCl_3_) 68.2 (1C, C-2), 66.7 (1C, C-5), 66.5 (1C, C-3), 64.4 (1C, C-4), 62.2 (1C, C-6), 20.8 (2C, 2 C(O)*C*H_3_), 20.7, 20.6, (each 1C, 2 C(O)*C*H_3_); HRMS (ESI): *m/z* calc for C_16_H_20_Cl_3_NO_10_+Na^+^: 514.0045 [M+Na]^+^; found: 514.0038.

### *O*-(2,3,4,6-Tetra-*O*-acetyl-β-d-altropyranosyl)trichloroacetimidate (3)

^1^H NMR (500 MHz, CDCl_3_, 298K, TMS, ppm): δ = 8.64 (s, 1H, C=N–H), 6.47 (d, ^3^*J*_1,2_ = 2.8 Hz, 1H, H-1), 5.54 (dd, ^3^*J*_2,3_ = 8.1 Hz, ^3^*J*_3,4_ = 3.3 Hz, 1H, H-3), 5.42 (dd, ^3^*J*_1,2_ = 2.8 Hz, ^3^*J*_2,3_ = 8.1 Hz, 1H, H-2), 5.40 (dd, ^3^*J*_3,4_ = 3.3 Hz, ^3^*J*_4,5_ = 5.0 Hz, 1H, H-4), 4.34 (m, 2H, H-6), 4.25 (m, 1H, H-5), 2.11, 2.09, 2.09, 2.08 (each s, each 3H, 4 C(O)CH_3_); ^13^C NMR (125.8 MHz, CDCl_3_^,^ 298K, TMS, ppm): δ = 170.5 (1C, C(O)), 169.7 (2C, 2 C(O)), 169.5 (1C, C(O)), 160.9 (1C, C=NH), 94.5 (1C, C-1), 90.5 (1C, CCl_3_), 73.8 (1C, C-5), 67.0 (1C, C-4), 66.9 (1C, C-2), 65.6 (1C, C-3), 63.3 (1C, C-6), 20.8 (2C, 2 C(O)*C*H_3_), 20.7, 20.6 (each 1C, 2 C(O)*C*H_3_); HRMS (ESI): *m/z* calc for C_16_H_20_Cl_3_NO_10_+Na^+^: 514.0045 [M+Na]^+^; found: 514.0040.

## NMR spectra of methyl α-d-altropyranoside (1)

**Figure S20.** ^1^H NMR of **1** in methanol-d_4_. (500 MHz, 298 K).

**Figure S21.** ^1^H NMR of **1** in methanol-d_4_. (500 MHz, 298 K) with apodization (Exponential: ‑0.5 Hz, Gaussian: 0.3 Hz).

**Figure S22.** ^13^C NMR of **1** in methanol-d_4_. (125 MHz, 298 K).

**Figure S23.** ^1^H NMR of **1** in D_2_O. (500 MHz, 298 K).

**Figure S24.** ^1^H NMR of **1** in D_2_O. (500 MHz, 298 K) with apodization (Exponential: ‑0.5 Hz, Gaussian: 0.3 Hz).

**Figure S25.** ^13^C NMR of **1** in D_2_O. (125 MHz, 298 K).

**Figure S26.** ^1^H NMR of **1** in DMSO-d_6_. (500 MHz, 298 K).

**Figure S27.** ^1^H NMR of **1** in DMSO-d_6_ (500 MHz, 298 K) with apodization (Exponential: ‑0.5 Hz, Gaussian: 0.3 Hz).

**Figure S28.** ^13^C NMR of **1** in DMSO-d_6_ (125 MHz, 298 K).

**Figure S29.** The ^1^H NMR of **1** in DMSO-d_6_. (500 MHz, 298 K) is displayed above. The ^1^H NMR of **1** in DMSO-*d*_6_ with a drop of D_2_O to mask the OH-groups, is shown below.

**Figure S30.** ^1^H NMR of **1** in DMSO-d_6_ + 1 drop D_2_O (500 MHz, 298 K).

**Figure S31.** ^1^H NMR of **1** in DMSO-d_6_ + 1 drop D_2_O (500 MHz, 298 K) with apodization (Exponential: ‑0.5 Hz, Gaussian: 0.3 Hz).

**Figure S32.** ^1^H NMR of **1** in THF-d_8_ (500 MHz, 298 K).

**Figure S33.** ^1^H NMR of **1** in THF-d_8_ (500 MHz, 298 K) with apodization (Exponential: ‑0.5 Hz, Gaussian: 0.4 Hz).

**Figure S34.** ^13^C NMR of **1** in THF-d_8_ (125 MHz, 298 K).

**Figure S35.** The ^1^H NMR of **1** in THF-d_8_ (500 MHz, 298 K) is displayed above. The ^1^H NMR of **1** in THF-*d*_8_ with a drop of D_2_O to mask the hydroxy groups, is shown below.

## NMR spectra of *O*-(2,3,4,6-Tetra-*O*-acetyl-d-altropyranosyl) trichloroacetimidates 2 and 3

**Figure S36.** ^1^H NMR of **2** in CDCl_3_ (500 MHz, 298 K).

**Figure S37.** ^13^C NMR of **2** in CDCl_3_ (125 MHz, 298 K).

**Figure S38.** ^1^H NMR of **3** in CDCl_3_ (500 MHz, 298 K).

**Figure S39.** ^13^C NMR of **3** in CDCl_3_ (125 MHz, 298 K).

# Concluding remarks on the spectroscopic results in light of the anomeric effect

By employing orthogonal spectroscopic methods we were able to disentangle intra- and intermolecular effects on the conformational properties of methyl α-d-altropyranoside (**1**). In the gas phase, intramolecular forces dominate: Rotational spectroscopy revealed three hydrogen-bonding motifs that differ only in the orientation of the 5-hydroxymethyl group, while the pyranose ring consistently adopts the ^4^*C*_1_ chair conformation. In the crystal, intermolecular forces like hydrogen bonds and van der Waals interactions dominate. As a result, **1** again adopts the ^4^*C*_1_ conformation but only G^+^ (also called *gg*) and G^-^ (also called *gt*) rotamers of the 5-hydroxymethyl group are observed, whereas the T (also called *tg*) rotamer is absent. In solution, methyl altroside **1** exhibits a true conformational equilibrium, with minor populations of the ^1^*C*_4_ chair, which is detected in polar solvents (up to 8 % in D_2_O) but is absent in less polar media. This is in line with our previous report on a *N*-Boc-protected β-xyloside^[6]^ and can be rationalized by the solvent-dependent anomeric effect, which stabilizes an axial aglycone in apolar solvents but favors an equatorial orientation in polar ones (Figure S40).


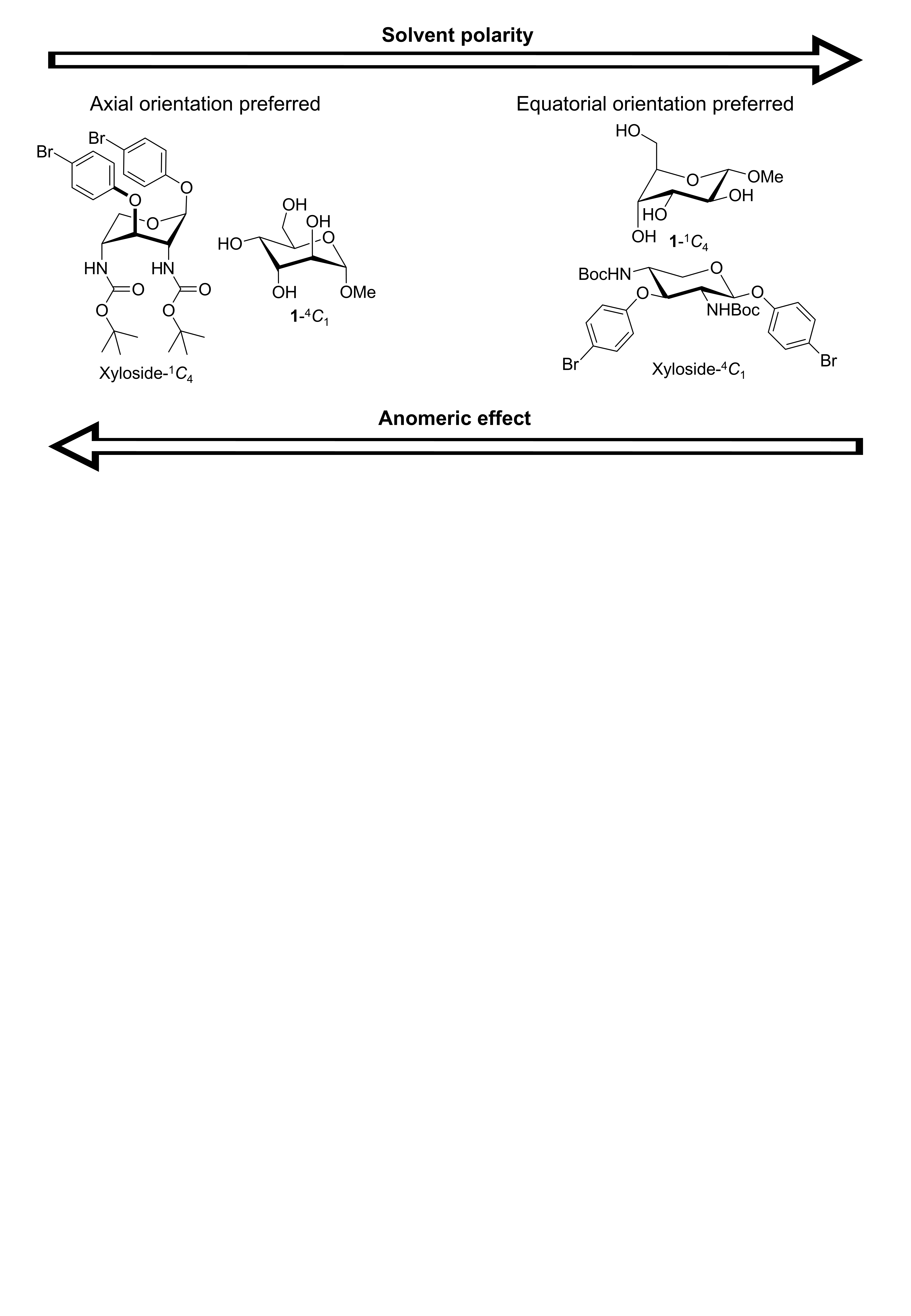


**Figure S40.** Our NMR data show that the solvent-dependent anomeric effect shapes the pyranose conformation of the altropyranoside studied in this account and the previously investigated xylopyranoside.^[6]^ In polar solvents the anomeric effect is weakened, therefore steric interactions dominate, and conformers with equatorial anomeric substituents are favored. On the other hand, in apolar solvents the anomeric effect is stronger and enforces an axial orientation of the anomeric substituent.

# References

[1] a) F. Neese, *J. Comput. Chem.* **2022**, *44*, 381–396; b) F. Neese, *Wiley Interdiscip. Rev.-Comput. Mol. Sci.* **2025**, *15*, e70019.

[2] B. de Souza, *Angew. Chem. Int. Ed.* **2025**, e202500393.

[3] a) V. Barone, M. Cossi, *J. Phys. Chem. A* **1998**, *102*, 1995–2001. b) F. Weigend, R. Ahlrichs, *Phys. Chem. Chem. Phys*. **2005**, *7*, 3297−3305; c) S. Grimme, J. Antony, S. Ehrlich, H. Krieg, *J. Chem. Phys*. **2010**, *132*, 154104; d) S. Grimme, S. Ehrlich, L. Goerigk, *J. Comput. Chem.* **2011**, *32*, 1456–1465.

[4] a) A. D. Becke, *Phys. Rev. A* **1988**, *38*, 3098–3100; b) C. Lee, W. Yang, R. G. Parr, *Phys. Rev. B* **1988**, *37*, 785–789; c) F. Weigend, M. Häser, H. Patzelt, R. Ahlrichs, *Chem. Phys. Lett*. **1998**, *294*, 143–152; d) A. D. Becke, *J. Chem. Phys.* **1992**, *96*, 2155–2160; e) E. Caldeweyher, S. Ehlert, A. Hansen, H. Neugebauer, S. Spicher, C. Bannwarth, S. Grimme, *J. Chem. Phys.* **2019**, *150*, 154122.

[5] a) T. H. Dunning, *J. Chem. Phys.* **1989**, *90*, 1007–1023; b) F. Neese, A. Hansen, D. G. Liakos, *J. Chem. Phys.* **2009**, *131*, 064103; c) F. Neese, A. Hansen, F. Wennmohs, S. Grimme, *Acc. Chem. Res.* **2009**, *42*, 641–648; d) D. G. Liakos, A. Hansen, F. Neese, *J. Chem. Theory Comput.* **2010**, *7*, 76–87; e) C. Riplinger, F. Neese, *J. Chem. Phys.* **2013**, *138*, 034106; f) C. Riplinger, B. Sandhoefer, A. Hansen, F. Neese, *J. Chem. Phys.* **2013**, *139*, 134101; g) C. Riplinger, P. Pinski, U. Becker, E. F. Valeev, F. Neese, *J. Chem. Phys.* **2016**, *144*, 024109; h) Y. Guo, C. Riplinger, U. Becker, D. G. Liakos, Y. Minenkov, L. Cavallo, F. Neese, *J. Chem. Phys.* **2018**, *148*, 011101; i) M. Garcia‐Ratés, F. Neese, *J. Comput. Chem.* **2020**, *41*, 922–939; j) M. Garcia‐Ratés, U. Becker, F. Neese, *J. Comput. Chem.* **2021**, *42*, 1959–1973.

[6] S. O. Jaeschke, T. K. Lindhorst, A. Auer, “Between Two Chairs: Combination of Theory and Experiment for the Determination of the Conformational Dynamics of Xylosides” *Chem. Eur. J.* **2022**, *28*, e202201544.

[7] a) J.P. Perdew, J. A. Chevary, S. H. Vosko, K. A. Jackson, M. R. Pederson, D. J. Singh, C. Fiolhais, *Phys. Rev. B* **1992**, *46*, 6671–6687; b) J. P. Perdew, K. Burke, M. Ernzerhof, *Phys. Rev. Lett.* **1996**, *77*, 3865–3868; c) J. P. Perdew, M. Ernzerhof, K. Burke, *J. Chem. Phys.* **1996**, *105*, 9982–9985; d) J. Tao, J. P. Perdew, V. N. Staroverov, G. E. Scuseria, *Phys. Rev. Lett.* **2003**, *91*, 146401; e) J. P. Perdew, J. Tao, V. N. Staroverov, G. E. Scuseria, *J. Chem. Phys.* **2004**, *120*, 6898–6911; f) F. Jensen, *Theor. Chem. Acc.* **2010**, *126*, 371–382.

[8] a) C. Møller, M. S. Plesset, *Phys. Rev.* **1934**, *46*, 618–622; b) R. A. Kendall, T. H. Dunning, R. J. Harrison, *J. Chem. Phys.* **1992**, *96*, 6796–6806; c) D. E. Woon, T. H. Dunning, *J. Chem. Phys.* **1993**, *98*, 1358–1371.

[9] C. Lütjohann, C. Näther, T. K. Lindhorst, *Carbohydr. Res*. **2024**, *544*, 109228.

[10] D. Schmitz, V. A. Shubert, T. Betz, M. Schnell, *J. Mol. Spectrosc.* **2012**, 280, 77–84.

[11] C. Pérez, A. Krin, A. L. Steber, J. C. López, Z. Kisiel, M. Schnell, *J. Phys. Chem. Lett.* **2015** *7*, 154–160.

[12] H. M. Pickett, *J. Mol. Spectrosc*. **1991**, *148*, 371–377.

[13] G. M. Sheldrick, *Acta Crystallogr*. **2015**, *A71*, 3–8.

[14] G. M. Shledrick, *Acta Crystallogr*. **2015**, *C71*, 3–8.

[15] Stoe & Cie (**2002**). X-RED, X-SHAPE, X-Area*.* Stoe & Cie, Darmstadt, Germany.
